# A Computational Structural Analysis of Host Insertions in the Polyproline Region of the Hepatitis E Virus pORF1 Polyprotein

**DOI:** 10.3390/v18030341

**Published:** 2026-03-10

**Authors:** Nicolas Jeanne, Olivia Paronetto, Chloé Dimeglio, Florence Abravanel, Sébastien Lhomme, Marie Brut, Jacques Izopet

**Affiliations:** 1 Laboratoire de Virologie, Hôpital Purpan, CHU Toulouse, 31300 Toulouse, France; dimeglio.c@chu-toulouse.fr (C.D.); abravanel.f@chu-toulouse.fr (F.A.); lhomme.s@chu-toulouse.fr (S.L.); izopet.j@chu-toulouse.fr (J.I.); 2 Institut Toulousain des Maladies Infectieuses et Inflammatoires (Infinity), UMR 1291 (Institut Natioanl de la Santé Et de la Recherche Médicale), UMR 5051 (Centre National de la Recherche Scientifique), Université de Toulouse, 31024 Toulouse, France; olivia.paronetto@inserm.fr; 3 Laboratoire d’Analyse et d’Architecture des Systèmes—Centre National de la Recherche Scientifique, Université de Toulouse, CNRS, 31400 Toulouse, France; marie.brut@laas.fr

**Keywords:** hepatitis E virus, insertions, replication rates, Alphafold, molecular dynamics, hydrogen bonds, capping pore

## Abstract

Hepatitis E virus, a single-stranded positive-sense RNA virus, is the causative agent of acute viral hepatitis in humans and can lead to chronic infection in immunocompromised individuals. In this setting, strains containing host genome insertions within the polyproline region (PPR) of the pORF1 polyprotein were characterized and shown to display an increased replication rate across all systems. Using in silico modeling of pORF1 across 25 strains, combined with molecular dynamics (MD) simulations, we explored the structural variations caused by these insertions to investigate potential mechanisms underlying the increased replication rate compared to wild-type (WT) strains. Our results showed that the insertions neither induced structural organization within the PPR nor altered its intrinsically disordered nature. MD simulations further demonstrated that the overall stability of pORF1 remained unchanged in strains with insertions compared to WT strains. On the other hand, MD analyses revealed that strains with insertions exhibited an increased number of hydrogen bonds between the PPR and two other domains of pORF1: the MetY domain and the RNA-dependent RNA polymerase (RdRp). The stability of the MetY domain of the strains in the presence of host insertion events was higher than in the WT strains. These additional hydrogen bonds could position the MetY domain and the RdRp closer together, potentially promoting more efficient viral RNA synthesis. Validation of this hypothesis will require experimental structural studies, as well as computational modeling of the proposed dodecameric pORF1 structure.

## 1. Introduction

With 19.47 million cases of acute hepatitis E virus (HEV) infections and 3450 deaths in 2021, HEV is a major public health concern (WHO 2025, https://www.who.int accessed on 15 February 2026). While many infections are asymptomatic and self-limiting, pregnant women and patients with pre-existing liver disease are at risk of developing acute liver failure. Immunocompromised patients are also susceptible to developing chronic infections [1,2,3] leading to rapid liver fibrosis if left untreated. The international committee on the taxonomy of viruses classified HEV under the *Paslahepevirus balayani* species. Eight genotypes of HEV have been identified, but HEV genotypes 1 to 4 (HEV 1–4) are the most widely spread [4]. HEV-1 and HEV-2 have only been found in humans. Transmission occurs mainly through contaminated water, primarily in developing countries with poor sanitary conditions, where it can lead to waterborne outbreaks. HEV-3 and HEV-4 are zoonotic, with pigs, wild boars, deer and rabbits as their main reservoirs [5].

HEV is a single-stranded, positive-sense RNA virus. The HEV genome, about 7.2 kb long, with a 5′ methylguanylate cap and 3′ polyadenylated extremity, contains three main open reading frames (ORFs). The first, ORF1, encodes a polyprotein (pORF1) composed of several non-structural domains involved in HEV replication, including the MetY domain, the fatty-acid binding domain or metal binding domain (FABD/MBD), the polyproline region (PPR), the Macro domain, the helicase, and the RNA-dependent RNA polymerase (RdRp) [6,7]. The notion that a viral protease cleaves pORF1, as initially suggested during the first annotation of the HEV genome [8], has been reconsidered. The question of a cleavage of pORF1 by a host protease [9,10,11] or not [6,12,13] is still a debated topic in the scientific community. The PPR that contains a proline-rich segment is recognized as an intrinsically disordered region (IDR) [14]. The PPR contributes to viral replication and adaptation. It has been demonstrated that PPR deletion attenuates but does not abolish the virus replication [15,16,17]. Additionally, this region serves as a hotspot for insertion events from the host genome. These genetic events were reported in genotype 3 strains in various subtypes, including 3a, 3e, 3f and 3m [18]. Most strains harboring insertions have been shown to possess a replicative advantage in vitro [19,20,21,22]. Insertions increase the number of post-translational modification sites, such as acetylation, ubiquitination, and phosphorylation, in the PPR, while also enhancing the overall charge of this domain [21,22]. Based on the hypothesis that pORF1 exists in an uncleaved form, we hypothesized that interactions between the PPR and other domains of the polyprotein could influence the viral replication. We investigated whether host insertions in the PPR influence those interactions and could be associated with a replicative advantage. We adopted a structural approach by modeling various pORF1 proteins from HEV strains carrying insertions, along with wild-type (WT) strains. These models were then subjected to molecular dynamics simulations to identify potential conformational changes or interactions between the PPR and other pORF1 domains.

## 2. Materials and Methods

### 2.1. Strains

This study analyzed 25 HEV strains, focusing specifically on the amino acids sequence encoding the pORF1. Nine strains contain insertions, identified by the strain name and the insertion origin, which subtypes are 3a, 3f, 3h and 3m: HEPAC-6 *RNF19A* (MF444145), HEPAC-26 *RPL6* (MF444089), HEPAC-64 *ZNF787* (MF444119), HEPAC-93 *EEF1A1* (MN646692), HEPAC-93 *RNA18SP5* (MN646695), HEPAC-100 *GATM* (MN646689), HEPAC-100 *PEBP1* (MN646696), HEPAC-154 *KIF1B* (MF444083) and Kernow-C1-p6 *RPS17* (JQ679013). The corresponding pORF1 amino acid sequences are available in the File S1. The replication rates of strains with insertions have been examined in prior research [21,22]. For comparisons, we used 16 WT strains (pORF1 amino acid sequences in the File S2), with accession numbers: AB248520, AB291961, AB437318, EU495148, FJ653660, FJ956757, JN837481, JN906974, JQ679014 (Kernow-C1-p1), KT447527, KT447528, KU980235, KY232312, KY780957, MF444031, and MG783569. The subtypes of the WT strains and the strains with insertions were determined through a phylogenetic analysis of the nucleotide sequences and the subtype assignment for each strain is provided in Table S1. Strains carrying insertions and the selected WT strains all belong to the three major clades of HEV genotype 3: 3abk, 3efg, and 3chilm.

### 2.2. pORF1 Structure Modeling

Structure modeling from the amino acid sequence was conducted using AlphaFold2 [23], with computations performed on a High-Performance Computing infrastructure. From the AlphaFold2 predictions, predicted alignment error (PAE) metrics were extracted to assess the consistency of domain predictions [24]. This analysis was carried out using a custom script, Alphafold Metrics Visualisation v1.3.0 (https://github.com/njeanne/alphafold_metrics_visualisation). From the data generated by AlphaFold2, we can extract the predicted local distance difference test (pLDDT) score [25], which reflects the model’s confidence in residue positioning. A low pLDDT score is commonly interpreted as an indicator of low confidence in AlphaFold’s prediction of amino acid positioning. However, a pLDDT score below 50 may also suggest the presence of an intrinsically disordered region (IDR), as reported by Ruff et al. [26]. This analysis was performed using the custom Python script IDR Alphafold v1.1.1 (https://github.com/njeanne/idr_alphafold).

### 2.3. Molecular Dynamics

All atom molecular dynamics (MD) simulations over 1 µs were performed on the predicted structures using Amber22 [27,28,29] and the generalized Born implicit solvent model [30]. Hydrogen atoms generated by AlphaFold were removed using the PyMOL v2.5.0 [31] command action/hydrogen/remove, as Amber cannot directly process them. The FF14SB force field [32] was applied to the protein residues for all 25 systems, with coordinates and topology files created using LeaP (from package AmberTools 20). For each system, an energy minimization was first carried out with the Sander program to remove close contacts. Subsequent MD was then run using the pmemd program to leverage multiple GPUs for efficiency. The systems were heated from 0 to 300° K over 100,000 cycles, using a Langevin thermostat with a collision frequency of 1 ps^−1^. A time step of 2 fs and a nonbonded cutoff of 12 Å were applied, as well as the SHAKE algorithm to constrain all hydrogen-containing bonds. Once the target production temperature was reached, production runs were executed in 17 slices, each consisting of 30 million cycles, culminating in a total of 1 µs of simulation. Finally, trajectory files were merged using CPPTRAJ v5.1.0 [33] to compile the complete trajectory for each system. The strains PDB, topology, coordinates files and the MD configuration files are available in this repository (https://github.com/njeanne/HEV_ORF1_MD_whole_atoms accessed on 30 January 2026).

### 2.4. Molecular Dynamics Trajectories Study

The root mean square deviation (RMSD) quantifies the deviation in backbone atom positions of pORF1 over time by comparing each simulation frame of the MD trajectory to a reference. The root mean square fluctuation (RMSF) quantifies the positional fluctuations of backbone atoms for each residue over the course of the MD simulation. The PPR was excluded from the RMSF analysis owing to substantial variability in the length of its amino acid sequences. These analyses were performed on the 1 µs MD trajectories to evaluate the pORF1 stability across the WT and insertions systems. They were conducted using custom scripts, rms v1.5.0 (https://github.com/njeanne/rms), rms aggregate v1.4.0 (https://github.com/njeanne/rms_aggregate) and rmsf compare v1.0.0 (http://github.com/njeanne/rmsf_compare), developed with pytraj (v2.0.6), a Python package binding to CPPTRAJ program.

For each strain, hydrogen bond formation between the PPR and other pORF1 domains during the MD simulations was analyzed using a custom script trajectories hbonds v1.2.0 built with Pytraj, available in this repository (https://github.com/njeanne/trajectories_hbonds). The results were processed with another custom script, plot hbonds v2.4.0 (http://github.com/njeanne/plot_contacts), to generate graphical representations of the hydrogen bonds of interest. Finally, hydrogen bonds were aggregated by category (WT and insertions) using a third custom script, contacts aggregate v1.0.0 (https://github.com/njeanne/contacts_aggregate), to explore statistical differences in hydrogen bond formation.

### 2.5. Statistical Analysis

The comparison of the aggregated hydrogen bonds by strains with insertions and WT strains was conducted using a non-parametric Mann–Whitney statistical test. For pORF1 domains in which the number of hydrogen bonds with the PPR was significantly increased in strains with insertions, we classified the strains in two groups, strains with insertions and WT strains. In each group, we computed the Manhattan distance of the RMSD values of the strains within the group. For Strain A, the Manhattan distance of the first frame RMSD was computed against all RMSD frames of Strain B. The same procedure was then applied to the second frame RMSD of Strain A, and so on, until all RMSD values across all strains within a group had been compared. Finally, we conducted Mann–Whitney rank tests to evaluate if a significative difference exists between the groups.

## 3. Results

### 3.1. Insertions Origins and Structures

**Figure 1:**
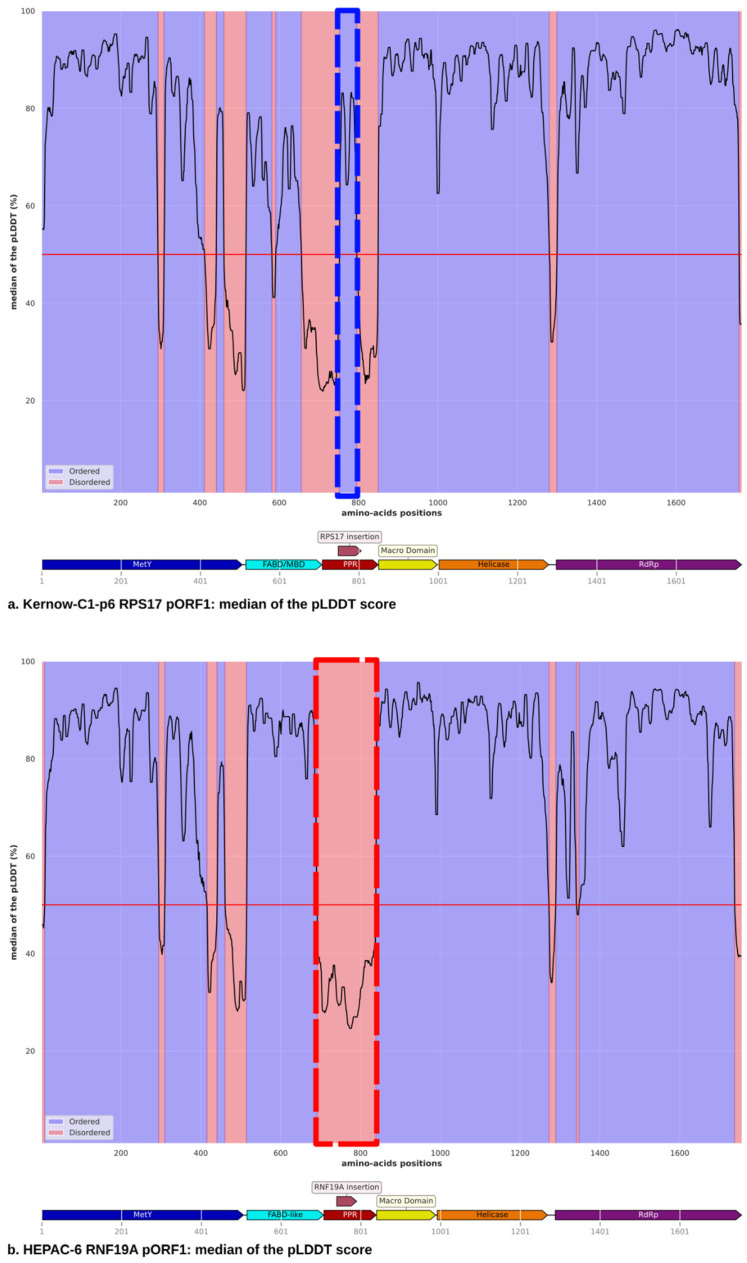
The predicted local distance difference test (pLDDT) score illustrates the intrinsically disordered regions in pORF1, where regions with a pLDDT score < 50% threshold, symbolised by the solid horizontal red line, are highlighted in red, indicating disorder. (**a**) The *RPS17* insertion within the polyproline region (PPR) shows a transition from a disordered state to a more ordered state at the insertion site (marked by a blue dashed line). This suggests that the *RPS17* insertion introduces structural order into what was previously a disordered region. The same pattern exists for the *RPL6* insertion (Figure S2). (**b**) In contrast, the *RNF19A* insertion does not affect the disordered state of the PPR (highlighted by a red dashed line). This behavior is consistent with the six other insertions, none of which alter the intrinsic disorder of the PPR (Figure S2).

The origins of these insertions, previously reported in our earlier study [18], are diverse and are detailed in Table 1. The insertions primarily originate from exons, with some retaining the original ORF and structure of the host protein, such as *RPL6* and *RPS17*, which exhibit an alpha-helix structure. One insertion (*KIF1B*) loses its structure upon insertion, while keeping the same ORF. Others, like *RNF19A*, *EEF1A1*, and *GATM*, lack any initial structure. One insertion derives from the 5′ UTR of the gene (*ZNF787*), another from the 3′ UTR (*PEBP1*), and the last from a pseudogene (*RNA18SP5*), none of which exhibit any structure. The timing of these insertions appears to occur after RNA splicing, as observed in *GATM* and *KIF1B*, where the insertions are found at the junctions between two exons. This observation is consistent with the presence of insertions in the 5′ and 3′ untranslated regions, *ZNF787* and *PEBP1*, respectively, the exonic insertions *RNF19A*, *RPL6*, *EEF1A1, RPS17*, and the *RNA18SP5* pseudogene. The two insertions where the host protein structure is preserved (*RPL6* and *RPS17*) transform portions of the PPR from a disordered to an ordered state (Figure 1a). This contrasts with other insertions where the original sequence ORF is not conserved, resulting in the absence of these structural changes (Figure 1b). Among the insertions studied, only *RPS17* and *RPL6* display defined structures within the PPR. These are among the longest insertions (57 and 48 amino acids, respectively) and originate from ribosomal genes, which are well represented in the reference databases used by AlphaFold2 to construct multiple sequence alignments (MSAs). These factors could contribute to the observed structural predictions. The MSA coverage plots (Figure S1) for these two sequences indeed show a high number of sequences included in the alignment (approximately 2000), although sequence identity remains relatively low. However, a similar number of sequences with comparable identity scores is observed for *ZNF787*, yet no structure is predicted for this insertion (Figure S1). For the *KIF1B* insertion, which also derives from an exonic sequence and retains its original reading frame, AlphaFold2 does not predict any structure. This may be due to its shorter length (25 amino acids), or the structural context of the insertion, which may prevent preservation of the original α-helical conformation. The pLDDT confidence scores were plotted for all the samples full-length pORF1 (Figure S2).

### 3.2. pORF1 Domain Predictions

**Figure 2:**
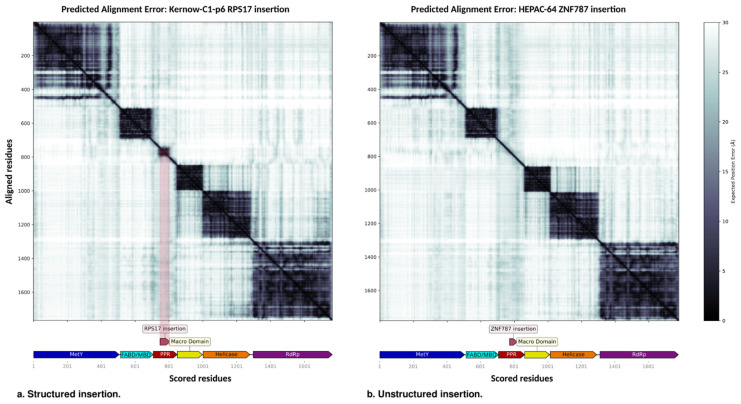
The Predicted Alignment Error (PAE) plot generated from AlphaFold modeling provides insights into the confidence of predicted domain interactions within pORF1. Low PAE values (represented by dark colors) between residue pairs from different domains indicate that AlphaFold predicts their relative positions and orientations with high confidence. High PAE values (shown in light colors) suggest uncertainty in the relative positioning and/or orientations of the domains. A domain map of pORF1 is displayed at the bottom of the figure. (**a**) Strain Kernow-C1-p6 containing the *RPS17* insertion, and similarly for *RPL26* insertion, the insertion shows a structure that was present in the original proteins highlighted in purple. Those structures are not present for the other insertions as shown for example in (**b**) for the strain HEPAC-64 with the *ZNF787* insertion.

The accuracy of AlphaFold model predictions can be evaluated using the PAE, which measures the model’s confidence in the relative positioning of two residues within the predicted structure. PAE is defined as the expected positional error for residue X, measured in Ångströms (Å), assuming the predicted and actual structures were aligned on residue Y. A low PAE between residue pairs X and Y from different domains indicates that AlphaFold predicts their relative positions and orientations with high confidence. Conversely, a high PAE suggests uncertainty in the relative positions and/or orientations of these domains, meaning they should not be interpreted as well-defined in the 3D structure. In the WT strains, the PAE values extracted from the AlphaFold models clearly define the domains identified by Fieulaine et al. and Goulet et al. [6,7], the MetY domain, the FABD/MBD, the Macro domain, the Helicase, and the RdRp (Figure S3). The only exception is the PPR, which is not defined as a domain due to its intrinsically disordered nature. The Macro domain, Helicase, and RdRp exhibit low PAE scores that indicate well-defined relative positioning between these domains (Figure 2). The insertions exhibit similar patterns, with the exception of two: *RPS17* and *RPL6*. These exceptions display structured regions on the PAE plot at the insertion sites. The open reading frame is preserved, and a structure was present in the original proteins. For Kernow-C1-p6 with the *RPS17* insertion (Figure 2a), the five domains of the pORF1 map align with the low-score regions on the PAE heat map. The PPR exhibits high PAE scores, consistent with its classification as an intrinsically disordered region, except for the *RPS17* insertion, which retains the structured region of the original protein. Similarly, for the HEPAC-64 strain with the *ZNF787* insertion (Figure 2b), representative of a structure lacking insertions, the PPR domain map matches the PAE high-score regions. However, since this insertion does not preserve the open reading frame of the original protein, no low PAE scores are observed at the insertion site.

### 3.3. Stability of the pORF1

**Figure 3:**
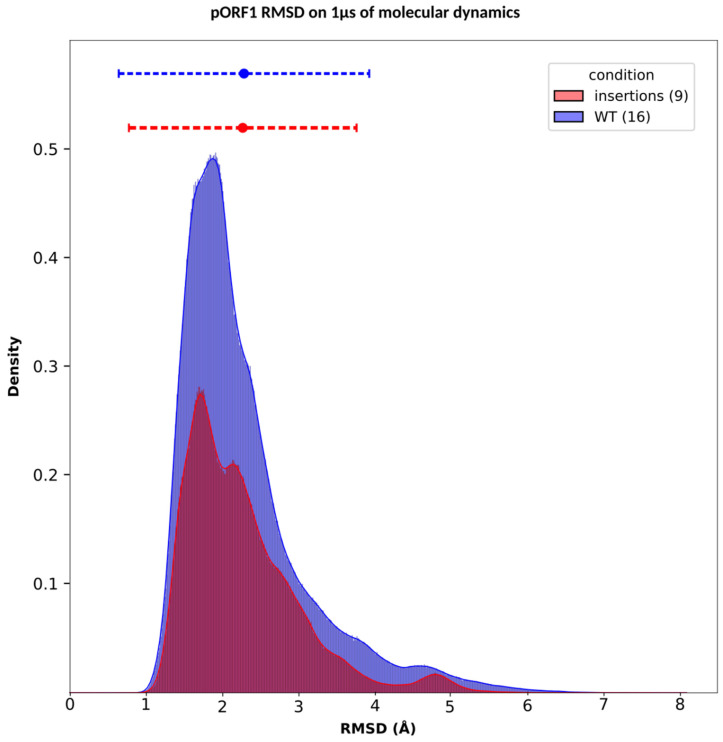
pORF1 aggregated RMSD distribution by group: insertions and WT. The RMSD results over the 1 µs Molecular Dynamics simulation were analyzed by category: blue for WT, and red for insertions. The density histogram of RMSD values shows the distribution of deviations for each category. Mean RMSD values are represented by dots, while the dashed lines indicate the confidence intervals, allowing for a visual assessment of variability and overlap between the groups.

During the simulation, we analyzed the RMSD and the RMSF of pORF1. For the RMSD we initially used the first frame as the reference to evaluate system behavior (Figure S4). Our analysis indicated that the systems reached equilibrium around frame 70,000 on average (280 ns), as evidenced by the small standard deviations of RMSD values calculated from this frame to the final frame (1 µs), Table 2. Based on this, we extracted the MD trajectory from frame 70,000 onward for RMSD computation. To refine our choice of a reference frame, we applied a k-means clustering method to identify the most representative frame from the MD simulation. The k-means algorithm partitions data into clusters by assigning each point to the nearest cluster center, which is initially chosen at random. The cluster centers are then updated as the mean of their assigned points, and the process is repeated until convergence. By grouping similar conformations based on structural descriptors, k-means facilitates the exploration of the conformational space [34]. In our study, the primary goal of clustering was to investigate dominant conformational states along the trajectories. Additionally, using these structures as references for MD analysis provides the advantage of relying on representative, physically meaningful conformations, rather than an arbitrary snapshot (Figure S5). Since the simulation time was consistent across all strains in this study, we aggregated the RMSD data by group (WT and insertions). For each frame, we computed the median RMSD for each group. The aggregated RMSD analysis revealed that all systems remained stable throughout the simulation, with median RMSD values ranging between 1 and 5 Å. When comparing the groups using density histograms of the RMSD, along with mean and confidence interval calculations (Figure 3), no differences were observed between the groups. The confidence intervals for all groups overlapped, indicating similar stability across WT strains and strains with insertions (Table 3). In our study, the RMSD and statistical results for the PPR alone were consistent with those observed in the pORF1 protein. Due to variations in residue numbers among strains, the RMSF analysis was first performed individually for each strain and subsequently grouped into categories defined by the presence of insertions or WT sequences. The grouped RMSF values were compared across the pORF1 domains using the mean and the corresponding 95% confidence intervals, however, no distinctive patterns were observed, as all confidence intervals overlapped (Figure S6).

### 3.4. Hydrogen Bonds Between PPR and the pORF1 Domains

**Figure 4:**
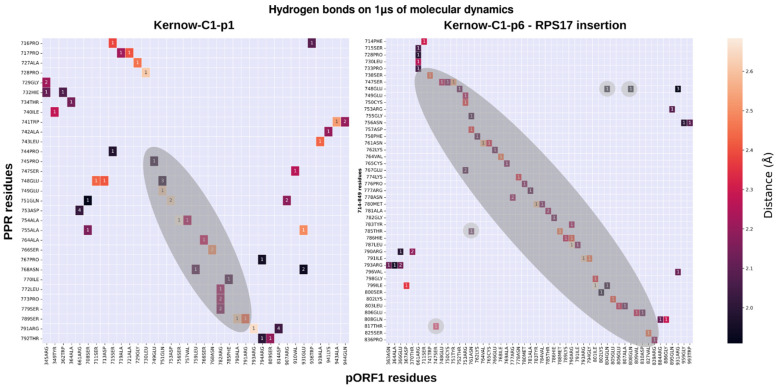
Kernow-C1-p1 WT (**left** panel) and Kernow-C1-p6 with *RPS17* insertion (**right** panel) hydrogen bonds between PPR and pORF1. The darker colors represent close contacts and the lighter colors more distant contacts with the maximal distance set to 3 Å. The number in the squares are the number of atoms in contacts between the 2 residues. The grayed region highlights the contact between the PPR residues and other residues from the same domain. Our interest is focused on the contacts between residues of the PPR and other domains.

**Figure 5:**
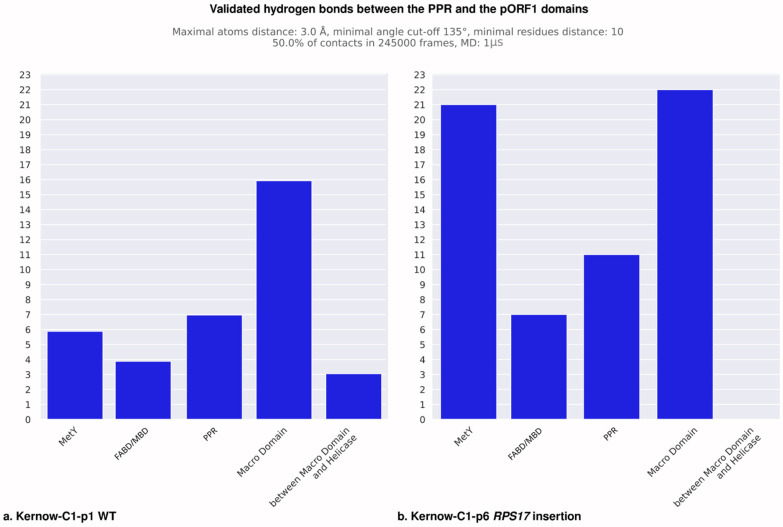
Validated hydrogen bonds between PPR and pORF1 domains for the Kernow-C1 strains without and with insertion. The plots display the number of hydrogen bonds between the PPR and pORF1 domains that meet the criterion of at least 10 residues of separation between contacting residues. (**a**) The count of validated hydrogen bonds for the Kernow-C1-p1 strain without insertions. (**b**) The count of validated hydrogen bonds for the Kernow-C1-p6 strain with the *RPS17* insertion in the PPR. An increase in hydrogen bonds is observed between the PPR and several other domains, including the MetY domain, the PPR itself, and the Macro domain.

**Figure 6:**
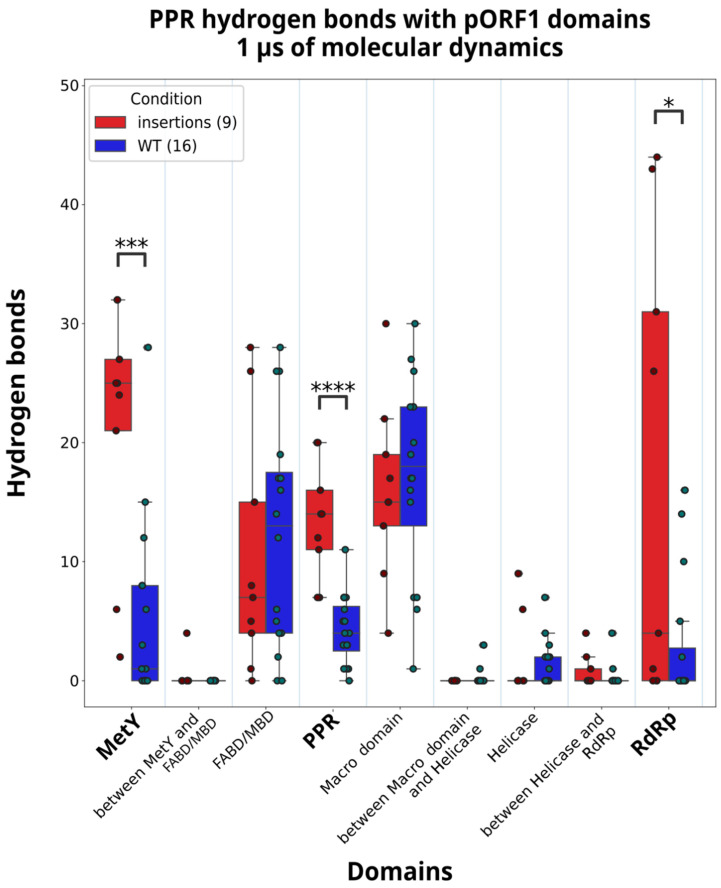
Hydrogen bonds between the PPR and pORF1 domains for the two conditions: insertions and WT. The quartiles of the distributions for the aggregated number of hydrogen bonds between the PPR and pORF1 domains are shown for both conditions: insertions (red) and WT (blue). Results from the Mann–Whitney two-tailed hypothesis test indicating a significant increase in contact numbers for the insertion condition are marked with asterisks (*: *p* ≤ 0.05, ***: *p* ≤ 0.001, ****: *p* ≤ 0.0001), and the relevant domains are highlighted in bold.

To investigate the influence of the insertions in the PPR on the pORF1 conformation, we analyzed the formation of the hydrogen bonds during the MD simulation on 1 µs. Hydrogen bonds are good descriptors for domain–domain interactions, the proteins may employ spatially localized hydrogen bonds to accommodate different functional requirements and structural conformations [35]. We focused on the difference in hydrogen bonds formed on the strains with insertion events and the WT strains. To validate a hydrogen bond, we defined several criteria that must be satisfied. The distance between the donor and acceptor atoms must be less than 3 Å, and the angle formed by the acceptor atom, the donor hydrogen, and the donor atom must exceed 135°. In addition, these conditions must be met in more than 50% of the MD simulation frames. Finally, the distance in residue numbers must be more than 10 in order to discard close contacts residues. The analysis was performed for each strain producing heatmaps of the hydrogen bonds (Figure S7). The Kernow-C1-p1 and Kernow-C1-p6 (Figure 4) show many interactions along the diagonals. These bonds are expected, as they represent intra-PPR hydrogen bonding. The comparison between the Kernow-C1-p1 strain without insertion and the Kernow-C1-p6 strain with the *RPS17* insertion after filtering out the hydrogen bonds with less than 10 amino acids of distance between the PPR amino acid and the pORF1 amino acid (Figure 5) reveals an increase in hydrogen bonds between the PPR and several other domains, including the MetY domain, the PPR itself, and the Macro domain. To generalize the analysis, we aggregated the validated hydrogen bonds of the strains with insertions and the WT strains, calculated the quartiles for each distribution, and examined differences in hydrogen bond number between the PPR and the pORF1 domains for both conditions (Figure 6). In the strains with insertion events, a significant increase in hydrogen bonds was observed between the residues of the PPR itself (*p* = 8.48 × 10^−05^) but also between the PPR and two other domains, the MetY domain (*p* = 9.89 × 10^−04^) and the RdRp (*p* = 0.027).

### 3.5. Localization of Hydrogen Bonds Specific to Strains with Insertions

A Multiple Sequence Alignment of pORF1 was performed on the strains with insertions and on the WT strains. Using the list of validated hydrogen bonds, we mapped these contacts onto the alignment to identify specific hydrogen bonds present in the insertion strains but absent in the WT strains. We also identified contacts common to both groups, with a particular focus on the MetY domain and RdRp domains. Among the strains with insertions, none of the 84 residues forming hydrogen bonds within the MetY domain, were involved in more than three of the nine strains, with a maximum of seven atomic contacts observed at a single position. Alignment of the MetY domain revealed that strains with insertions formed hydrogen bonds predominantly in the 3′ region of the domain, spanning positions 292 to 437 (Figure S8). Among the 82 contacts within the RdRp, a maximum of two out of nine strains shared a given contact position, with up to six atomic contacts at one location. For the RdRp domain alignment, insertion-specific contacts increase in two key regions: from positions 1536 to 1587 at the 5′ end, and from 1653 to 1676 in the central part of the RdRp domain (Figure S9).

### 3.6. RMSD Comparison for the Increased Hydrogen Bond Domains, the MetY and the RdRp, with the PPR

For the regions exhibiting an increased number of hydrogen bonds with the PPR, specifically, the MetY domain and the RdRp, we compared the RMSD Manhattan distances for the strains with insertions and for the WT strains, then we compared the Manhattan distances data sets. No significant difference in stability within the MetY or the RdRp domains between strains with insertions and WT strains were observed.

## 4. Discussion

We first modeled the pORF1 structures with and without host insertions in the PPR. By performing molecular dynamics simulations, we examined whether these insertions conferred increased stability or, conversely, greater flexibility to the pORF1 structure, but we did not observe any significant differences. However, the molecular dynamics analysis revealed an increase in hydrogen bond formation between the PPR-containing insertions and two other pORF1 domains: the MetY and RdRp domains.

To better understand the potential functional implications of these insertions, we examined their origins, which are diverse, ranging from exons and exon junctions to 5′ and 3′ UTRs, and even a pseudogene. In their original contexts, most of these sequences lack a defined structure, and they similarly fail to confer structural organization to pORF1. This suggests that their mechanism of action does not involve stabilizing the PPR into a more rigid or ordered state that could impact the overall conformation of pORF1. Consistently, AlphaFold modeling and pLDDT score analysis reveal that WT strains contain five intrinsically disordered regions, aligning with previous findings [6,7], with the PPR standing out as the most prominent. With the exception of two insertions that retained conserved structures from their origin, the remaining insertions fail to convert the PPR from a disordered to an ordered conformation. This suggests they do not contribute to pORF1 stabilization.

A question we addressed was whether the overall stability of pORF1 changed over time when comparing strains with insertions to the WT strains. To investigate this, we performed 1 µs molecular dynamics simulations and computed the RMSD and the RMSF, using the most representative structure as a reference for each sample. The aggregated RMSD values of the strains with an insertion were compared to the WT ones, revealing no significant differences. These findings suggest that pORF1 stability is not affected by insertion events. Similarly, the RMSF analysis did not reveal any changes in the stability of specific regions of pORF1 when comparing strains with insertions to the WT ones. Furthermore, no consistent pattern indicating altered stability was observed in the PPR region as a result of these insertions. Together, these results support the conclusion that the mechanisms leading to improved replication of hepatitis E viruses do not stem from stabilization or destabilization of pORF1.

We also investigated whether the intrinsically disordered PPR interacts with other pORF1 domains, focusing on differences between strains with insertions and WT strains. To assess this, we analyzed hydrogen bond formation over the course of the 1 µs molecular dynamics simulations. In strains with insertions, the PPR forms a significantly higher number of hydrogen bonds with itself. A closer examination of hydrogen bond localization within the PPR of strains with insertions revealed that these interactions are formed not only between the insertions and the rest of the PPR but also between regions of the PPR outside the insertions. This suggests that the effect is not cumulative due to the increased size of the PPR but rather reflects a rearrangement of the domain.

The PPR also forms an increased number of hydrogen bonds with both the MetY domain and the RdRp (including the adjacent undefined C-terminal region), indicating enhanced interactions between these domains. Analysis of the residues involved in hydrogen bond formation within the MetY domain reveals an increase in interactions toward the C-terminal end. However, no single amino acid is consistently shared across all strains with insertions; at most, a given residue is involved in hydrogen bonding in only three out of the nine strains. For the RdRp, the increase in hydrogen bond formation mostly occurs in the N-terminal region of the domain. Similar to the MetY domain, no specific residue is consistently involved, with any given hydrogen bond observed in no more than two out of the nine strains. The lack of shared specific residues involved in hydrogen bond formation across strains could reflect the variability in the amino acid composition of the PPR. Consequently, PPR residues may interact with different positions within the MetY or RdRp domains depending on the strain. Additionally, hydrogen bond formation is primarily governed by spatial proximity. Because the overall conformation adopted by pORF1 can vary subtly depending on the nature of the insertion, the relative positions of residues also differ between strains, resulting in variability in the specific hydrogen bonds formed.

Duplication events and rearrangements in the PPR have been reported in previous studies [21,36,37]. We therefore performed the same analyses as for all other samples on four strains carrying duplications identified in our laboratory [18]. In these strains, the duplications originated from the PPR region (MN646690 and MN646691), from the PPR and the Macro domain (MF444086) and from the PPR and the RdRp (MF444033). Adding these four strains in the host insertions group yielded results similar to those obtained when comparing the original host insertions group with the WT strains.

Interestingly, structural analysis of non-structural protein 1 (nsp1) from the alphavirus Chikungunya virus has shown that it assembles into a dodecameric, crown-like structure and interacts with the endosomal/lysosomal membrane of the host cell [38,39]. This interaction facilitates the formation of spherules-membrane-bound replication sites that concentrate metabolites and proteins necessary for viral RNA synthesis while helping the virus evade the host immune response. The Chikungunya virus nsp1 dodecamer is composed of three structural regions: the crown, located in the cytoplasmic area; the waist; and the skirt, which is embedded within the spherule structure. The comparison of the Chikungunya virus nsp1 to the HEV MetY domain, or putative capping pore as identified by Goulet et al. [7], suggests it may be identified as a capping pore oligomerizing into a dodecameric structure analogous to its Chikungunya counterpart.

**Figure 7:**
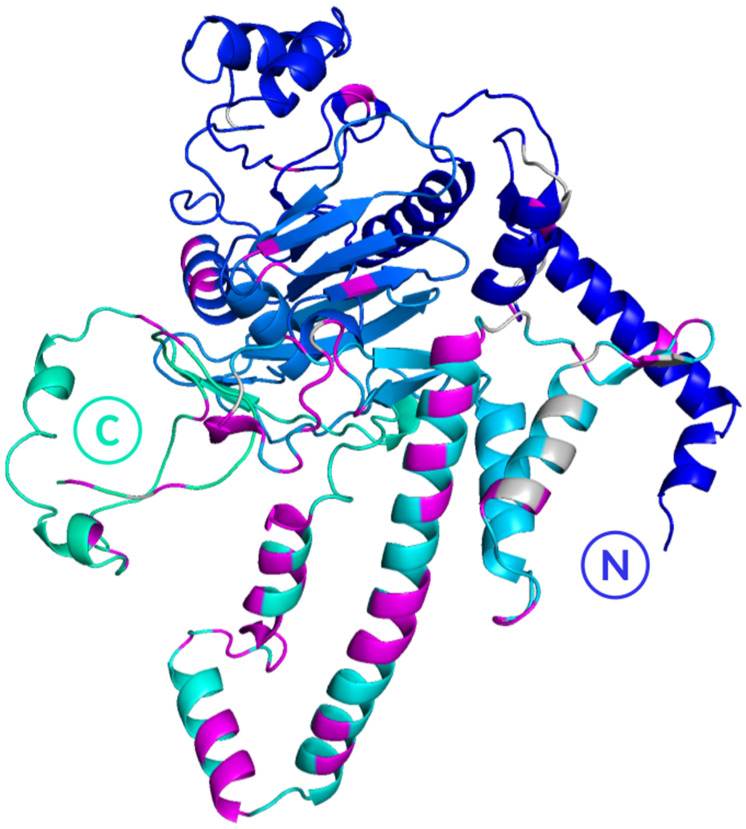
MetY domain residues forming hydrogen bonds with the PPR. The MetY domain, conserved across strains with and without insertions, is colored with a blue-to-cyan gradient from the N-terminal to the C-terminal end (respectively marked with a N and a C). Residues forming hydrogen bonds with the PPR, in at least one strain, are highlighted: grey indicates residues shared between the WT and insertion strains, while magenta marks residues only in insertion strains. These interactions are predominantly concentrated in the C-terminal region of the MetY domain.

Analysis of hydrogen bonds involving residues of the capping pore/MetY domain (Figure 7) revealed that most interactions occur at the C-terminal side, particularly in the waist and skirt regions, consistent with the dodecameric conformation of the Chikungunya virus non-structural protein 1. This observation supports the hypothesis that hydrogen bonding between the PPR and the capping pore/MetY on one hand, and between the PPR and the RdRp on the other, may facilitate the optimal positioning of the polymerase relative to the capping pores and, by extension, to cytoplasmic metabolites thereby enhancing the efficiency of viral RNA synthesis.

To investigate this further, it would be interesting to model a dodecameric conformation of pORF1 in order to analyze the spatial arrangement of its domains constrained by its crown-like architecture, followed by molecular dynamics simulations to assess the stability and interactions among the domains. Such an approach would provide a preliminary computational framework prior to experimental validation. Unfortunately, modeling such a structure is highly memory-demanding and even with Alphafold3 running on NVIDIA A100 GPUs with 80 GB of memory, we were only able to generate a tetrameric pORF1 structure. We examined whether the hydrogen bonds formed between the PPR and these two domains in insertion-containing strains altered the structural stability of the MetY domain and RdRp relative to WT strains. As no difference in stability was observed, we concluded that the increase in hydrogen bonds does not affect the stability of these domains.

As a continuation of this study, it would be of interest to investigate the impact of mutations in the PPR of strains carrying insertions or duplications for which these mutations have been shown to reduce the replication rate [19,21,40]. Molecular modeling, molecular dynamics simulations, and trajectory analyses of strains with and without these mutations are needed to determine whether these mutations also affect hydrogen bond formation between the PPR and MetY or RdRp.

## 5. Conclusions

To summarize, we generated 25 in silico models of the HEV polyprotein pORF1, including strains with insertions in the PPR and WT strains. These models suggest that the insertions neither induce structural organization within the PPR domain, nor shift this region from an intrinsically disordered to an ordered state. Molecular dynamics simulations further indicate that insertions may be associated with increased hydrogen bond formation within the PPR itself, as well as between the PPR and two other pORF1 domains, namely MetY and RdRp. However, these additional interactions in the insertion strains do not appear to substantially affect the structural stability of either the MetY or RdRp domains, nor do they significantly alter the overall stability of pORF1 compared to WT strains.

If HEV were to follow a mechanism similar to that proposed for the alphavirus Chikungunya virus, where the MetY domain forms a crown-like oligomeric structure referred to as a capping pore, such interactions could bring the RdRp into closer proximity with metabolites transiting through the pore, potentially enhancing the efficiency of viral RNA synthesis. Nevertheless, an experimentally resolved structure of pORF1 would be required to validate these computational predictions.

## Figures and Tables

**Table 1:** Description of the insertions in the PPR.

HEV Sequence	Event	Origin	Size (AA)	Replication Rate ^1^
HEPAC-6	Insertion	*RNF19A* Ubiquitin Protein Ligase [Exon]	51	Increased
HEPAC-26	Insertion	*RPL6* Ribosomal Protein L6 [Exon]	48	Similar
HEPAC-64	Insertion	*ZNF787* Zinc Finger 787 [5′ intronic sequence]	45	Increased
HEPAC-93	Insertion	*EEF1A1* Eukaryotic translation Elongation Factor 1 Alpha 1 [Exon]	18	Increased
HEPAC-93	Insertion	*RNA18SP5* 18S Ribosomal Pseudogene 5 [pseudogene]	25	Similar
HEPAC-100	Insertion	*GATM* Glycine Amidinotransferase [2 exons junction]	29	Increased
HEPAC-100	Insertion	*PEBP1* Phosphatidyl Ethanolamine Binding Protein 1 [3′ intronic sequence]	18	Not studied
HEPAC-154	Insertion	*KIF1B* Kinesin Family 1B [2 exons junction]	25	Increased
Kernow-C1-p6	Insertion	*RPS17* Ribosomal Protein S17 [Exon]	57	Increased

^1^ The replication rate comparison is conducted using the wild type (WT) Kernow-C1-p1 strain as the reference [21,22].

**Table 2:** pORF1 mean root mean square deviation (RMSD) values and standard deviations from the 70,000th frame to the final frame (255,000th), using the first frame as the reference.

Strain	Event	RMSD Mean (Å)	RMSD Standard Deviation (Å)
HEPAC-6 *RNF19A*	Insertion	18.93	0.5
HEPAC-26 *RPL6*	Insertion	38.37	0.98
HEPAC-64 *ZNF787*	Insertion	14.91	0.31
HEPAC-93 *EEF1A1*	Insertion	21.51	0.21
HEPAC-93 *RNA18SP5*	Insertion	16.47	0.23
HEPAC-100 *GATM*	Insertion	18.75	0.54
HEPAC-100 *PEPB1*	Insertion	16.37	0.21
HEPAC-154 *KIF1B*	Insertion	17.04	0.4
Kernow-C1-p6 *RPS17*	Insertion	14.41	0.21
AB248520-3e	WT	15.39	0.33
AB291961-3f	WT	16.99	0.2
AB437318-3b	WT	13.81	0.33
EU495148-3f	WT	16.09	0.36
FJ653660-3f	WT	13.81	0.28
FJ956757-3f	WT	14.97	0.35
JN837481-3a	WT	10.28	0.18
JN906974-3f	WT	17.42	0.27
Kernow-C1-p1	WT	18.1	0.27
KT447527-3f	WT	12.24	0.31
KT447528-3a	WT	14.75	0.25
KU980235-3f	WT	12.02	0.22
KY232312-3f	WT	17.81	0.43
KY780957-3h	WT	12.47	0.78
MF444031-3c	WT	11.22	0.97
MG783569-3c	WT	15.56	0.22

**Table 3:** RMSD mean and confidence interval for the insertions and WT strains.

	Mean (Å)	Minimum Confidence Interval (Å)	Maximum Confidence Interval (Å)	Variance (Å)
Insertions	2.267	0.772	3.761	0.581
WT	2.283	0.638	3.927	0.704

## Data Availability

The PDB, MD topology, MD coordinate, and MD configuration files are available in this repository.

## Supplementary Materials

The following supporting information can be downloaded at: https://www.mdpi.com/article/10.3390/v18030341/s1, Table S1: Characteristics of strains and accession numbers; Figure S1: Alphafold2 pORF1 MSA coverage plots for the 9 strains with insertions; Figure S2: Alphafold2 pORF1 pLDDT score; Figure S3: Alphafold3 modeling of Kernow-C1-p6 pORF1 domains with *RPS17* insertion; Figure S4: RMSD with the first frames as references; Figure S5: RMSD with the most representative frame from k-means clustering as reference; Figure S6: RMSF comparison among strains with insertions and WT sequences across the pORF1 domains; Figure S7: Hydrogen bonds between the PPR and the pORF1; Figure S8: Multiple Sequences Alignment of the MetY domain and number of hydrogen bonds with the PPR; Figure S9: Multiple Sequences Alignment of the RdRp and number of hydrogen bonds with the PPR; File S1: The HEV pORF1 amino acids sequences with insertion events fasta file; File S2: The HEV pORF1 Wild Types amino acids sequences fasta file.
