# Supplementary material for: A Computational Structural Analysis of Host Insertions in the Polyproline Region of the Hepatitis E Virus pORF1 Polyprotein
Source: Viruses. 2026 Mar 10;18(3):341. doi: 10.3390/v18030341 (PMC13030434; doi:10.3390/v18030341)
Supplement: Supplementary file 1 [file viruses-18-00341-s001.zip › Supplementary_information.pdf]

Supplementary Table S1. Characteristics of strains and accession numbers.

Sample	Subtype	Clade	Event	Accession Number
HEPAC-64 *ZNF787*	3f	3efg	host insertion	MF444119
HEPAC-93 *EEF1A1*	3f		host insertion	MN646692
HEPAC-93 *RNA18SP5*	3f		host insertion	MN646695
HEPAC-100 *GATM*	3f		host insertion	MN646689
HEPAC 100 *PEBP1*	3f		host insertion	MN646696
HEPAC-154 *KIF1B*	3f		host insertion	MF444083
HEPAC-6 *RNF19A*	3h	3chilm	host insertion	MF444145
HEPAC-26 *RPL6*	3m		host insertion	MF444089
Kernow-C1-p6 *RPS17*	3a	3abk	host insertion	JQ679013
AB248520	3e	3efg	WT	AB248520
AB291961	3f		WT	AB291961
EU495148	3f		WT	EU495148
FJ653660	3f		WT	FJ653660
FJ956757	3f		WT	FJ956757
JN906974	3f		WT	JN906974
KT447527	3f		WT	KT447527
KU980235	3f		WT	KU980235
KY232312	3f		WT	KY232312
MF444031	3c	3chilm	WT	MF444031
MG783569	3c		WT	MG783569
KY780957	3h		WT	KY780957
JN837481	3a	3abk	WT	JN837481
KT447528	3a		WT	KT447528
Kernow-C1-p1	3a		WT	JQ679014
AB437318	3b		WT	AB437318

Supplementary Figure S1. Alphafold2 pORF1 MSA coverage plots for the 9 strains with insertions. The x-axis represents MSA coverage, while the y-axis indicates the amino-acid position within pORF1. Colored lines correspond to sequences from the databases used in the MSA, with color intensity reflecting sequence identity to the query ranging from high confidence (blue) to low confidence (red). Insertions are highlighted with grey dashed boxes.

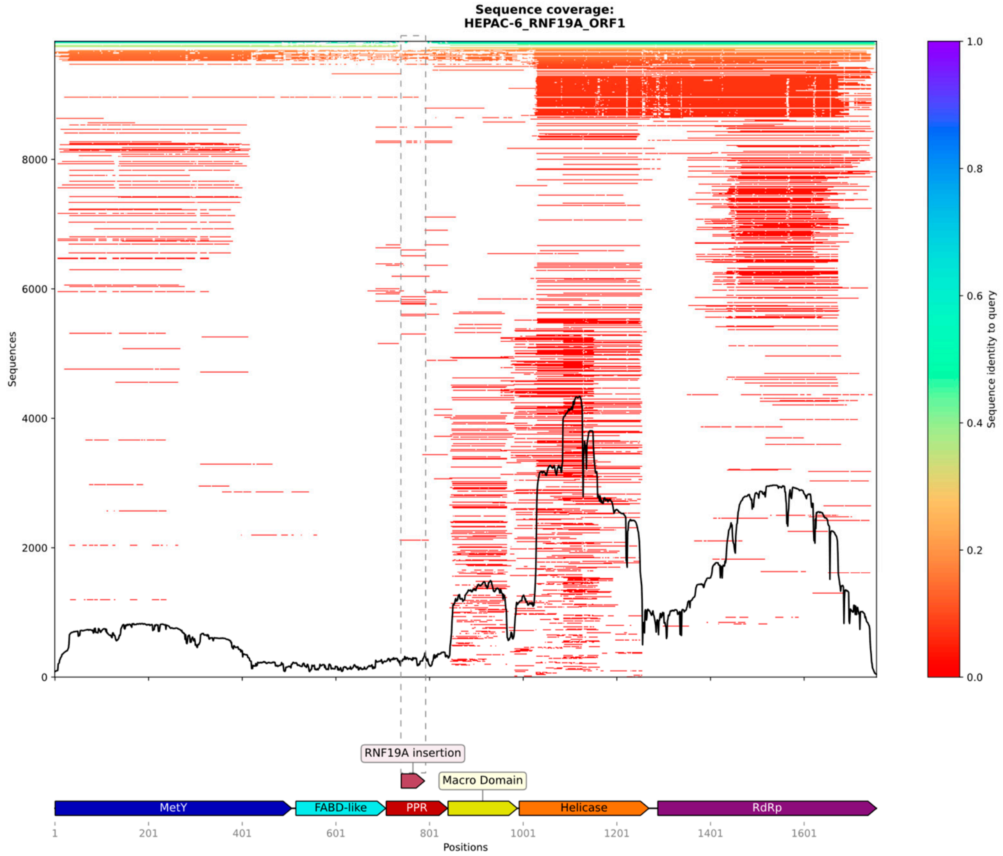

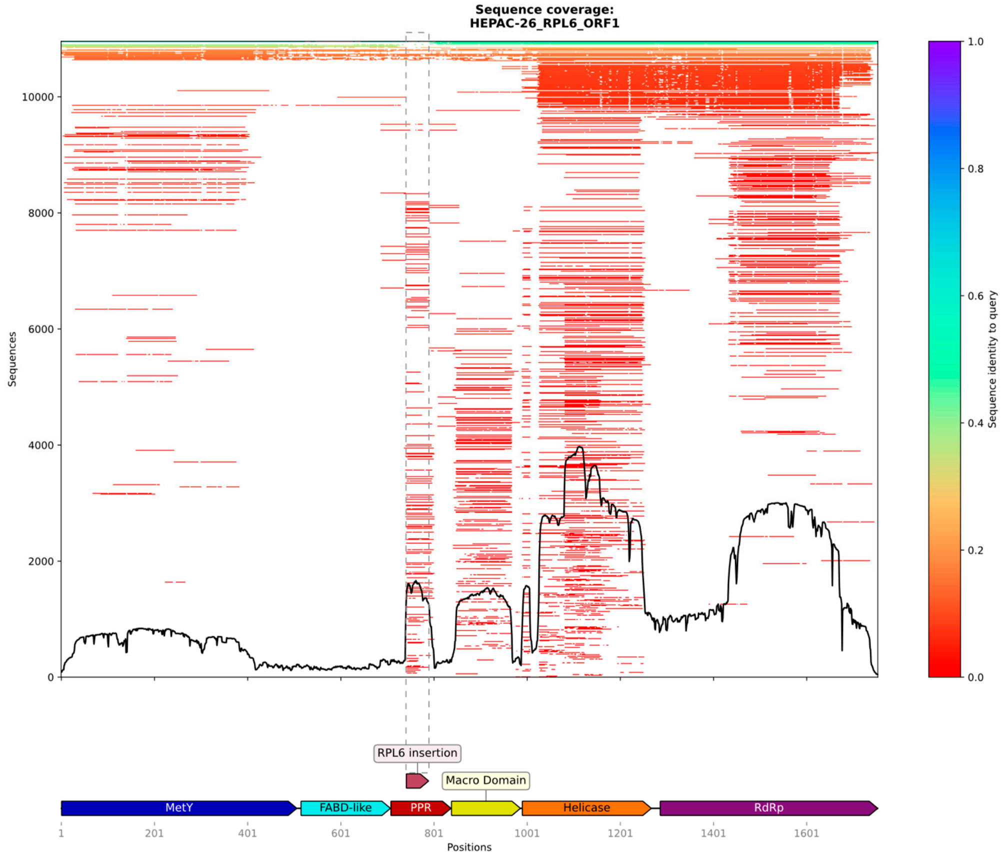

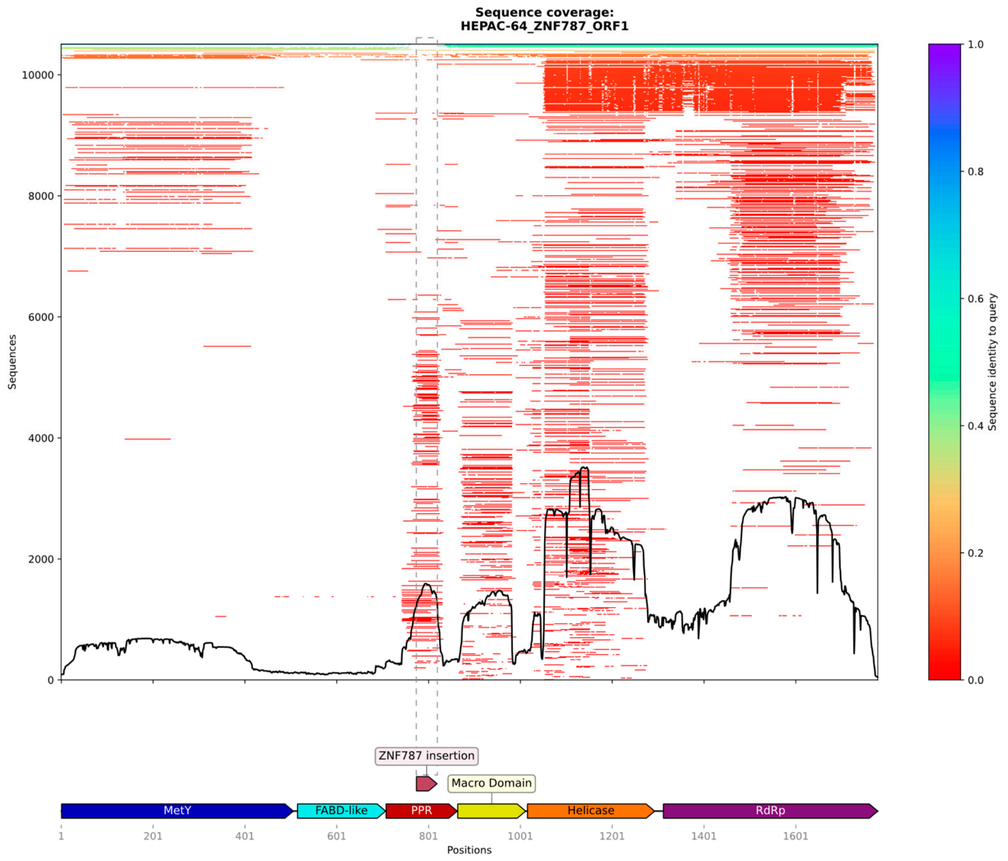
Sequence coverage:
HEPAC-64_ZNF787_ORF1
Sequences
0
2000
4000
6000
8000
10000
Sequence identity to query
0.0
0.2
0.4
0.6
0.8
1.0
ZNF787 insertion
Macro Domain
MetY
FABD-like
PPR
Helicase
RdRp
1
201
401
601
801
1001
1201
1401
1601
Positions

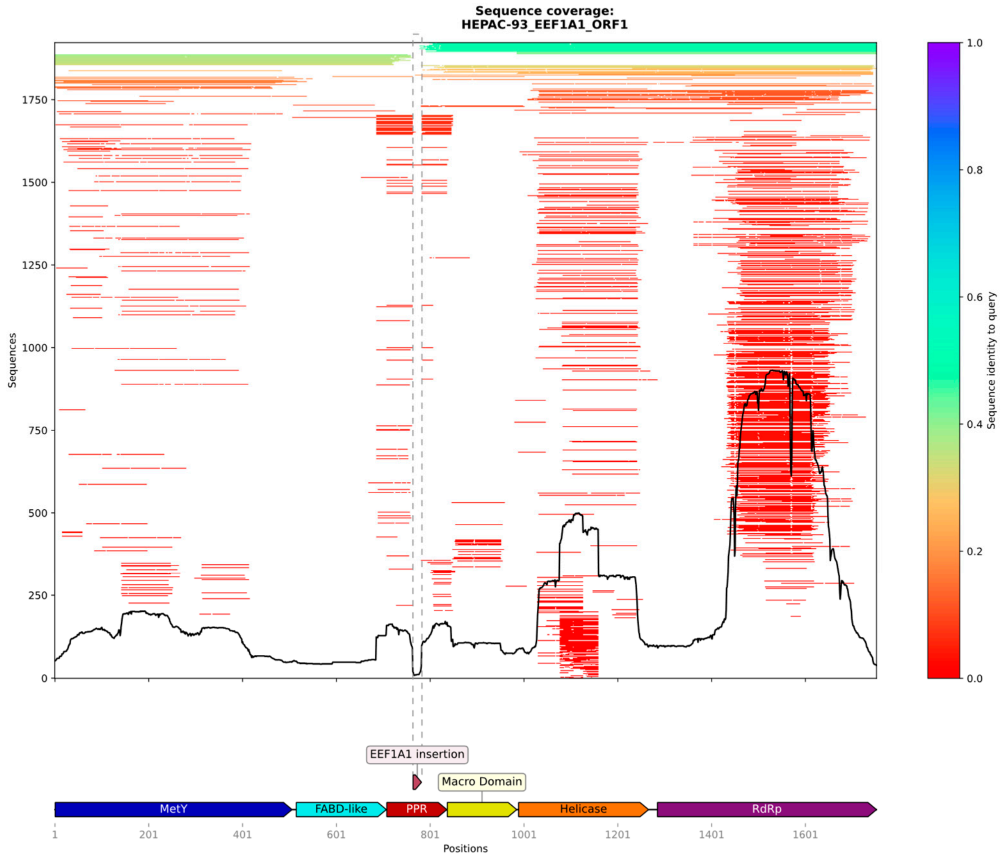
Sequence coverage:
HEPAC-93_EEF1A1_ORF1
Sequences
0
250
500
750
1000
1250
1500
1750
Sequence identity to query
0.0
0.2
0.4
0.6
0.8
1.0
EEF1A1 insertion
Macro Domain
MetY
FABD-like
PPR
Helicase
RdRp
1
201
401
601
801
1001
1201
1401
1601
Positions

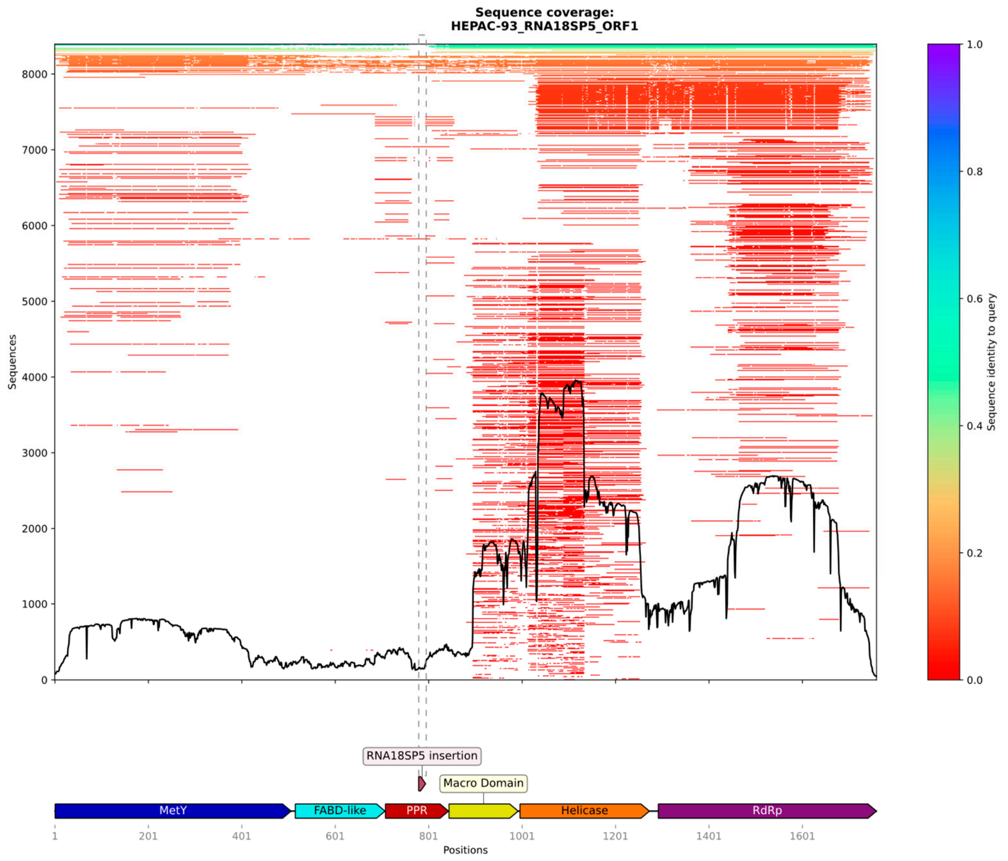
Sequence coverage:
HEPAC-93_RNA18SP5_ORF1
Sequences
0
1000
2000
3000
4000
5000
6000
7000
8000
Sequence identity to query
0.0
0.2
0.4
0.6
0.8
1.0
RNA18SP5 insertion
Macro Domain
MetY
FABD-like
PPR
Helicase
RdRp
1
201
401
601
801
1001
1201
1401
1601
Positions

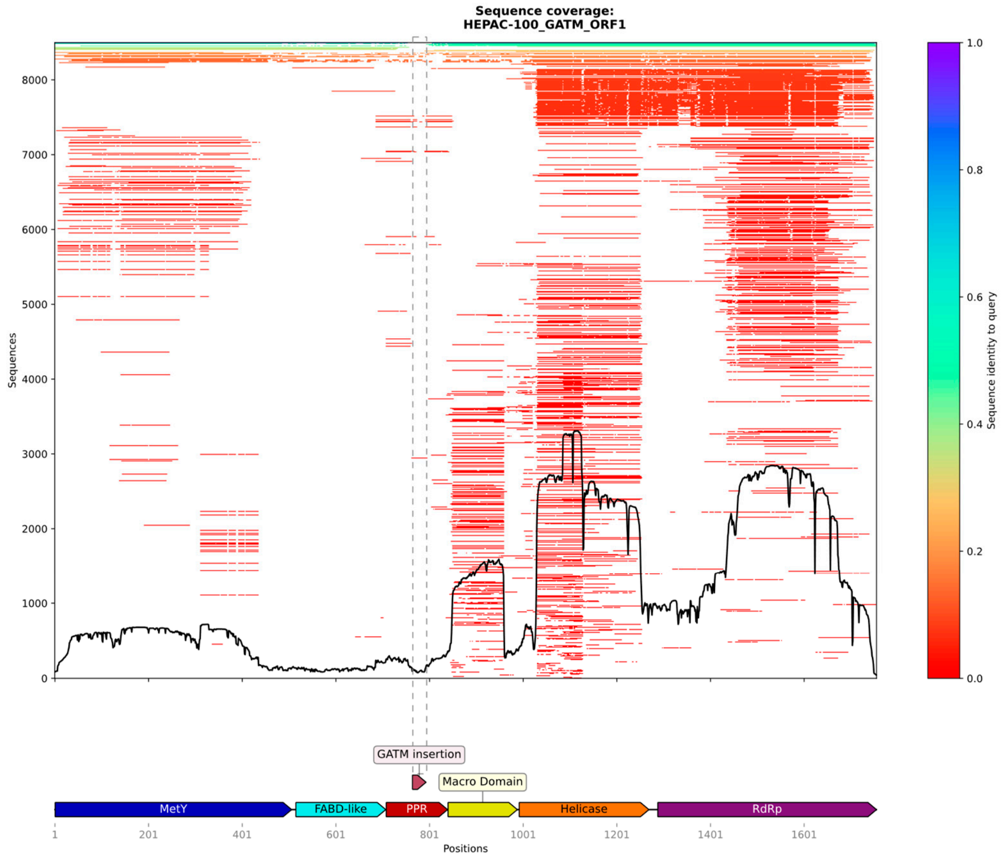
Sequence coverage:
HEPAC-100_GATM_ORF1
Sequences
0
1000
2000
3000
4000
5000
6000
7000
8000
Sequence identity to query
0.0
0.2
0.4
0.6
0.8
1.0
GATM insertion
Macro Domain
MetY
FABD-like
PPR
Helicase
RdRp
1
201
401
601
801
1001
1201
1401
1601
Positions

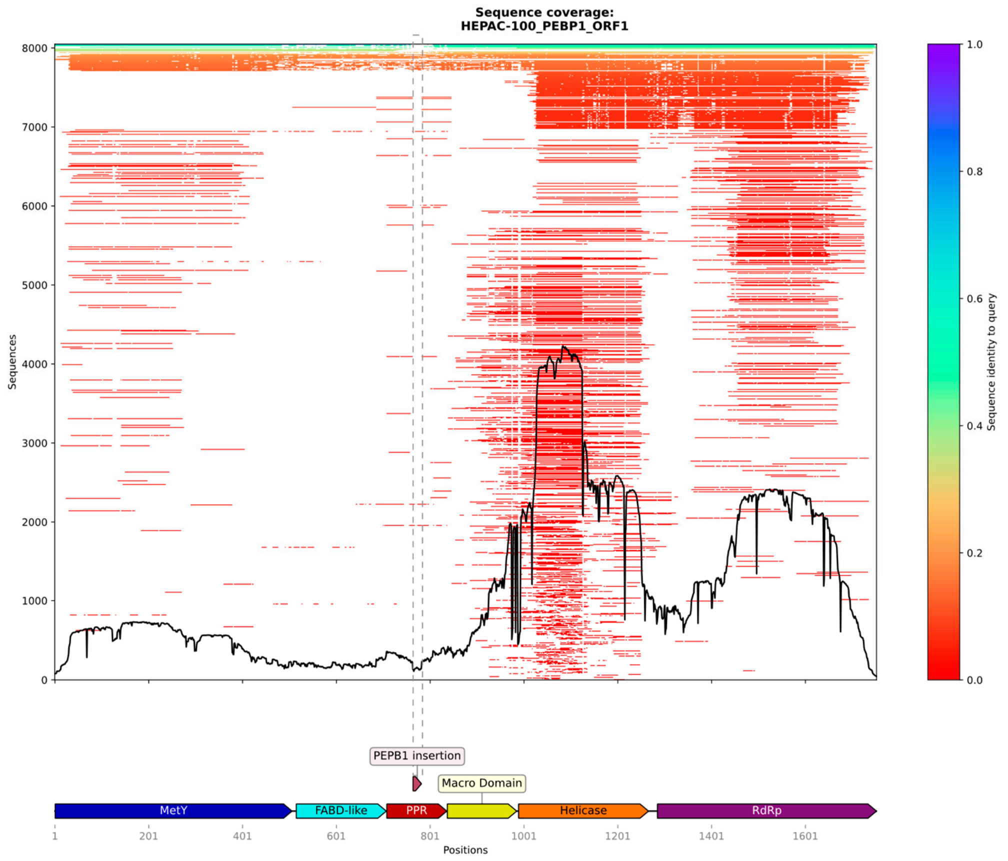
Sequence coverage:
HEPAC-100_PEBP1_ORF1
Sequences
Sequence identity to query
PEPB1 insertion
Macro Domain
MetY
FABD-like
PPR
Helicase
RdRp
Positions

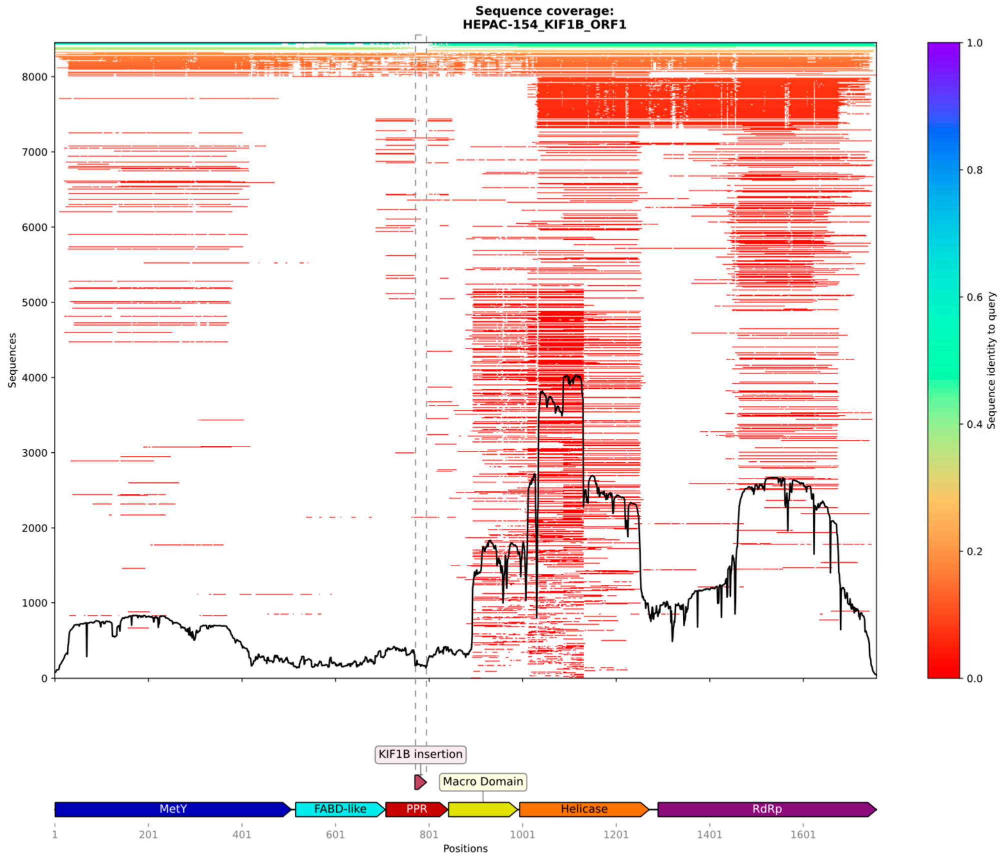
Sequence coverage:
HEPAC-154_KIF1B_ORF1
Sequences
Sequence identity to query
KIF1B insertion
Macro Domain
MetY
FABD-like
PPR
Helicase
RdRp
Positions

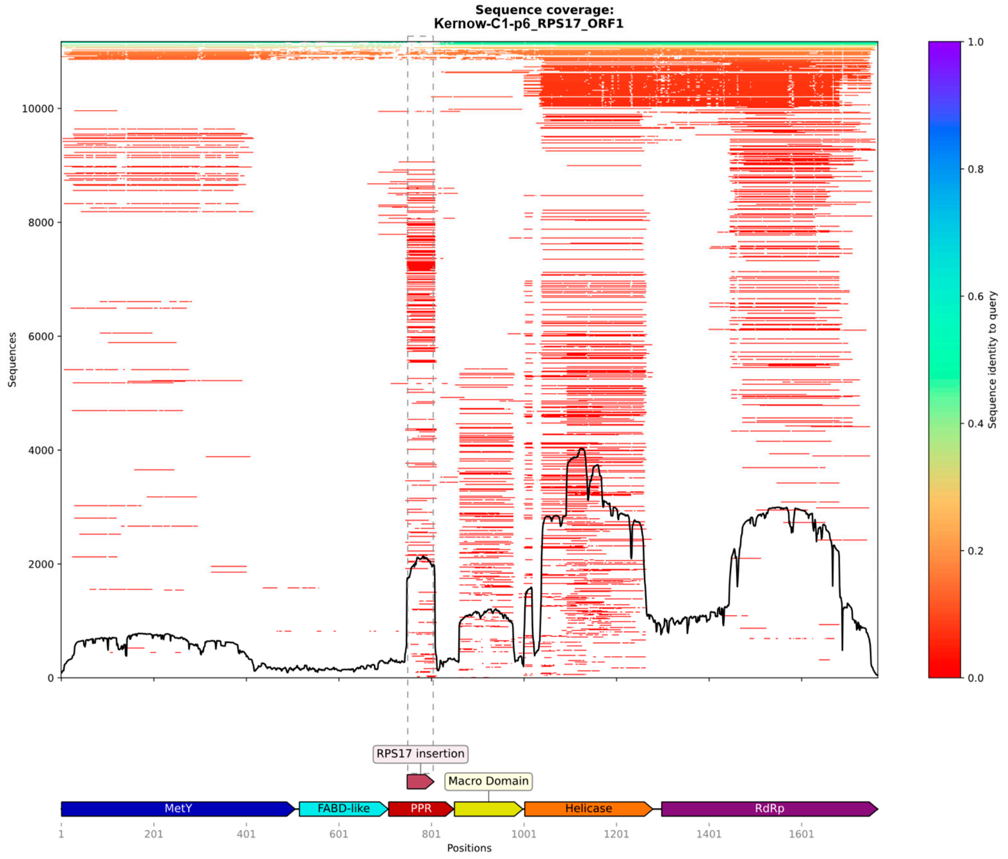

Sequence coverage:
Kernow-C1-p6_RPS17_ORF1
Sequences
10000
8000
6000
4000
2000
0
1.0
0.8
0.6
0.4
0.2
0.0
Sequence identity to query
RPS17 insertion
Macro Domain
MetY
FABD-like
PPR
Helicase
RdRp
1
201
401
601
801
1001
1201
1401
1601
Positions

Supplementary Figure S2. The Alphafold2 pORF1 pLDDT scores plots for the 16 WT strains and the 9 strains with insertions.

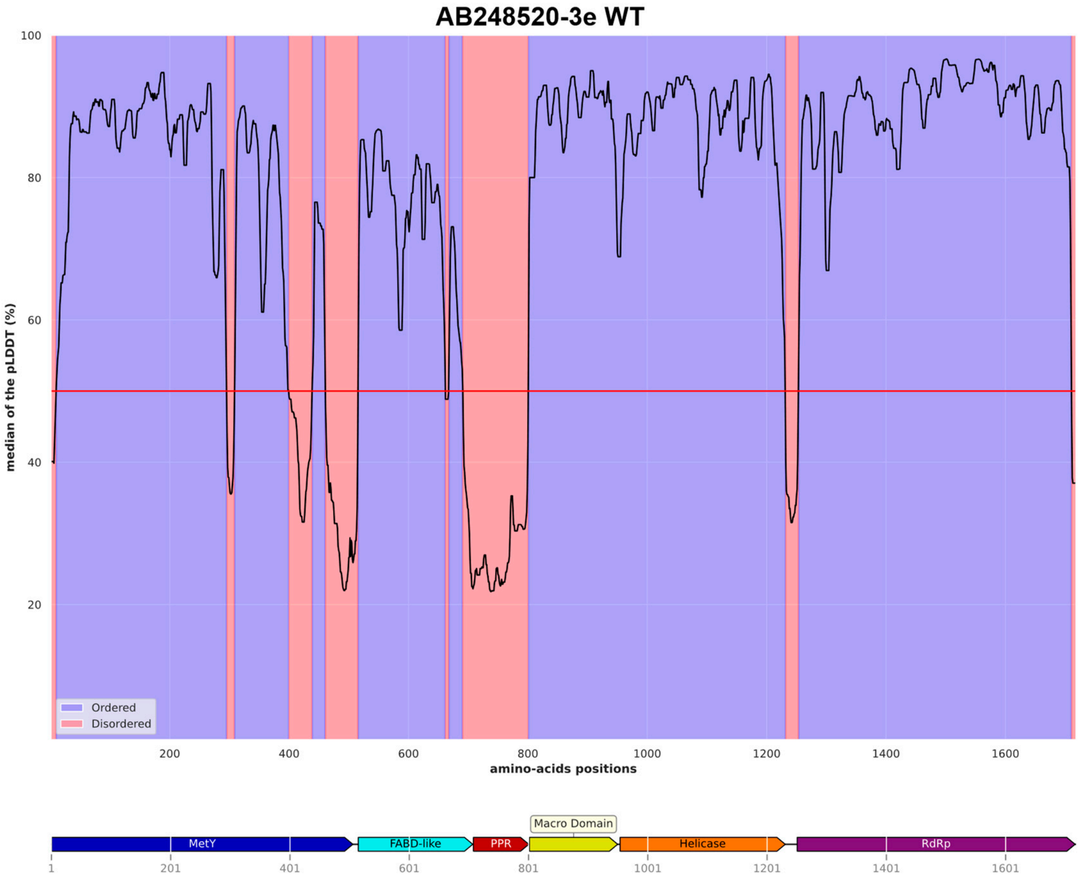

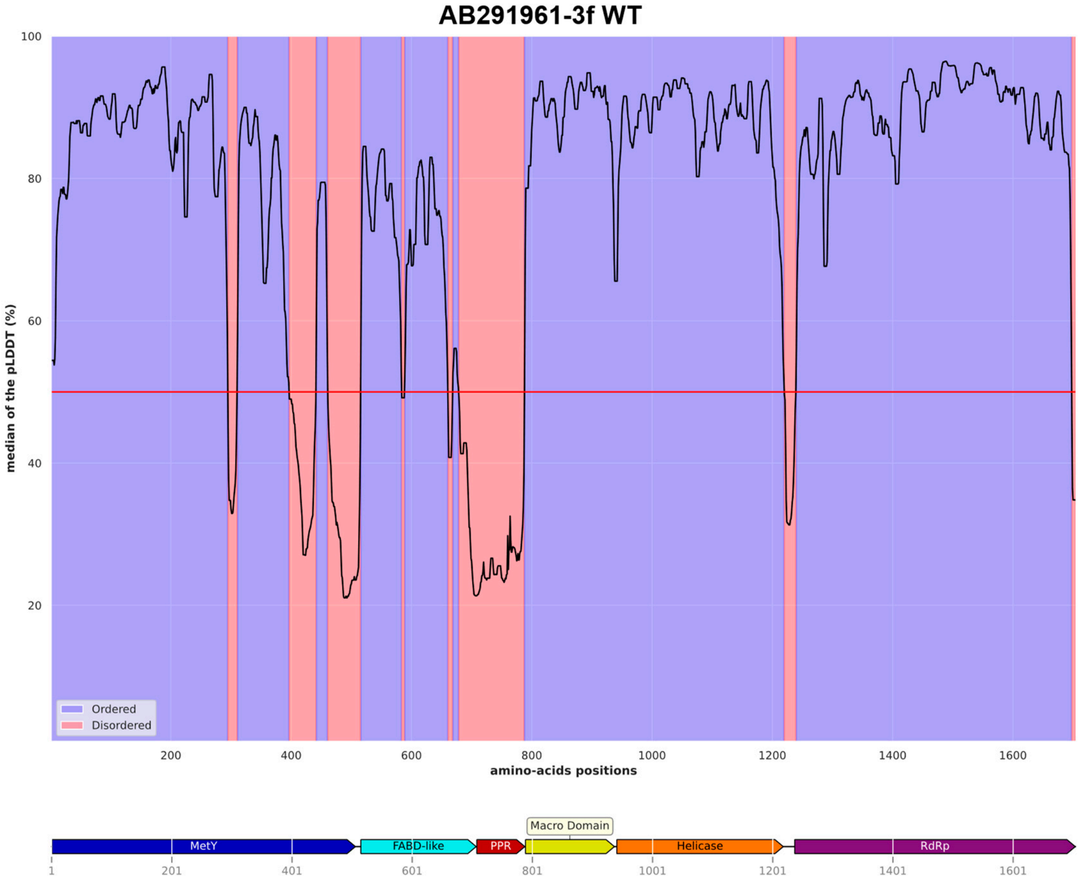

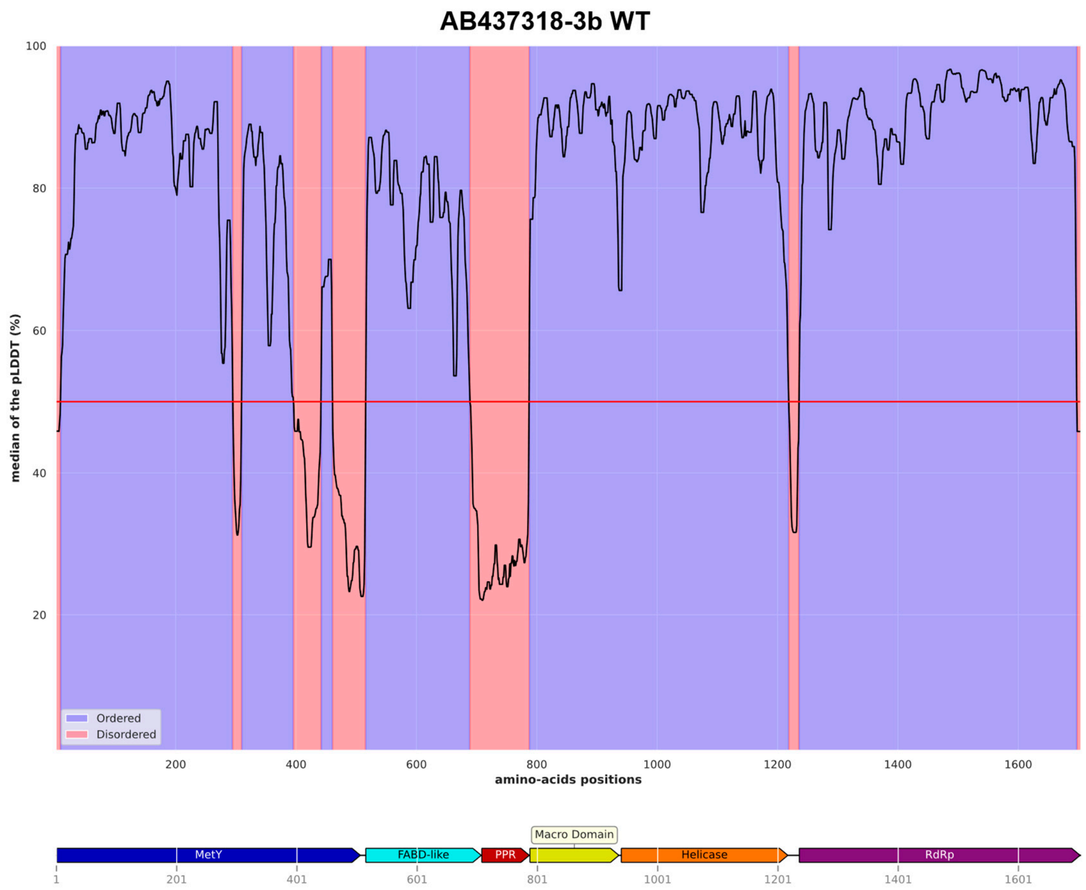
AB437318-3b WT
median of the pLDDT (%)
amino-acids positions
Ordered
Disordered
Macro Domain
MetY
FABD-like
PPR
Helicase
RdRp

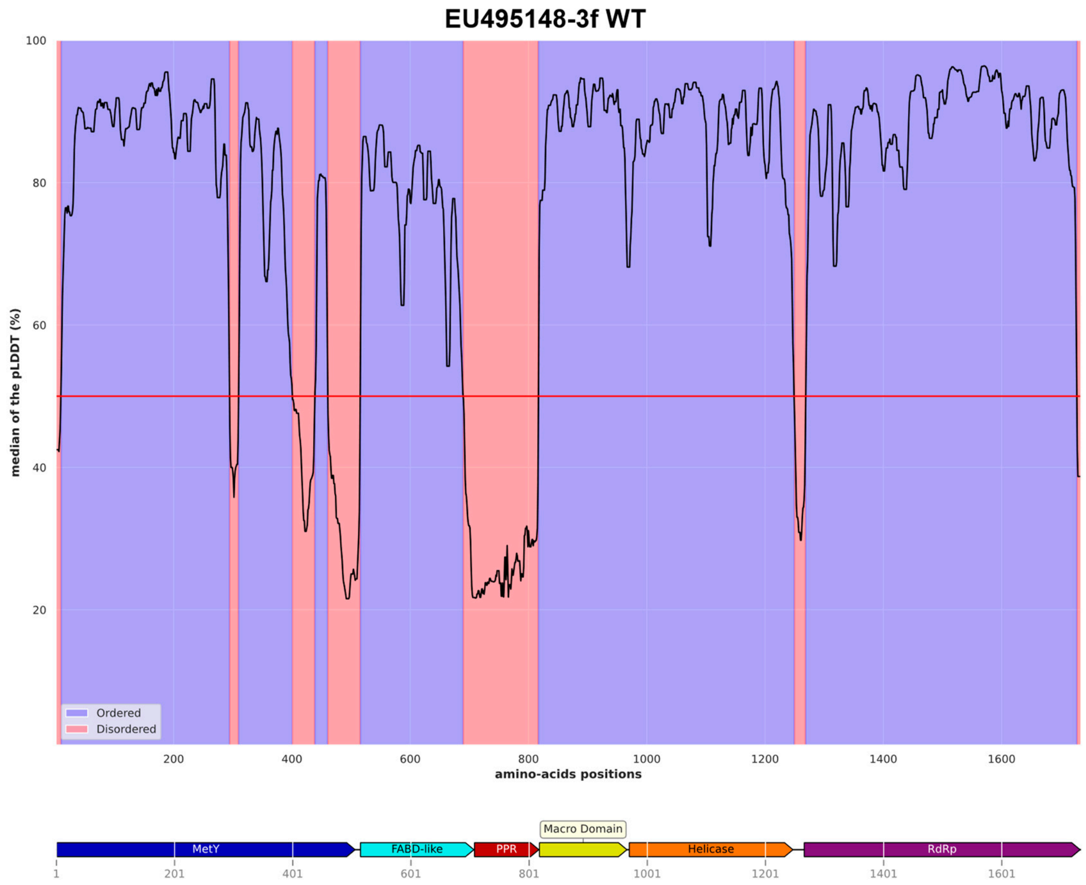
EU495148-3f WT
median of the pLDDT (%)
amino-acids positions
Ordered
Disordered
Macro Domain
MetY
FABD-like
PPR
Helicase
RdRp

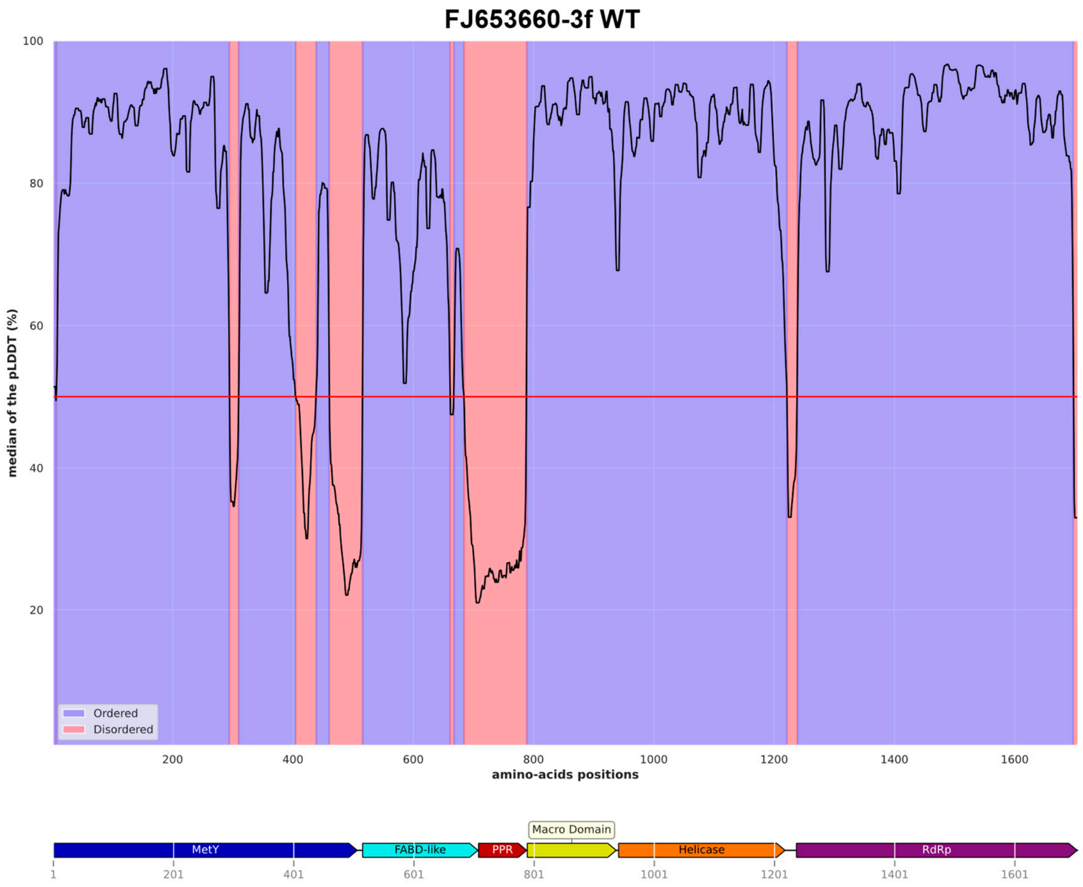
FJ653660-3f WT
median of the pLDDT (%)
amino-acids positions
Ordered
Disordered
MetY
FABD-like
PPR
Macro Domain
Helicase
RdRp

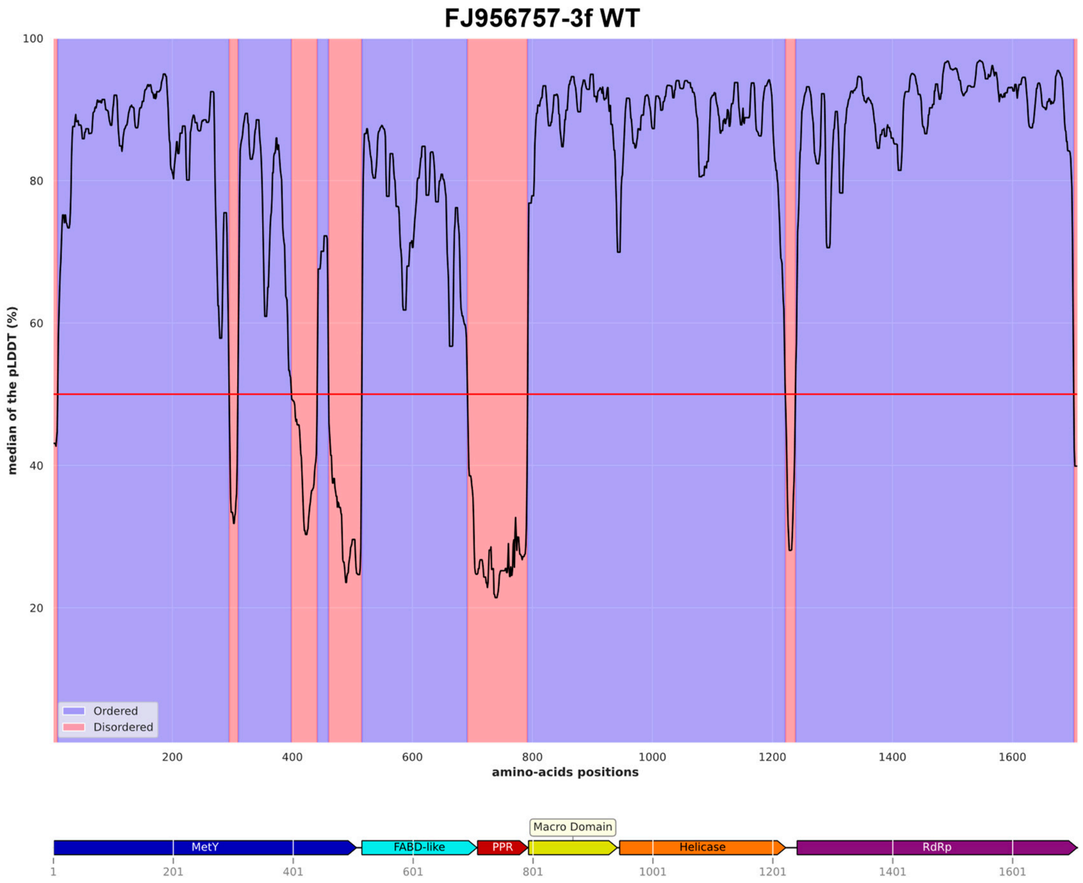
FJ956757-3f WT
median of the pLDDT (%)
amino-acids positions
Ordered
Disordered
MetY
FABD-like
PPR
Macro Domain
Helicase
RdRp

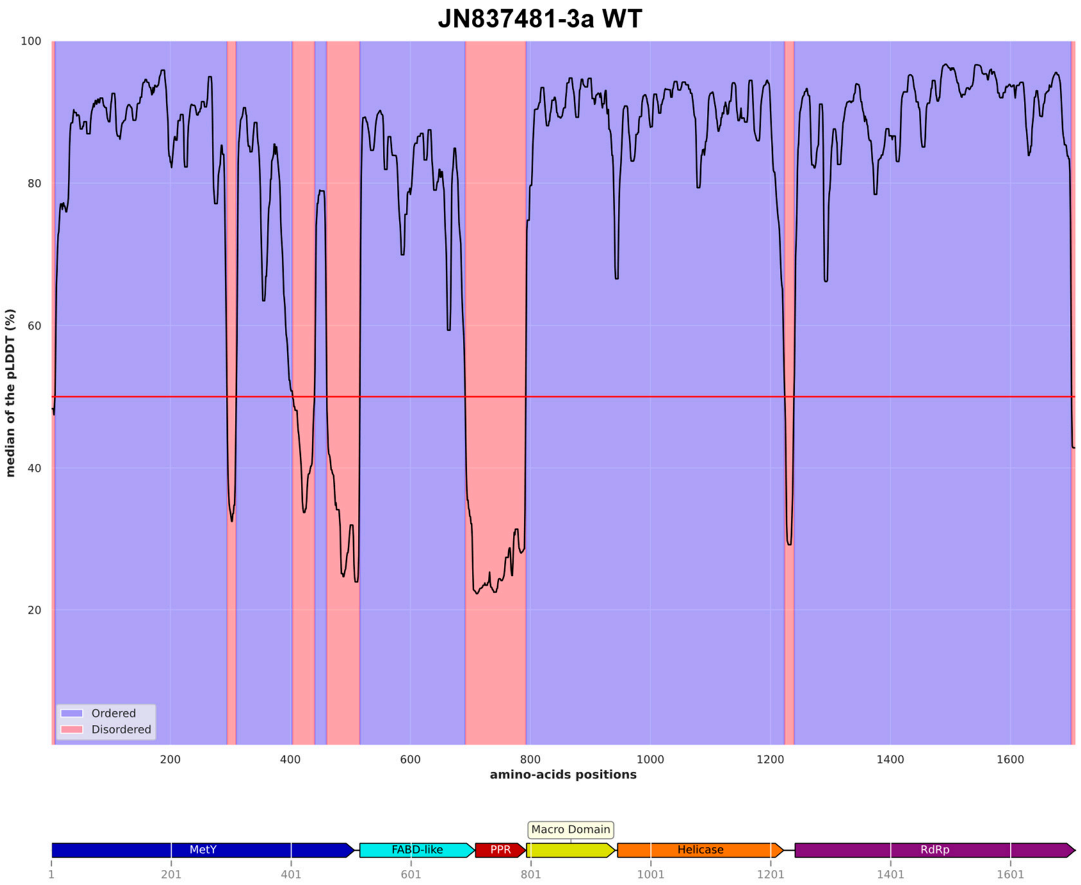
JN837481-3a WT
median of the pLDDT (%)
amino-acids positions
Ordered
Disordered
Macro Domain
MetY
FABD-like
PPR
Helicase
RdRp

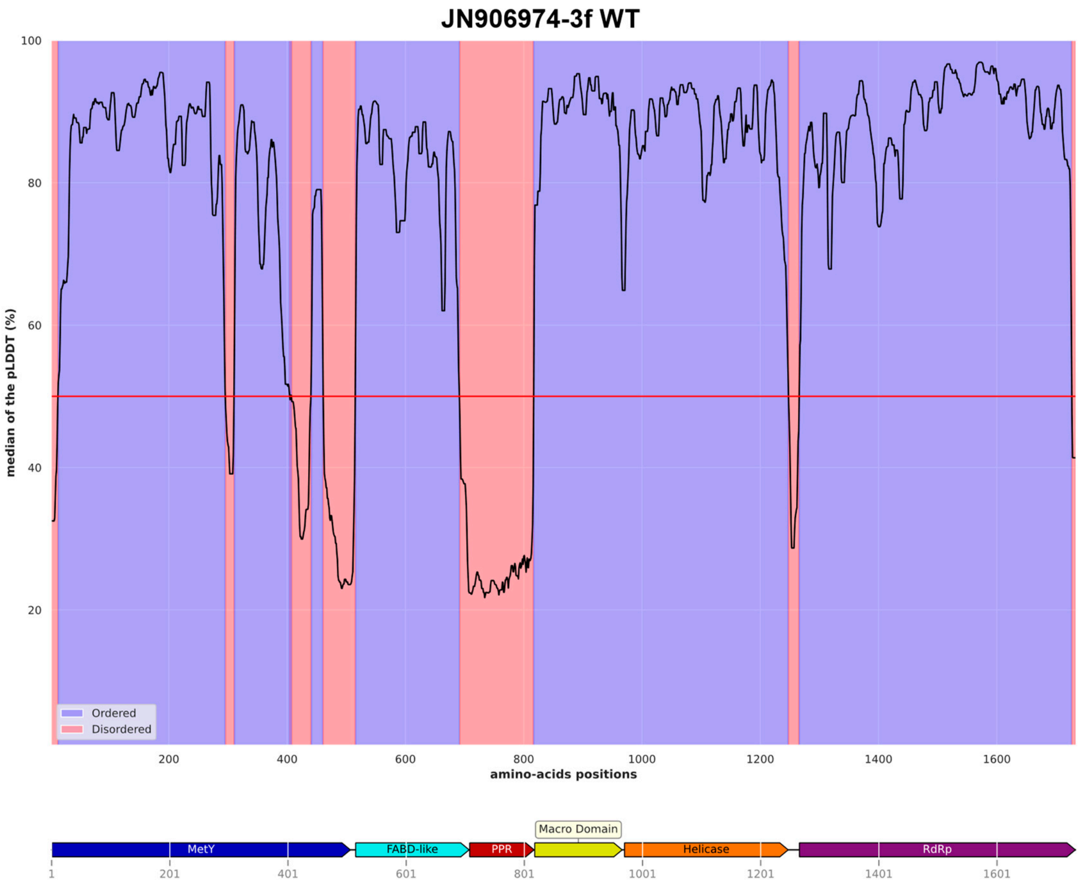
JN906974-3f WT
median of the pLDDT (%)
amino-acids positions
Ordered
Disordered
Macro Domain
MetY
FABD-like
PPR
Helicase
RdRp

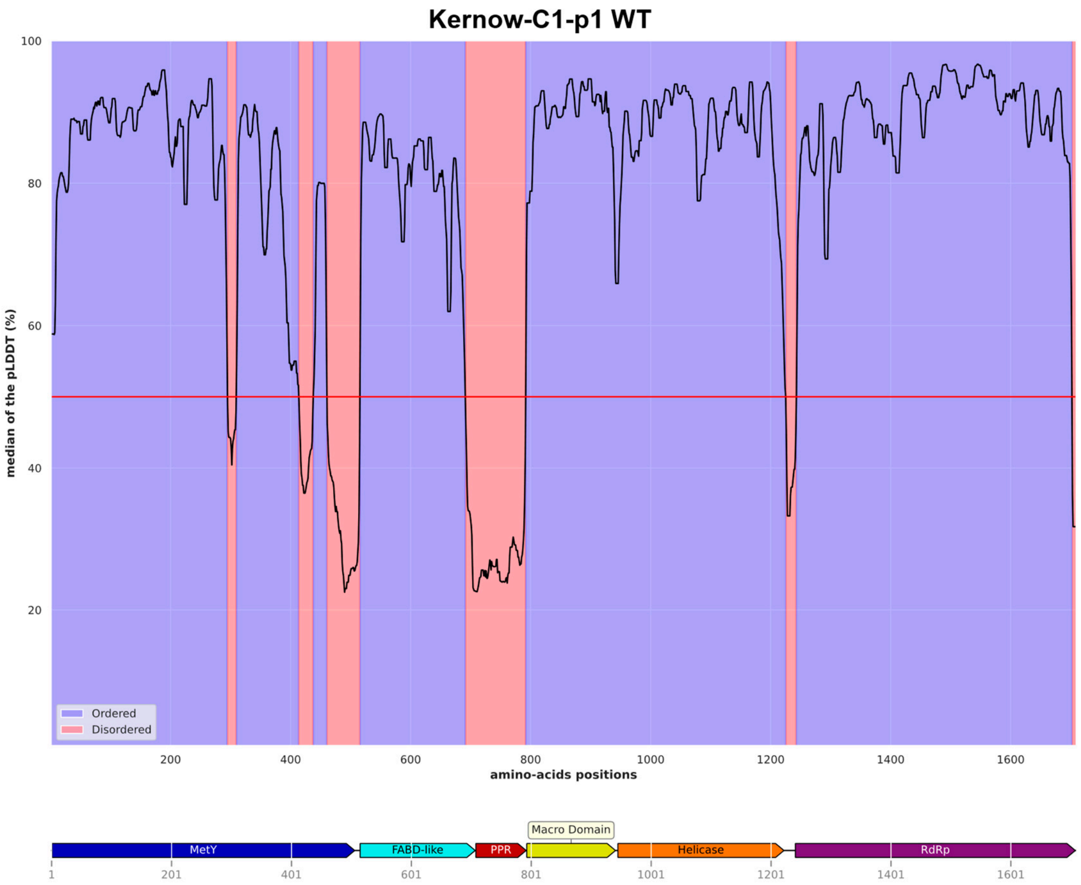
Kernow-C1-p1 WT
median of the pLDDT (%)
amino-acids positions
Ordered
Disordered
Macro Domain
MetY
FABD-like
PPR
Helicase
RdRp

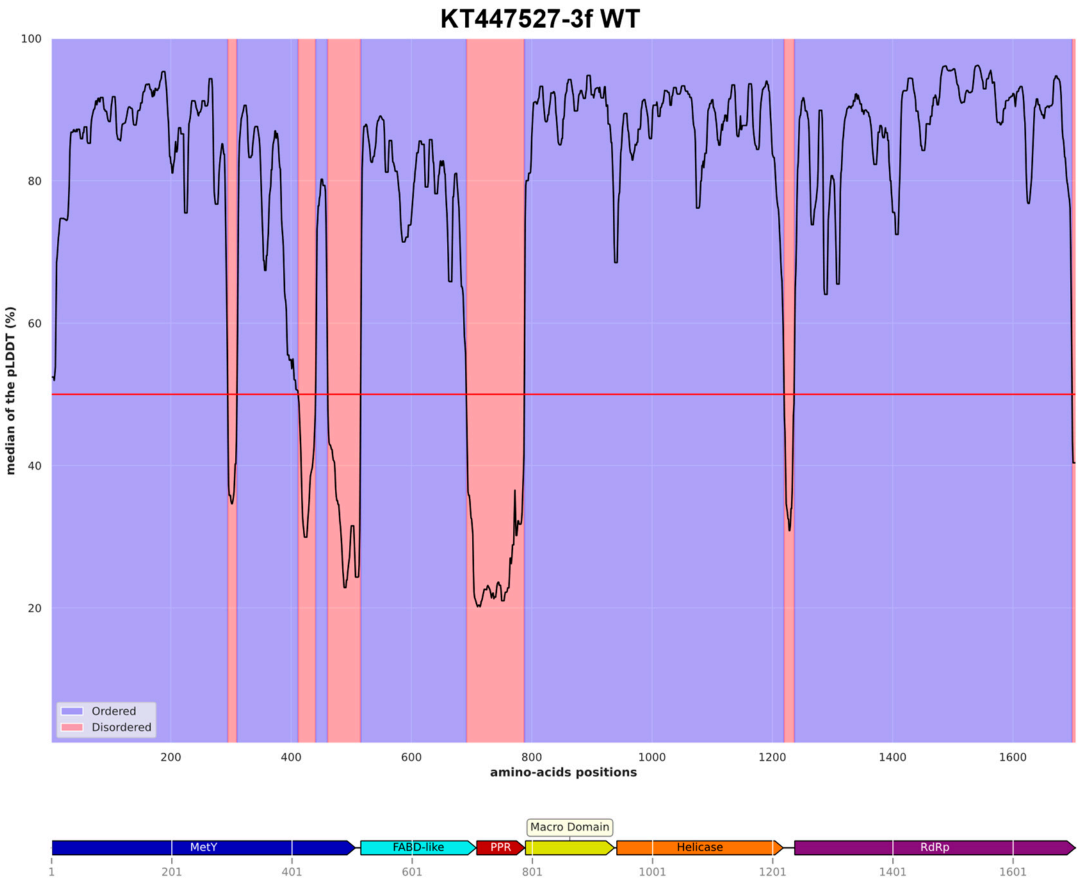
KT447527-3f WT
median of the pLDDT (%)
amino-acids positions
Ordered
Disordered
Macro Domain
MetY
FABD-like
PPR
Helicase
RdRp

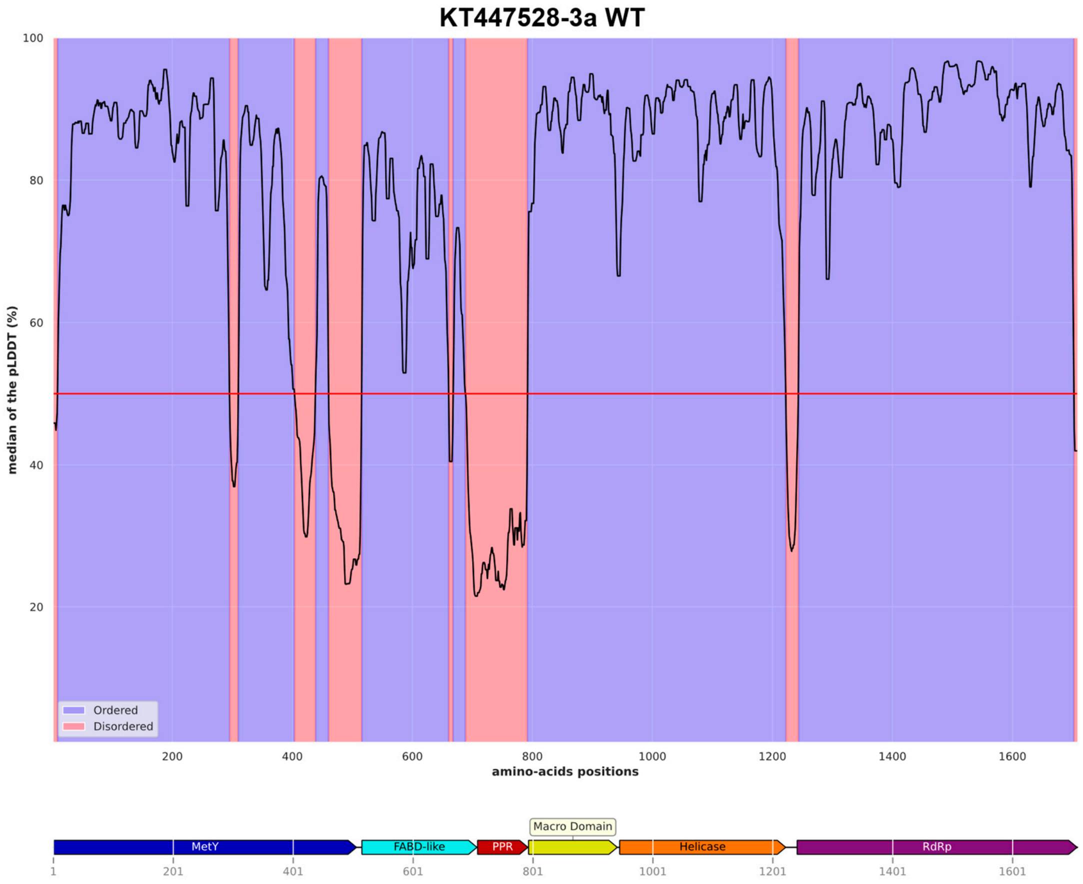
KT447528-3a WT
median of the pLDDT (%)
100
80
60
40
20
Ordered
Disordered
200
400
600
800
1000
1200
1400
1600
amino-acids positions
Macro Domain
MetY
FABD-like
PPR
Helicase
RdRp
1
201
401
601
801
1001
1201
1401
1601

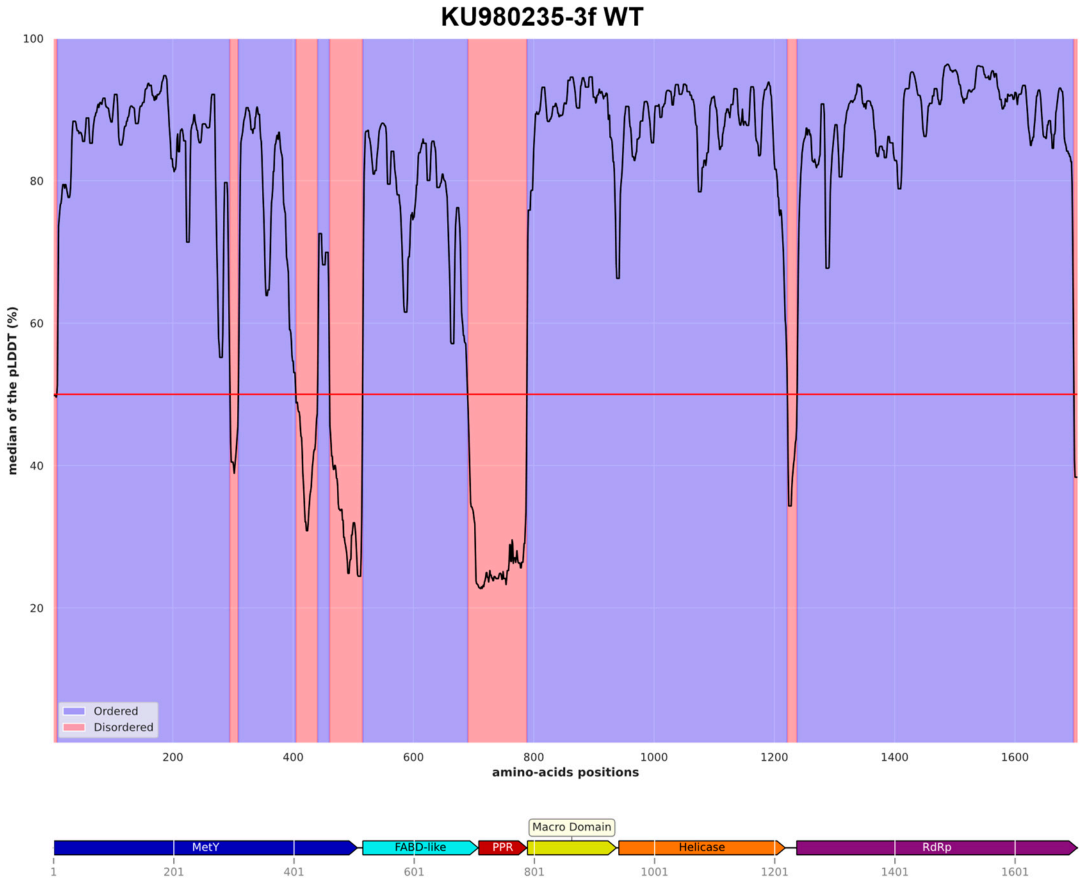
KU980235-3f WT
median of the pLDDT (%)
100
80
60
40
20
Ordered
Disordered
200
400
600
800
1000
1200
1400
1600
amino-acids positions
Macro Domain
MetY
FABD-like
PPR
Helicase
RdRp
1
201
401
601
801
1001
1201
1401
1601

KY232312-3f WT

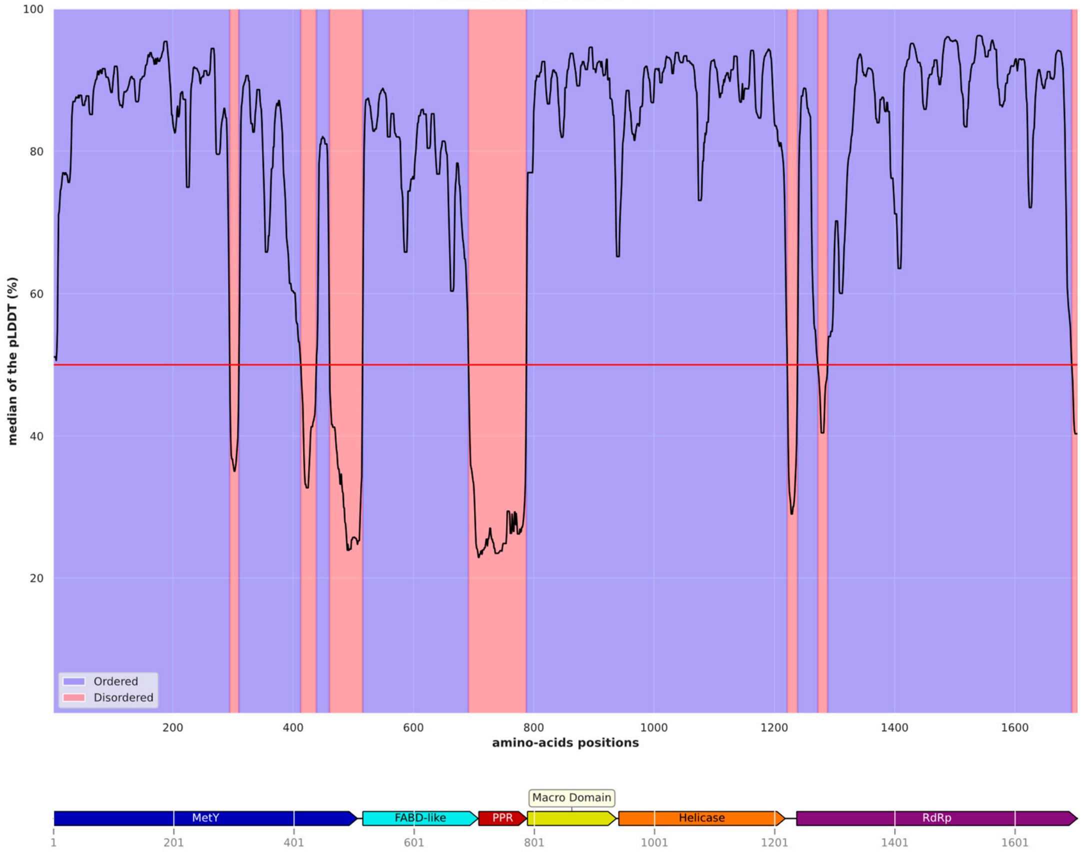

KY780957-3h WT

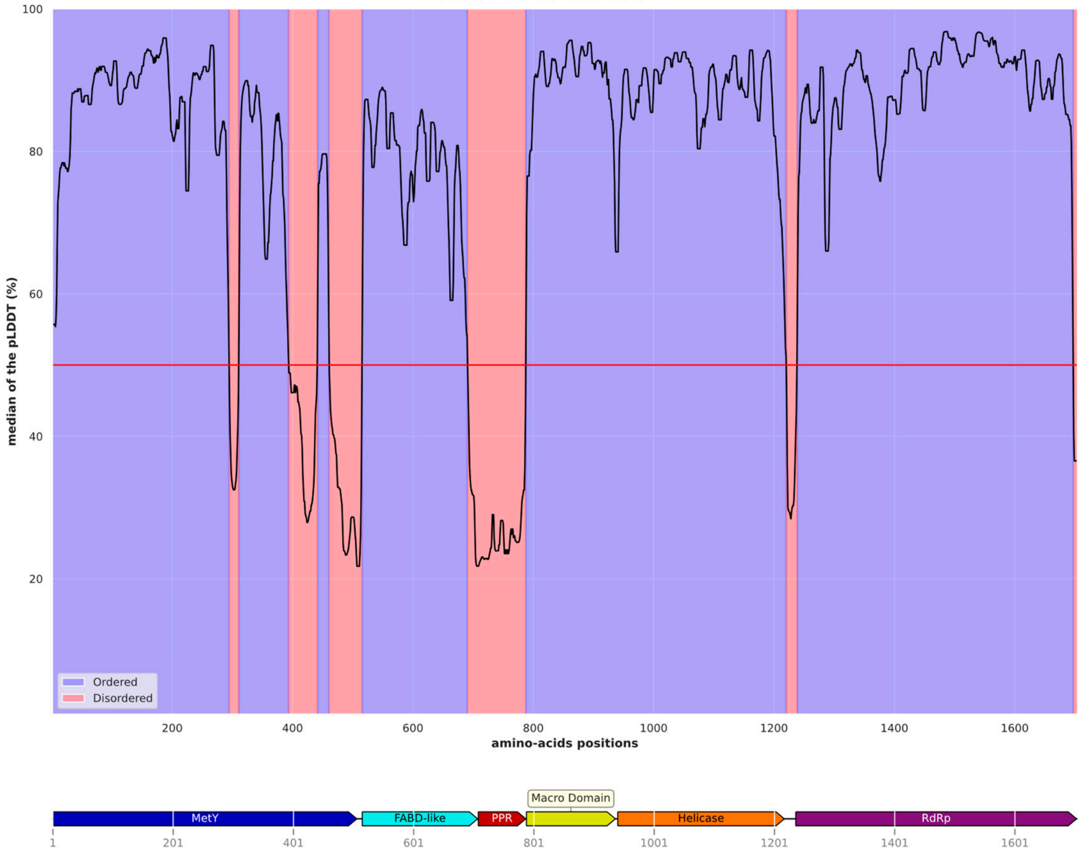

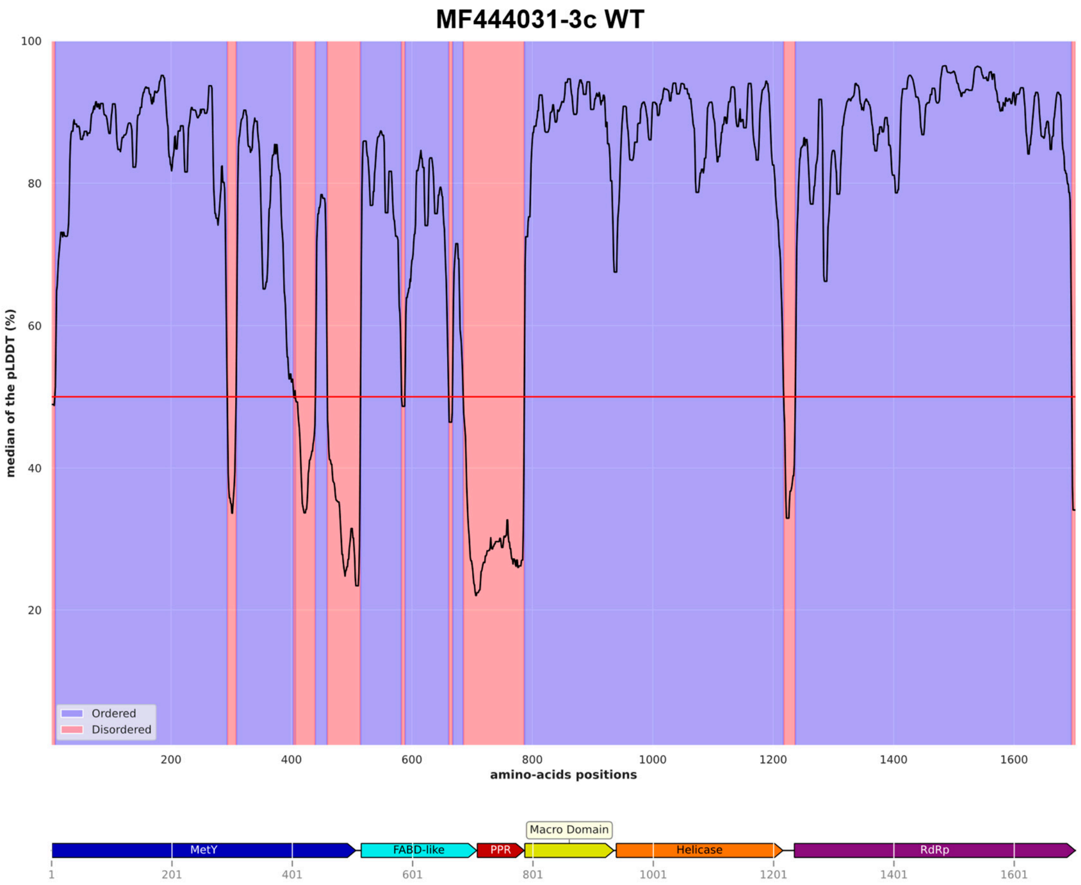
MF444031-3c WT
median of the pLDDT (%)
amino-acids positions
Ordered
Disordered
MetY
FABD-like
PPR
Macro Domain
Helicase
RdRp

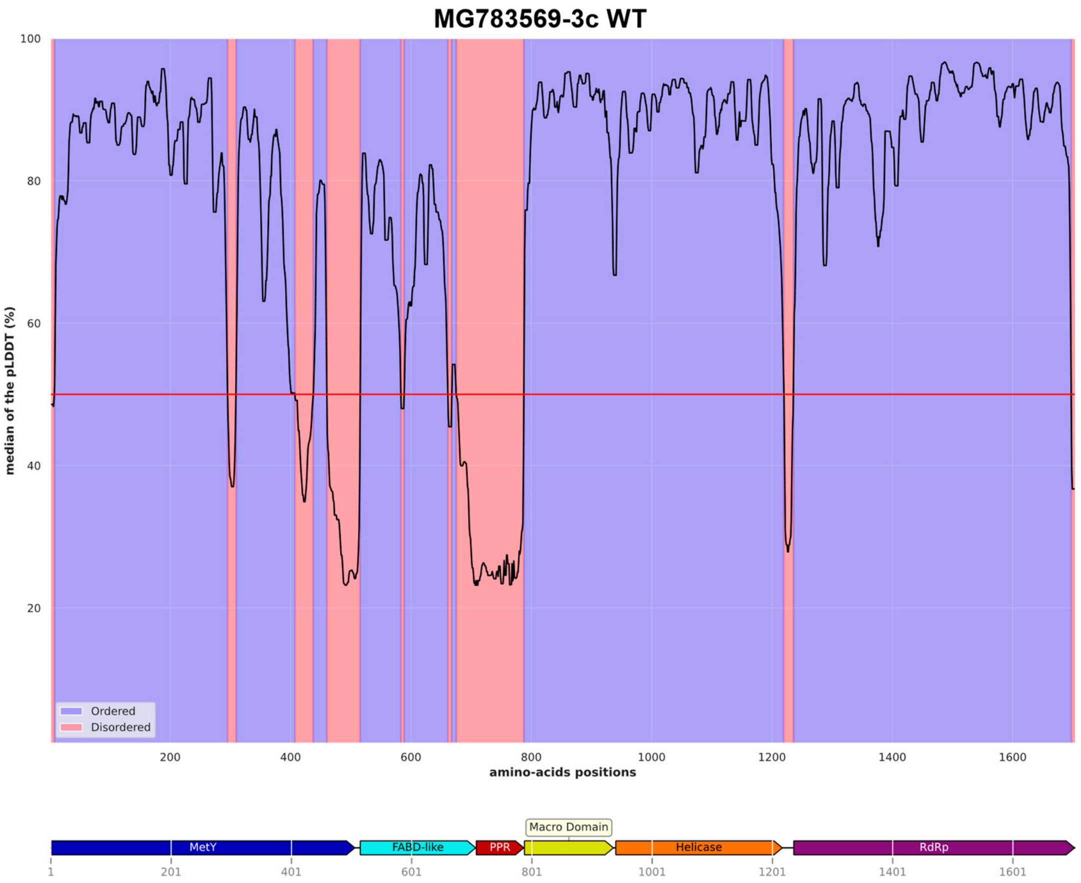
MG783569-3c WT
median of the pLDDT (%)
amino-acids positions
Ordered
Disordered
MetY
FABD-like
PPR
Macro Domain
Helicase
RdRp

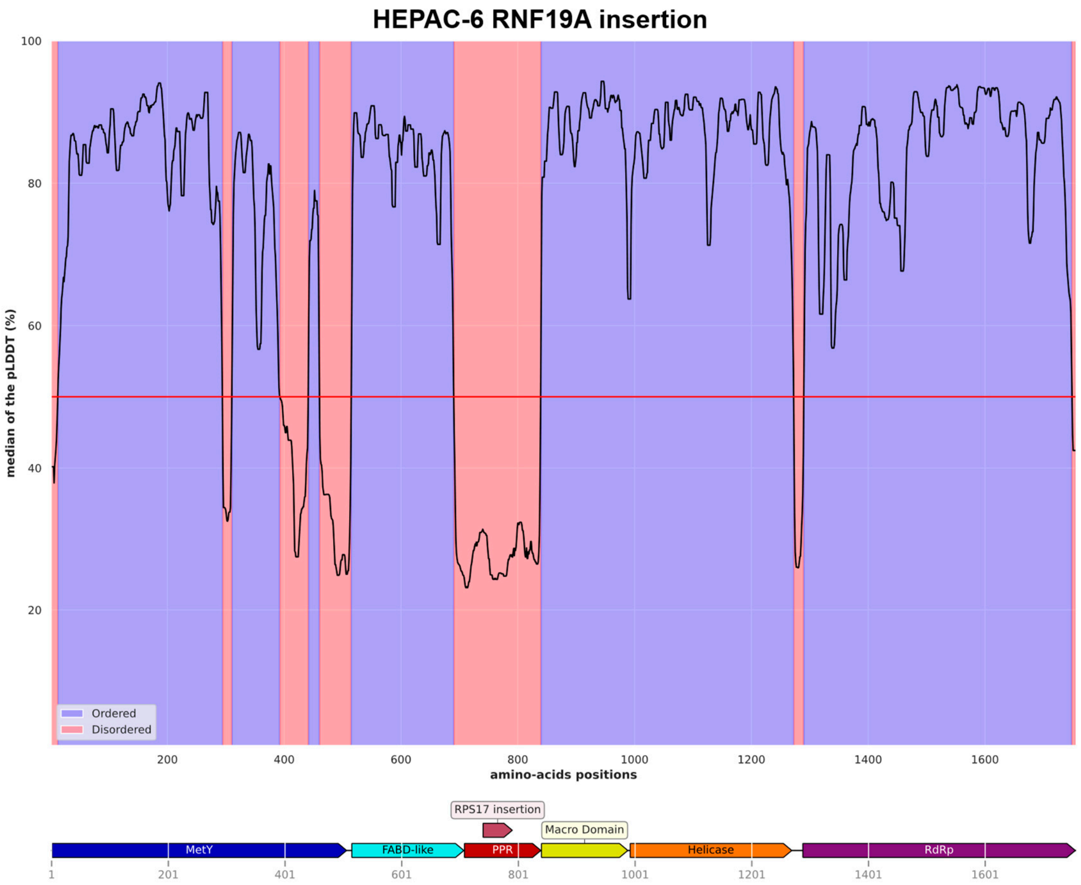
HEPAC-6 RNF19A insertion
median of the pLDDT (%)
Ordered
Disordered
amino-acids positions
RPS17 insertion
Macro Domain
MetY
FABD-like
PPR
Helicase
RdRp

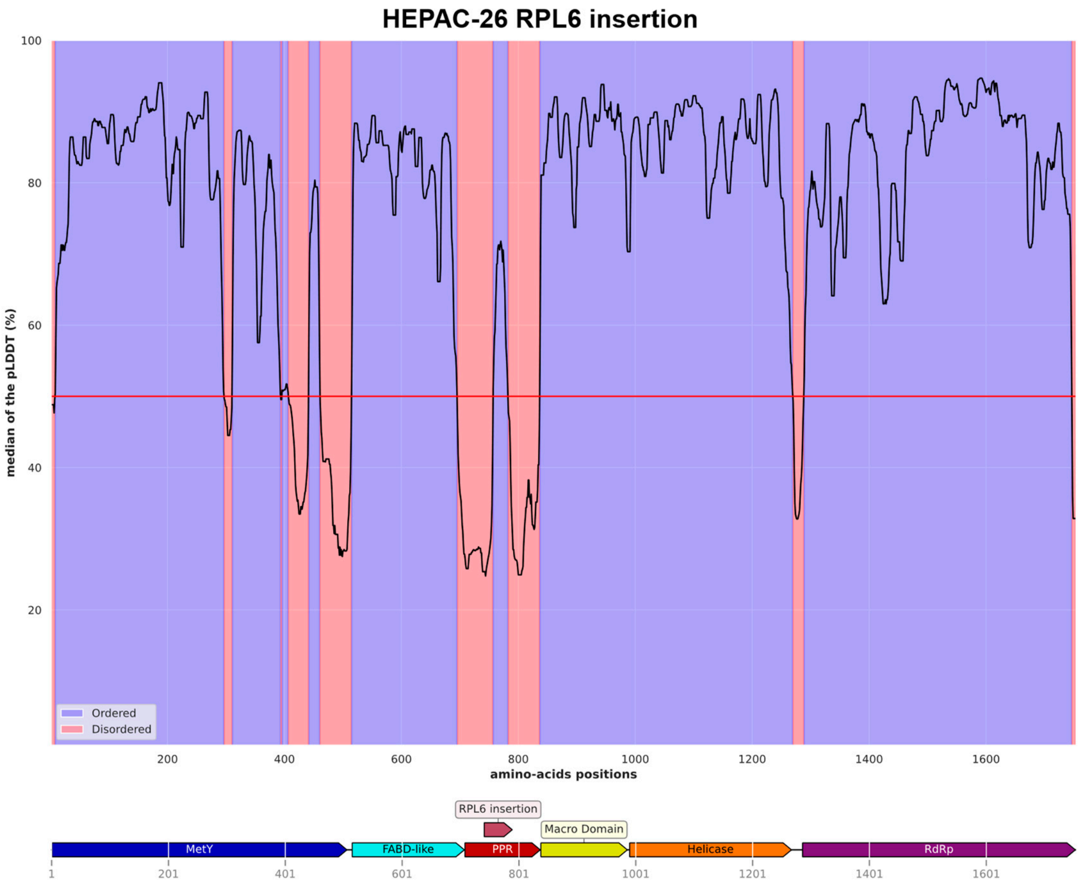
HEPAC-26 RPL6 insertion
median of the pLDDT (%)
Ordered
Disordered
amino-acids positions
RPL6 insertion
Macro Domain
MetY
FABD-like
PPR
Helicase
RdRp

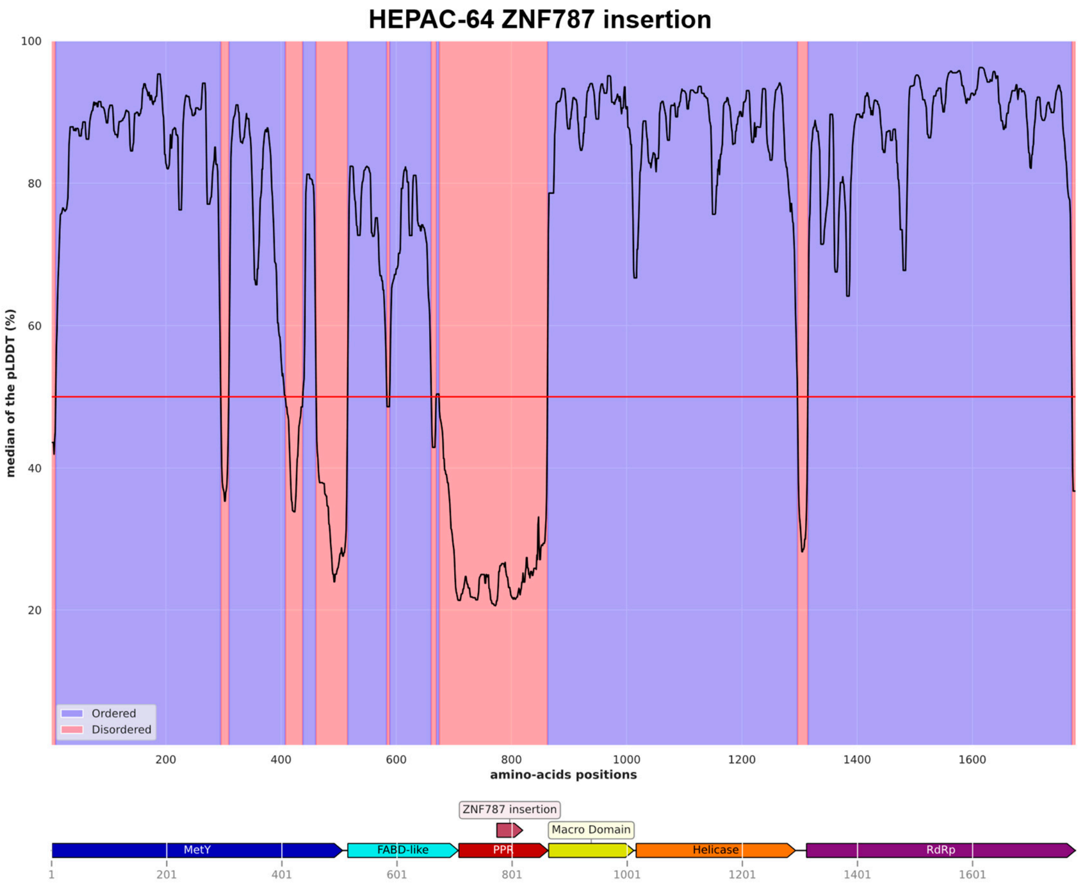
HEPAC-64 ZNF787 insertion
median of the pLDDT (%)
amino-acids positions
Ordered
Disordered
ZNF787 insertion
Macro Domain
MetY
FABD-like
PPR
Helicase
RdRp

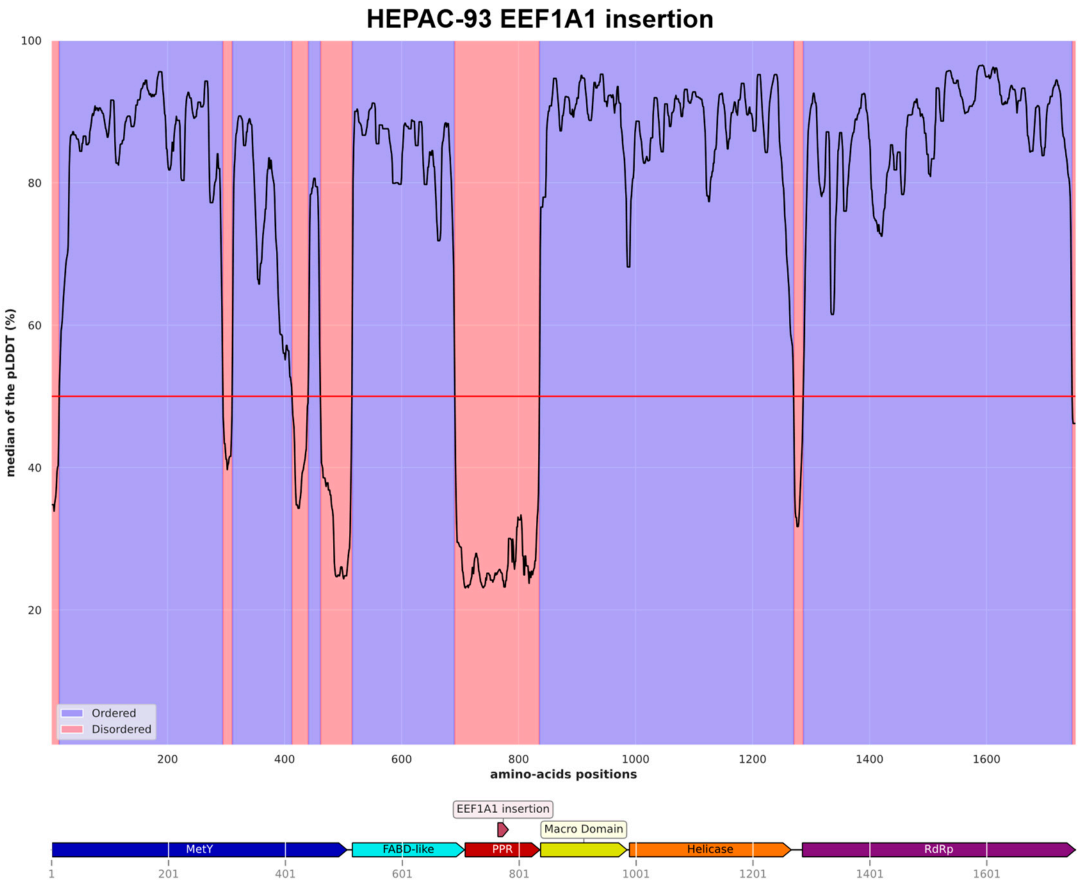
HEPAC-93 EEF1A1 insertion
median of the pLDDT (%)
amino-acids positions
Ordered
Disordered
EEF1A1 insertion
Macro Domain
MetY
FABD-like
PPR
Helicase
RdRp

HEPAC-93 RNA18SP5 insertion

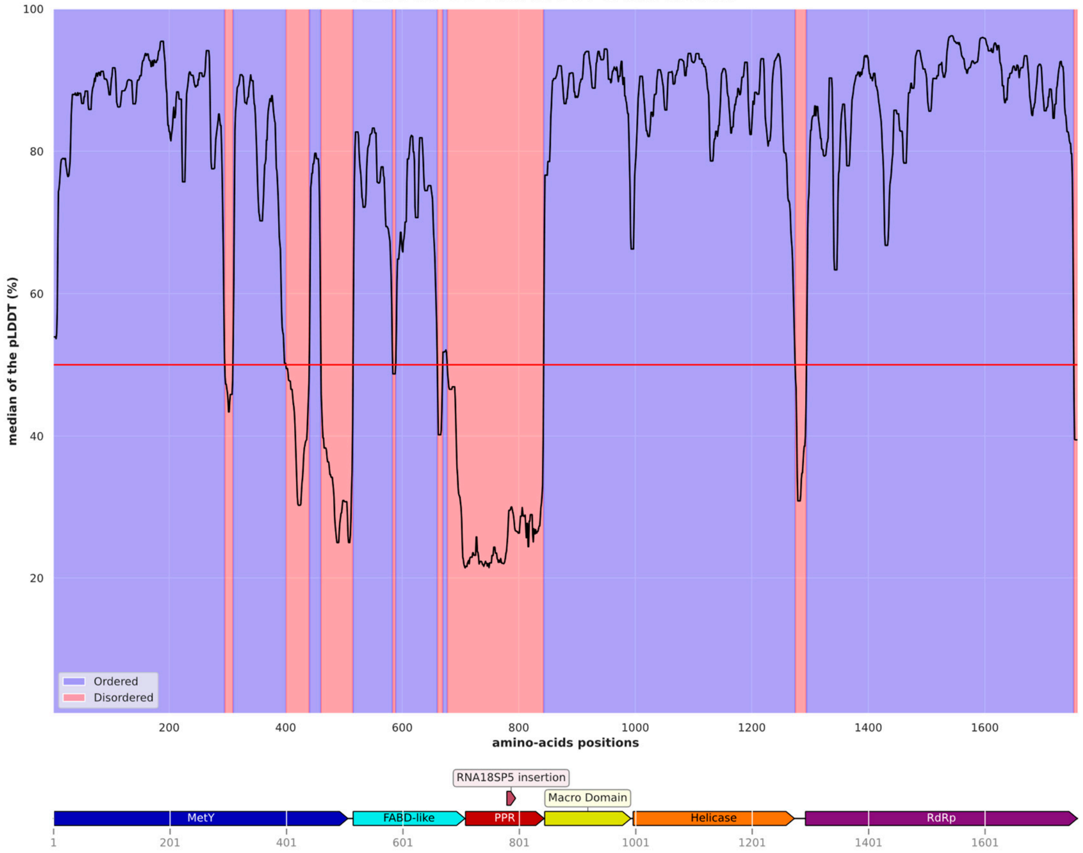

HEPAC-100 GATM insertion

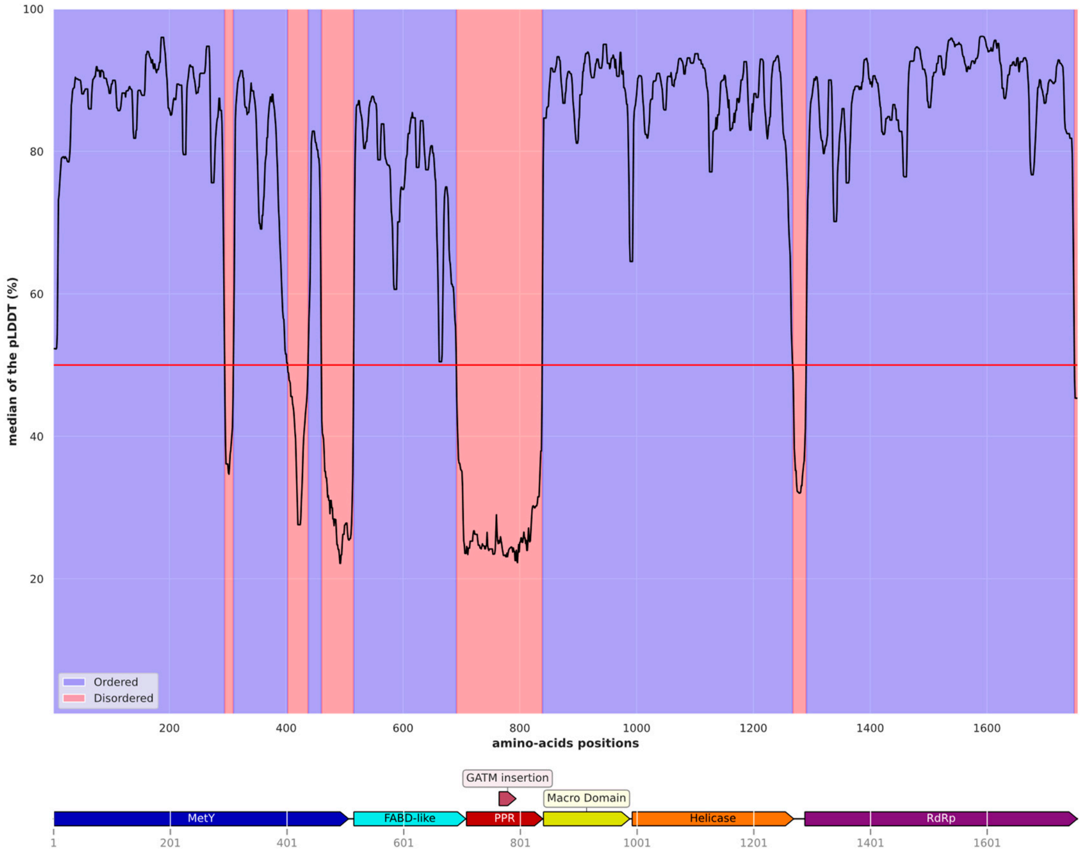

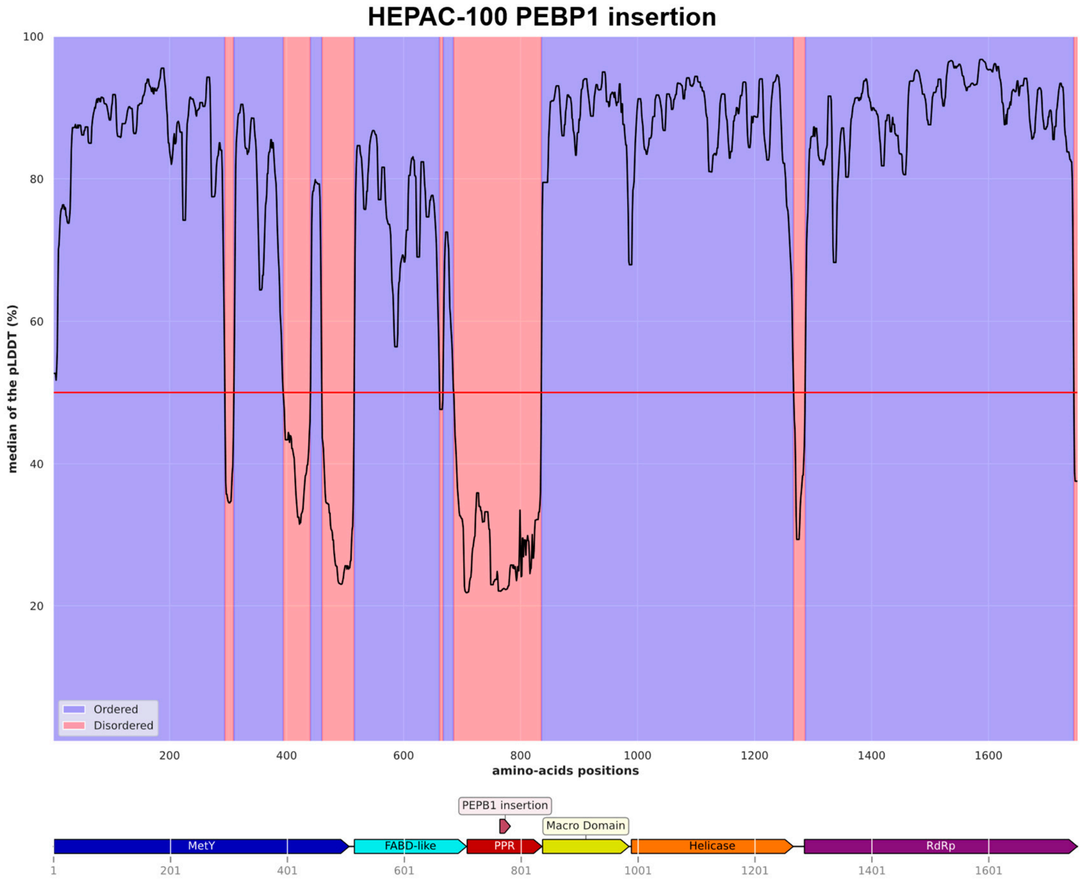
HEPAC-100 PEBP1 insertion
median of the pLDDT (%)
amino-acids positions
Ordered
Disordered
PEPB1 insertion
Macro Domain
MetY
FABD-like
PPR
Helicase
RdRp

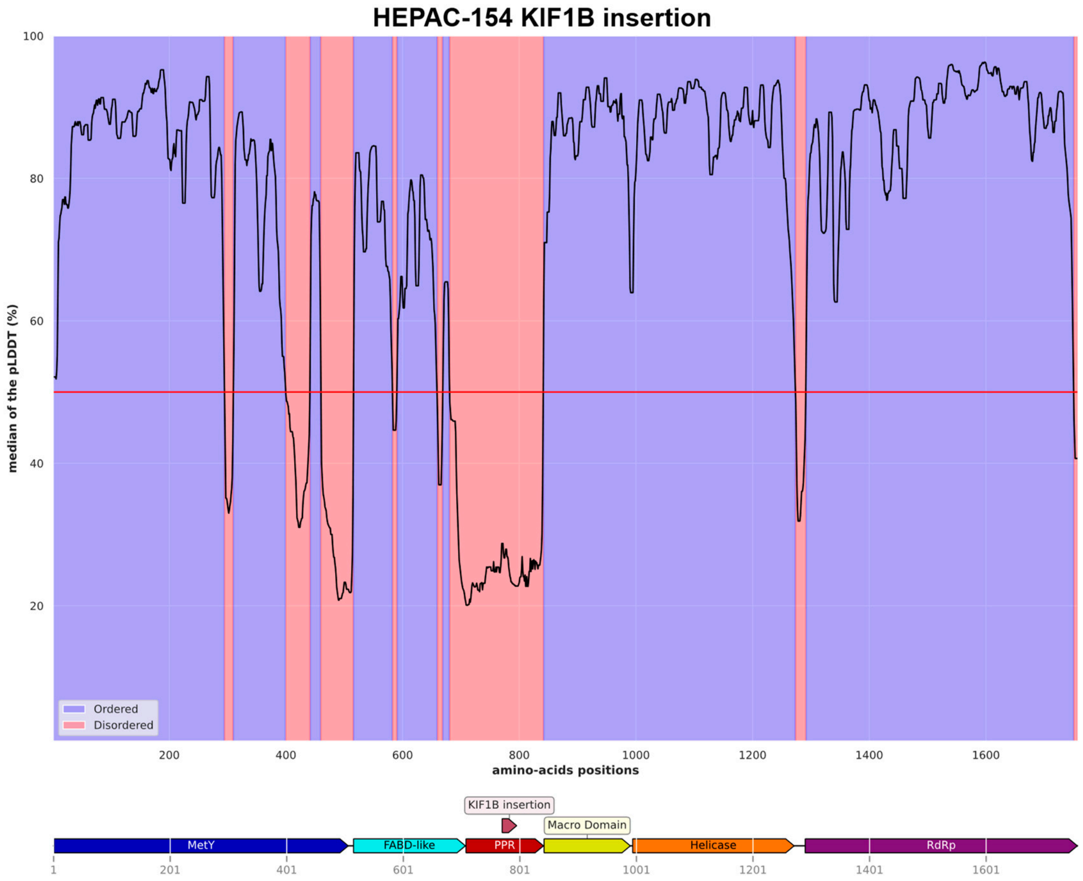
HEPAC-154 KIF1B insertion
median of the pLDDT (%)
amino-acids positions
Ordered
Disordered
KIF1B insertion
Macro Domain
MetY
FABD-like
PPR
Helicase
RdRp

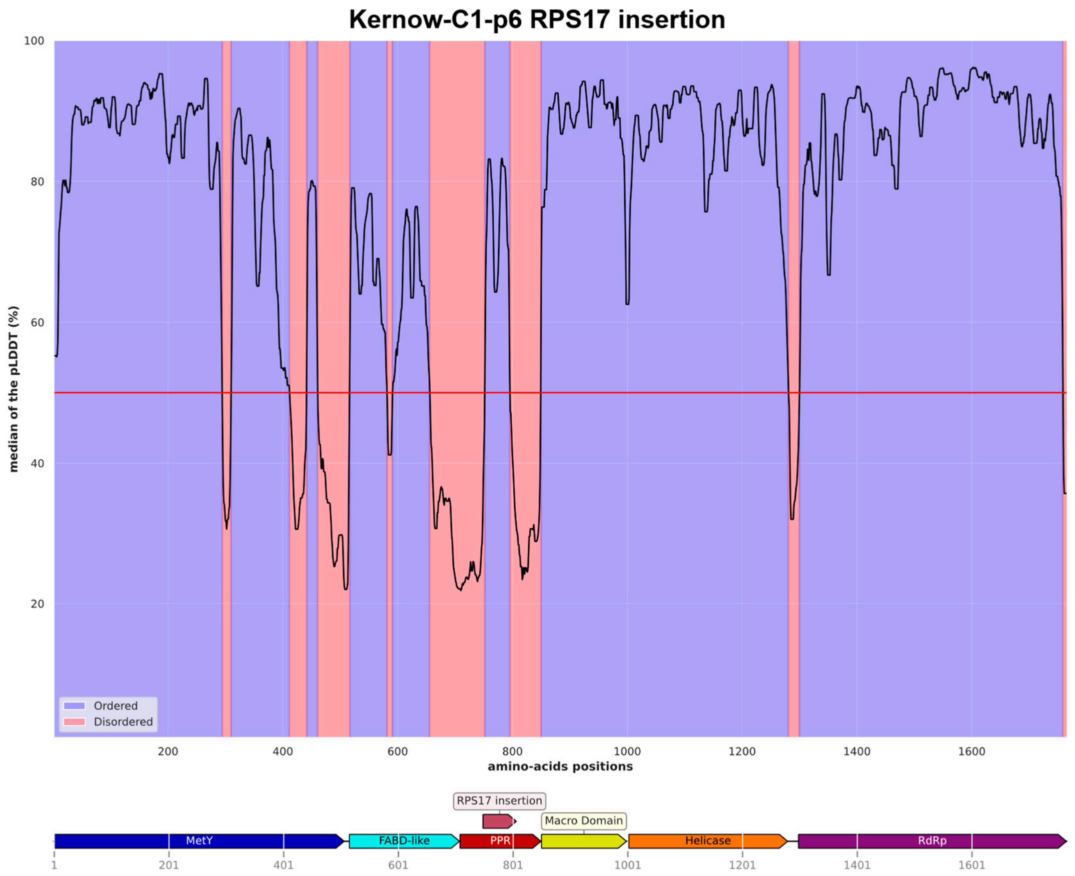
Kernow-C1-p6 RPS17 insertion
median of the pLDDT (%)
100
80
60
40
20
Ordered
Disordered
200
400
600
800
1000
1200
1400
1600
amino-acids positions
RPS17 insertion
Macro Domain
MetY
FABD-like
PPR
Helicase
RdRp
1
201
401
601
801
1001
1201
1401
1601

Supplementary Figure S3. The AlphaFold3 structure prediction of the Kernow-C1-p6 pORF1 with the *RPS17* insertion. The domains are colored: the MetY domain in blue, the FABD/MBD in green, the PPR in pink, the *RPS17* insertion in red, the Macro domain in cyan, the Helicase in orange and the RdRp in magenta.

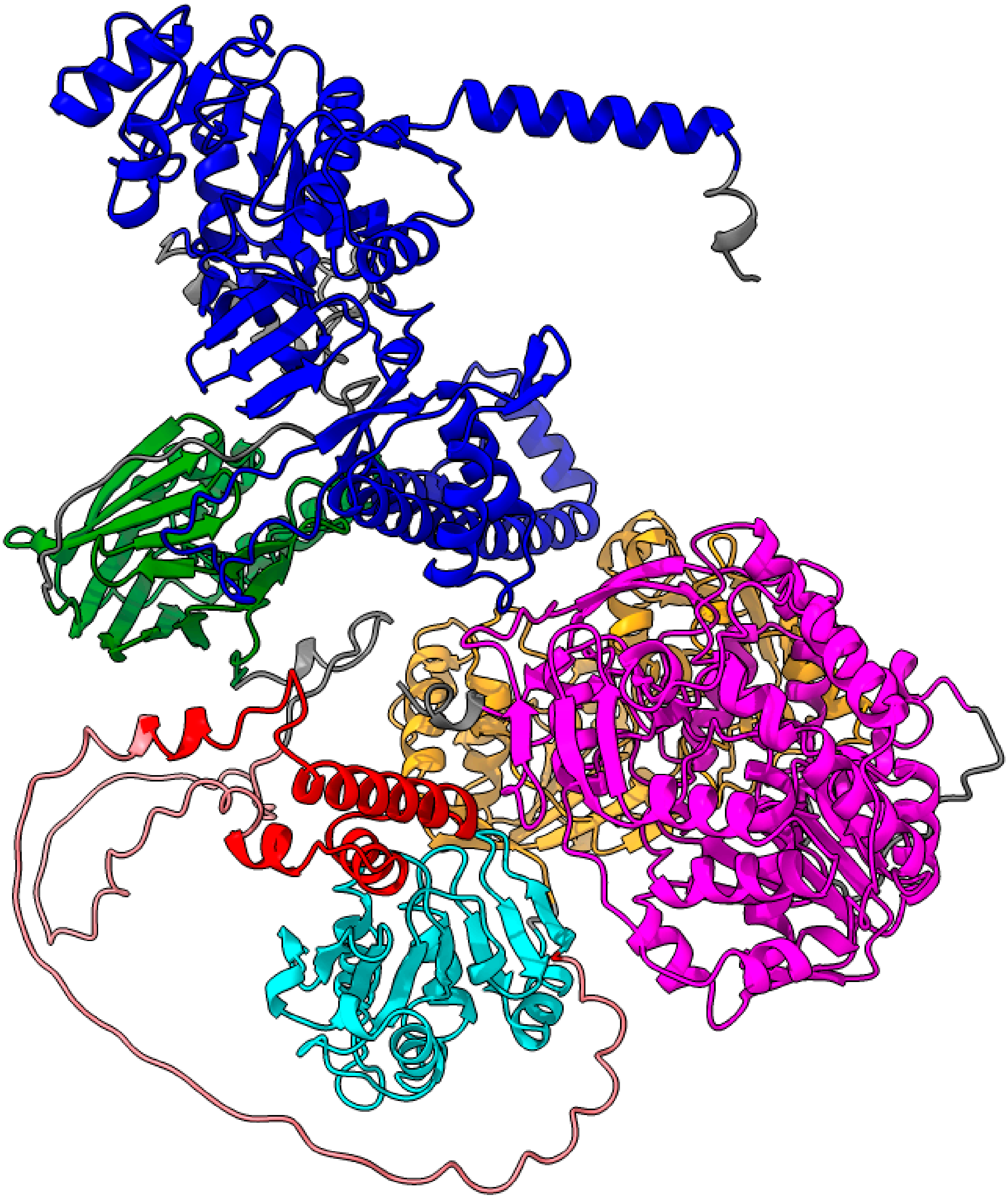

Supplementary Figure S4. RMSD with the first frame as reference. RMSD of WT (blue) and insertions (red) using the first frame as a reference. The X-axis indicates the molecular dynamics frame index, and the Y-axis indicates the RMSD in Å. All systems stabilized around frame 70,000 on average, marked with the black vertical lines.

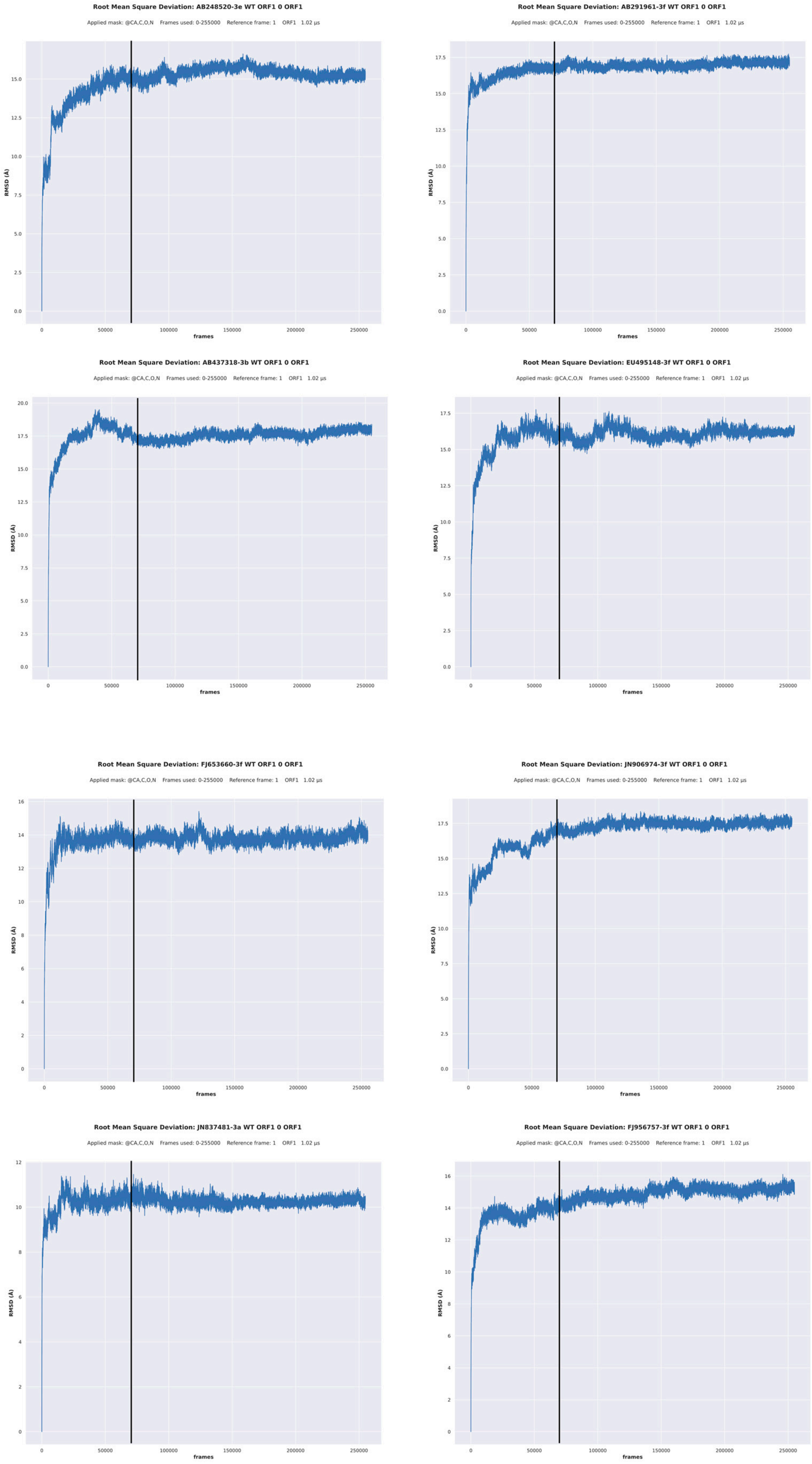

Root Mean Square Deviation: KT447527-3efg WT ORF1 0 ORF1

Applied mask: @CA,C,O,N   Frames used: 0-255000   Reference frame: 1   ORF1   1.02 µs

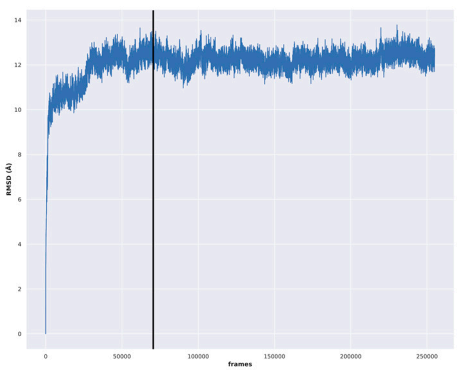

Root Mean Square Deviation: KT447528-3a WT ORF1 0 ORF1

Applied mask: @CA,C,O,N   Frames used: 0-255000   Reference frame: 1   ORF1   1.02 µs

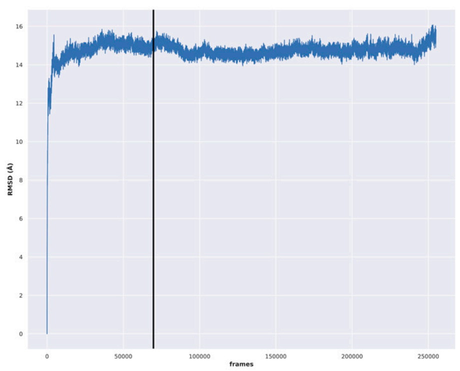

Root Mean Square Deviation: KU980235-3f WT ORF1 0 ORF1

Applied mask: @CA,C,O,N   Frames used: 0-255000   Reference frame: 1   ORF1   1.02 µs

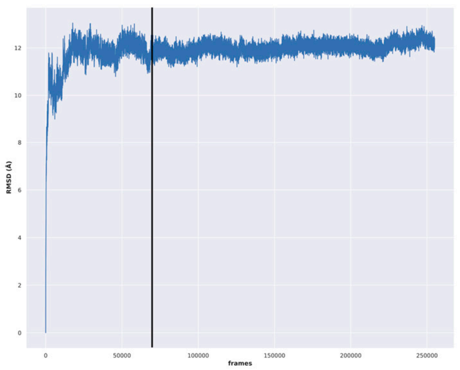

Root Mean Square Deviation: KY232312-3f WT ORF1 0 ORF1

Applied mask: @CA,C,O,N   Frames used: 0-255000   Reference frame: 1   ORF1   1.02 µs

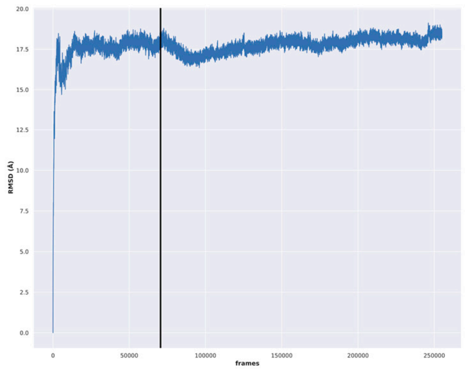

Root Mean Square Deviation: KY780957-3chi WT ORF1 0 ORF1

Applied mask: @CA,C,O,N   Frames used: 0-255000   Reference frame: 1   ORF1   1.02 µs

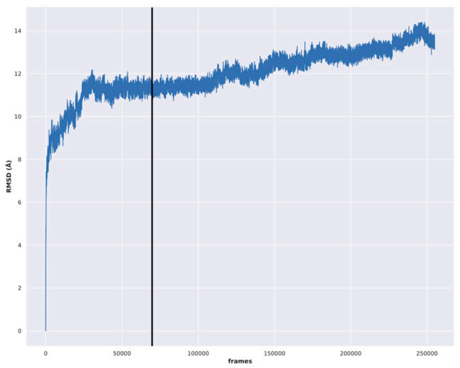

Root Mean Square Deviation: MF444031-3c WT ORF1 0 ORF1

Applied mask: @CA,C,O,N   Frames used: 0-255000   Reference frame: 1   ORF1   1.02 µs

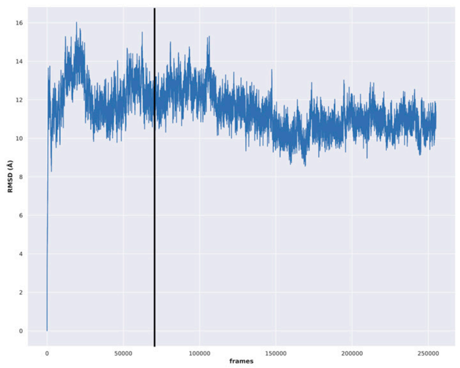

Root Mean Square Deviation: MG783569-3 WT ORF1 0 ORF1

Applied mask: @CA,C,O,N   Frames used: 0-255000   Reference frame: 1   ORF1   1.02 µs

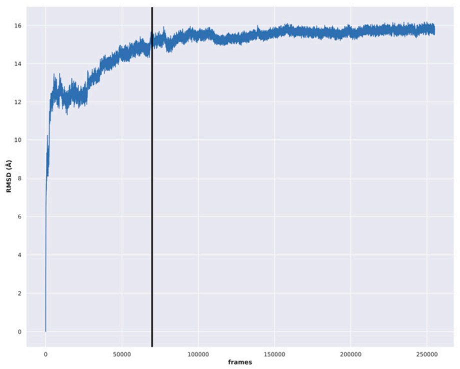

Root Mean Square Deviation: JQ679014 WT ORF1 0 ORF1

Applied mask: @CA,C,O,N   Frames used: 0-255000   Reference frame: 1   ORF1   1.02 µs

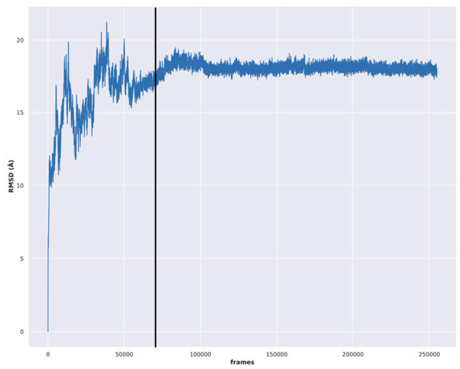

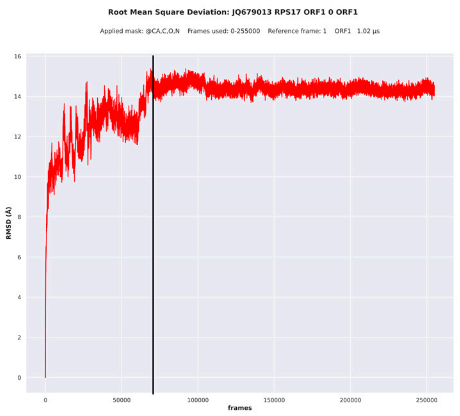
Root Mean Square Deviation: JQ679013 RPS17 ORF1 0 ORF1
Applied mask: @CA,C,O,N Frames used: 0-255000 Reference frame: 1 ORF1 1.02 µs
RMSD (Å)
frames

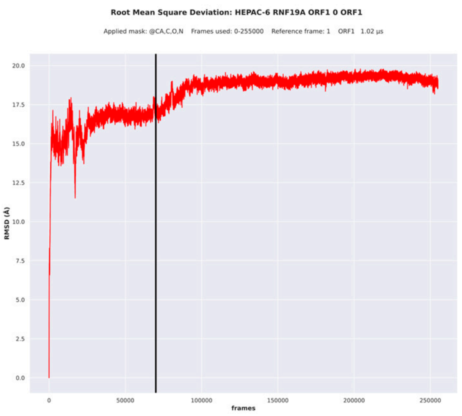
Root Mean Square Deviation: HEPAC-6 RNF19A ORF1 0 ORF1
Applied mask: @CA,C,O,N Frames used: 0-255000 Reference frame: 1 ORF1 1.02 µs
RMSD (Å)
frames

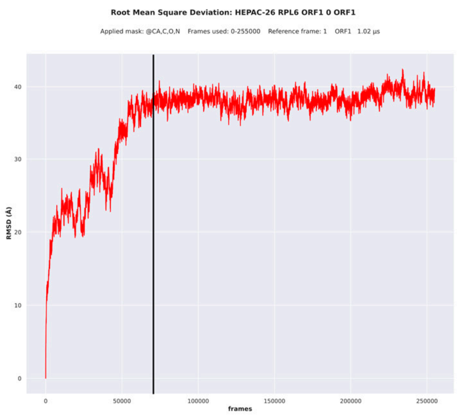
Root Mean Square Deviation: HEPAC-26 RPL6 ORF1 0 ORF1
Applied mask: @CA,C,O,N Frames used: 0-255000 Reference frame: 1 ORF1 1.02 µs
RMSD (Å)
frames

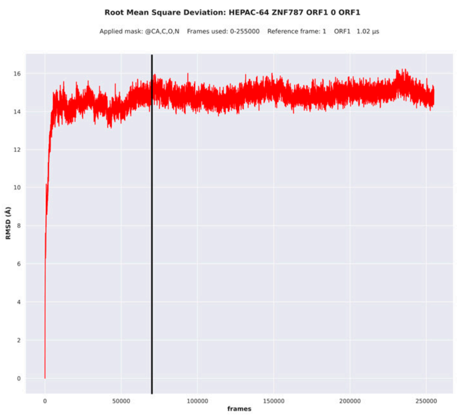
Root Mean Square Deviation: HEPAC-64 ZNF787 ORF1 0 ORF1
Applied mask: @CA,C,O,N Frames used: 0-255000 Reference frame: 1 ORF1 1.02 µs
RMSD (Å)
frames

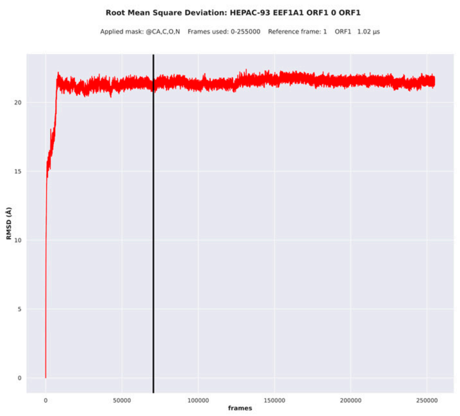
Root Mean Square Deviation: HEPAC-93 EEF1A1 ORF1 0 ORF1
Applied mask: @CA,C,O,N Frames used: 0-255000 Reference frame: 1 ORF1 1.02 µs
RMSD (Å)
frames

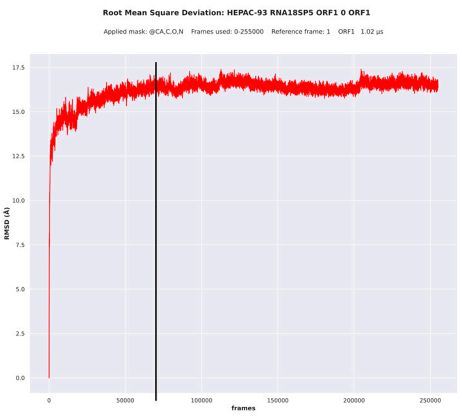
Root Mean Square Deviation: HEPAC-93 RNA18SP5 ORF1 0 ORF1
Applied mask: @CA,C,O,N Frames used: 0-255000 Reference frame: 1 ORF1 1.02 µs
RMSD (Å)
frames

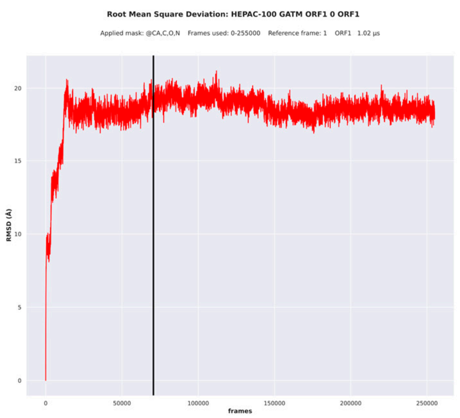
Root Mean Square Deviation: HEPAC-100 GATM ORF1 0 ORF1
Applied mask: @CA,C,O,N Frames used: 0-255000 Reference frame: 1 ORF1 1.02 µs
RMSD (Å)
frames

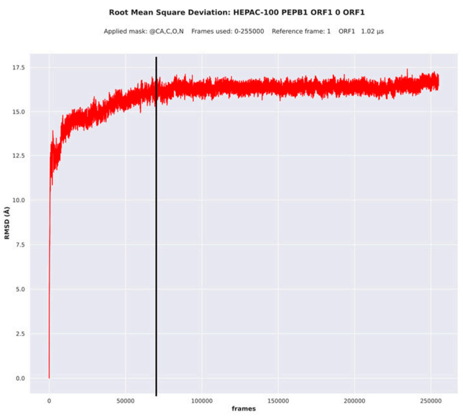
Root Mean Square Deviation: HEPAC-100 PEPB1 ORF1 0 ORF1
Applied mask: @CA,C,O,N Frames used: 0-255000 Reference frame: 1 ORF1 1.02 µs
RMSD (Å)
frames

Root Mean Square Deviation: HEPAC-154 KIF1B ORF1 0 ORF1

Applied mask: @CA,C,O,N   Frames used: 0-255000   Reference frame: 1   ORF1   1.02 µs

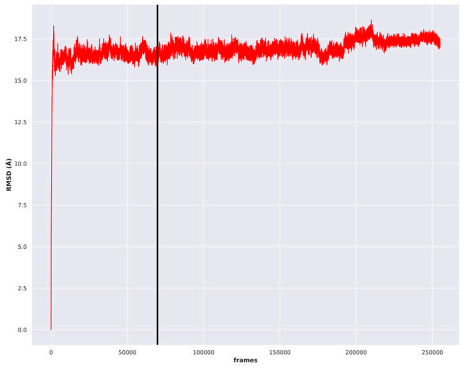

Supplementary Figure S5. WT (blue) and insertions (red) RMSD with the most representative frame from k-means clustering as reference. The X-axis indicates the molecular dynamics frame index starting at frame 70 000 to the last frame. The Y-axis indicates the RMSD in Å. The frame exhibiting an RMSD value of 0 Å corresponds to the representative structure identified through clustering and subsequently used as the reference frame.

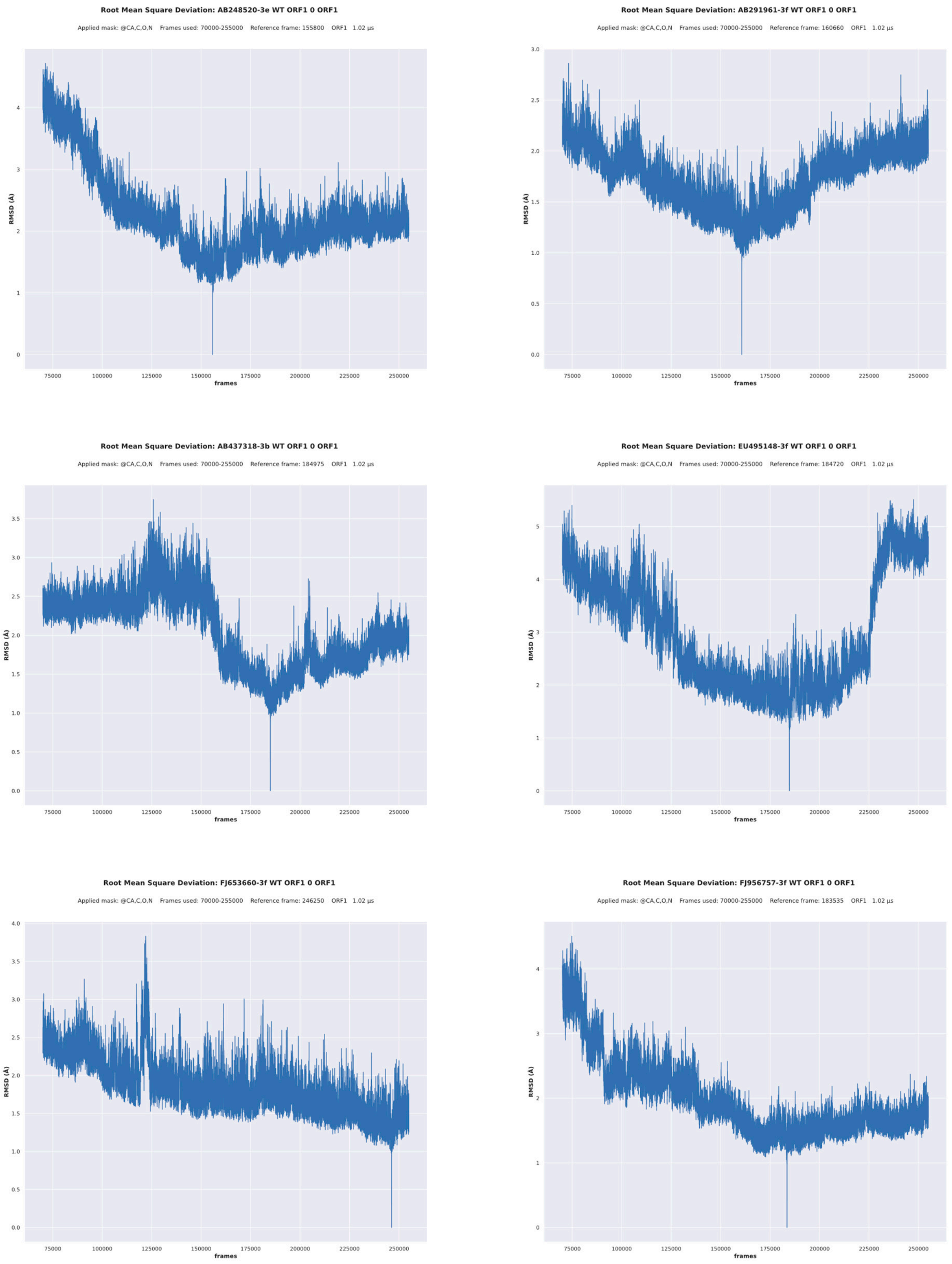

Root Mean Square Deviation: JN837481-3a WT ORF1 0 ORF1

Applied mask: @CA,C,O,N   Frames used: 70000-255000   Reference frame: 188920   ORF1   1.02 µs

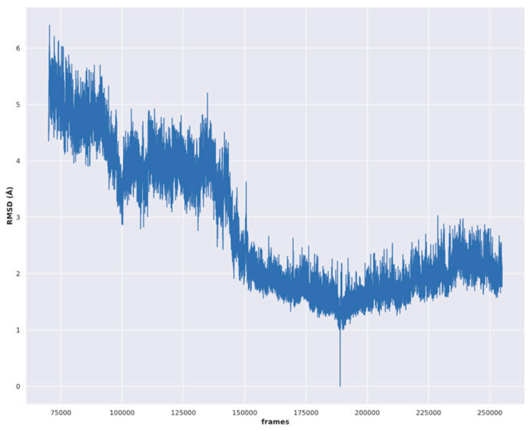

Root Mean Square Deviation: JN906974-3f WT ORF1 0 ORF1

Applied mask: @CA,C,O,N   Frames used: 70000-255000   Reference frame: 155590   ORF1   1.02 µs

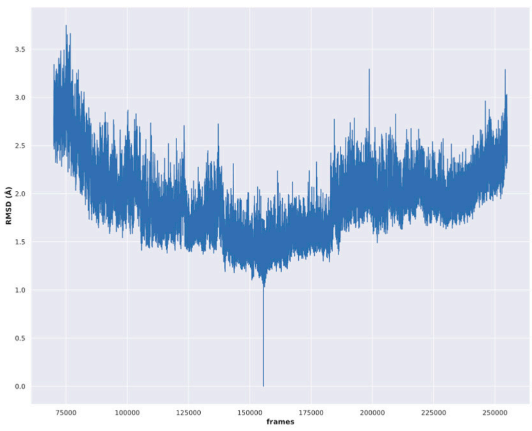

Root Mean Square Deviation: Kernow-C1-p1 WT ORF1 0 ORF1

Applied mask: @CA,C,O,N   Frames used: 70000-255000   Reference frame: 181210   ORF1   1.02 µs

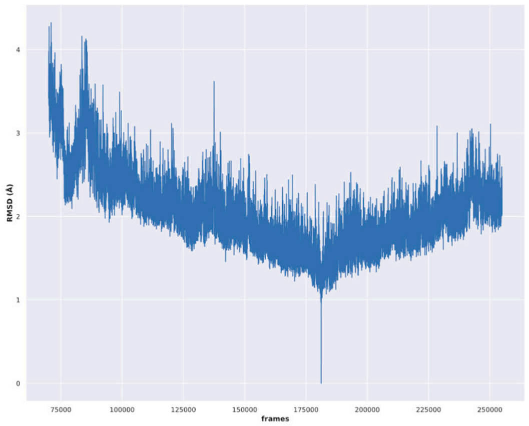

Root Mean Square Deviation: KT447527-3efg WT ORF1 0 ORF1

Applied mask: @CA,C,O,N   Frames used: 70000-255000   Reference frame: 210715   ORF1   1.02 µs

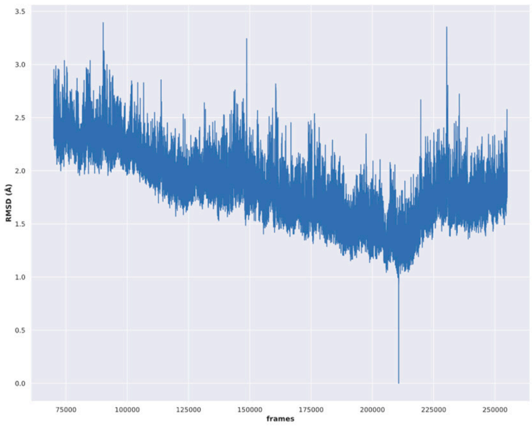

Root Mean Square Deviation: KT447528-3a WT ORF1 0 ORF1

Applied mask: @CA,C,O,N   Frames used: 70000-255000   Reference frame: 114265   ORF1   1.02 µs

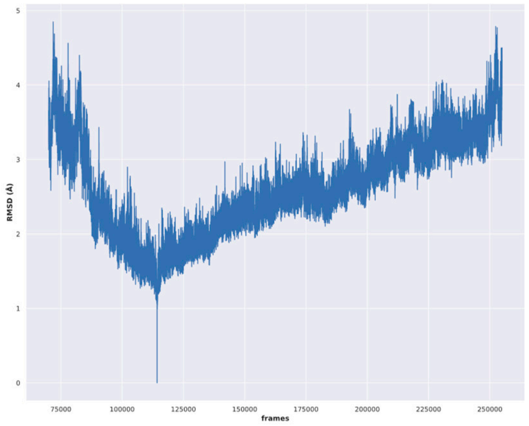

Root Mean Square Deviation: KU980235-3f WT ORF1 0 ORF1

Applied mask: @CA,C,O,N   Frames used: 70000-255000   Reference frame: 172525   ORF1   1.02 µs

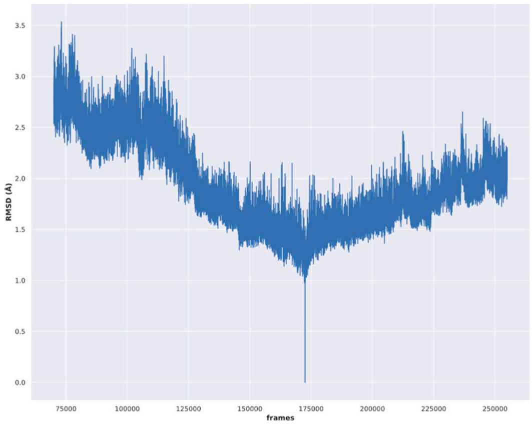

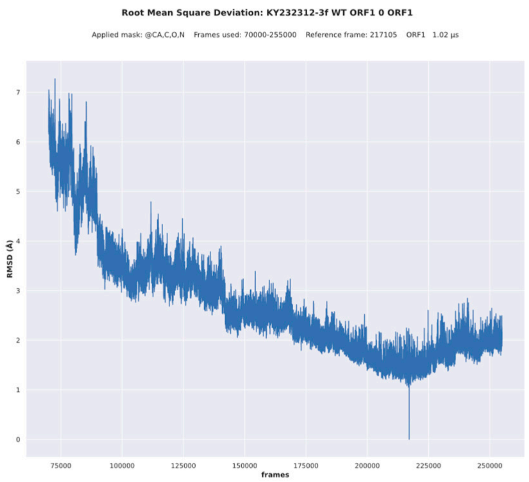
Root Mean Square Deviation: KY232312-3f WT ORF1 0 ORF1
Applied mask: @CA,C,O,N   Frames used: 70000-255000   Reference frame: 217105   ORF1   1.02 µs
RMSD (Å)
frames

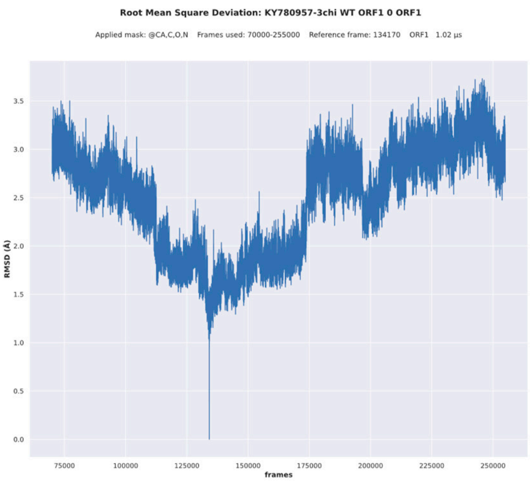
Root Mean Square Deviation: KY780957-3chi WT ORF1 0 ORF1
Applied mask: @CA,C,O,N   Frames used: 70000-255000   Reference frame: 134170   ORF1   1.02 µs
RMSD (Å)
frames

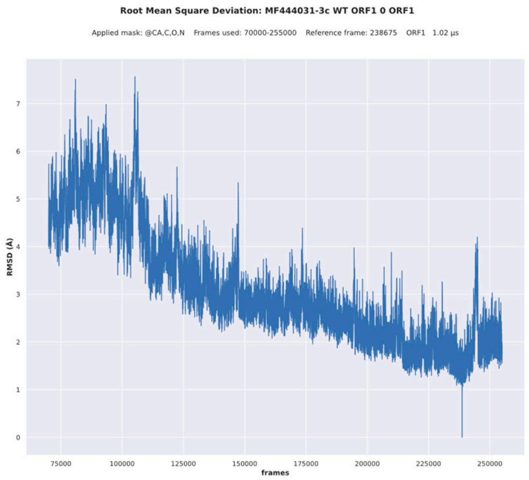
Root Mean Square Deviation: MF444031-3c WT ORF1 0 ORF1
Applied mask: @CA,C,O,N   Frames used: 70000-255000   Reference frame: 238675   ORF1   1.02 µs
RMSD (Å)
frames

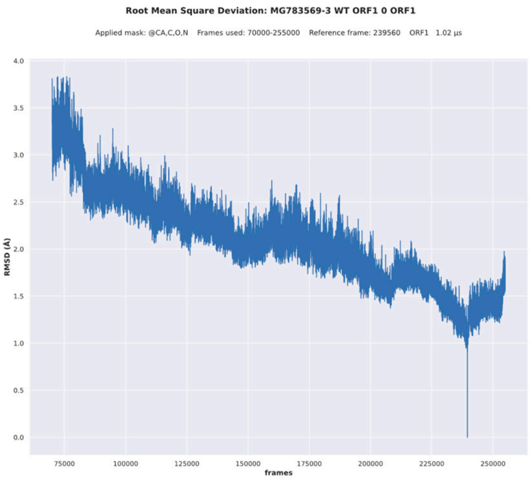
Root Mean Square Deviation: MG783569-3 WT ORF1 0 ORF1
Applied mask: @CA,C,O,N   Frames used: 70000-255000   Reference frame: 239560   ORF1   1.02 µs
RMSD (Å)
frames

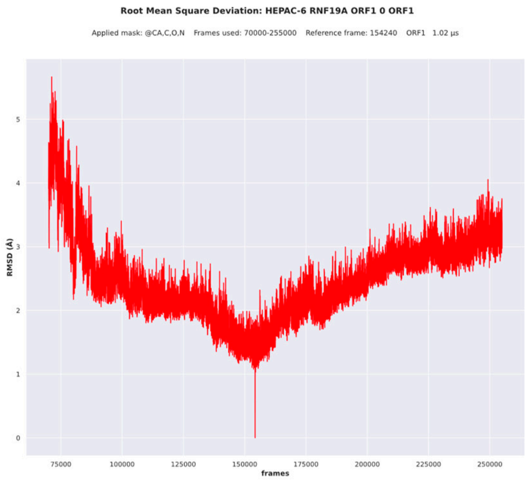
Root Mean Square Deviation: HEPAC-6 RNF19A ORF1 0 ORF1
Applied mask: @CA,C,O,N   Frames used: 70000-255000   Reference frame: 154240   ORF1   1.02 µs
RMSD (Å)
frames

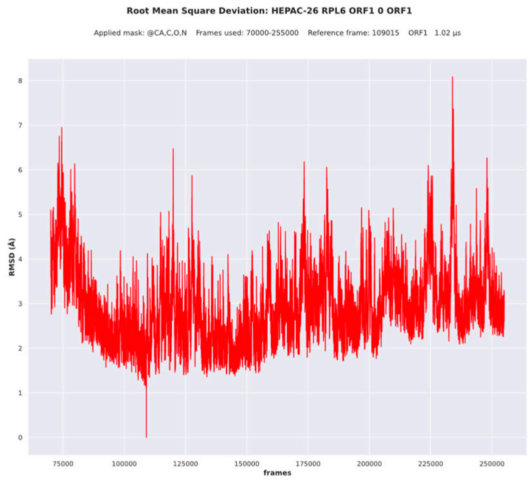
Root Mean Square Deviation: HEPAC-26 RPL6 ORF1 0 ORF1
Applied mask: @CA,C,O,N   Frames used: 70000-255000   Reference frame: 109015   ORF1   1.02 µs
RMSD (Å)
frames

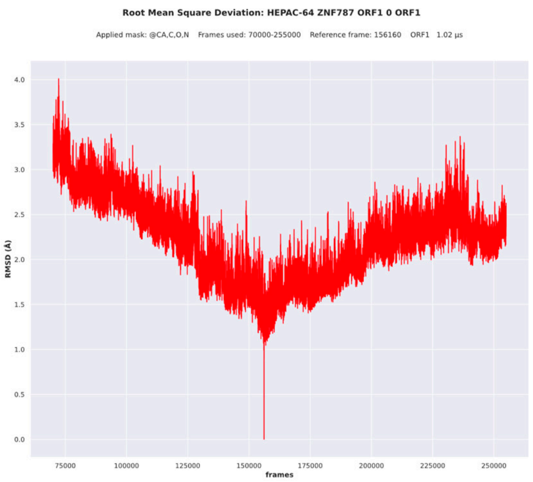
Root Mean Square Deviation: HEPAC-64 ZNF787 ORF1 0 ORF1
Applied mask: @CA,C,O,N Frames used: 70000-255000 Reference frame: 156160 ORF1 1.02 µs
RMSD (Å)
frames

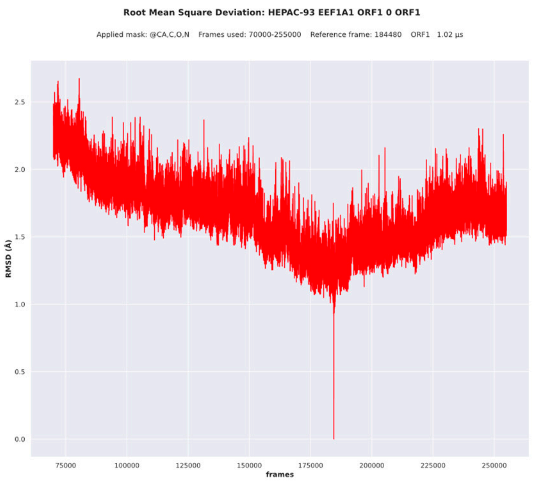
Root Mean Square Deviation: HEPAC-93 EEF1A1 ORF1 0 ORF1
Applied mask: @CA,C,O,N Frames used: 70000-255000 Reference frame: 184480 ORF1 1.02 µs
RMSD (Å)
frames

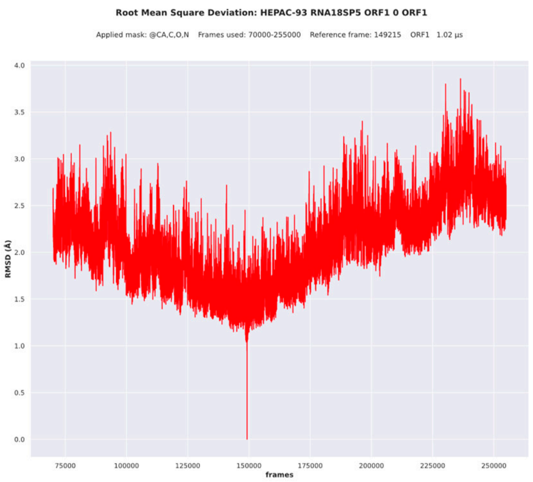
Root Mean Square Deviation: HEPAC-93 RNA18SP5 ORF1 0 ORF1
Applied mask: @CA,C,O,N Frames used: 70000-255000 Reference frame: 149215 ORF1 1.02 µs
RMSD (Å)
frames

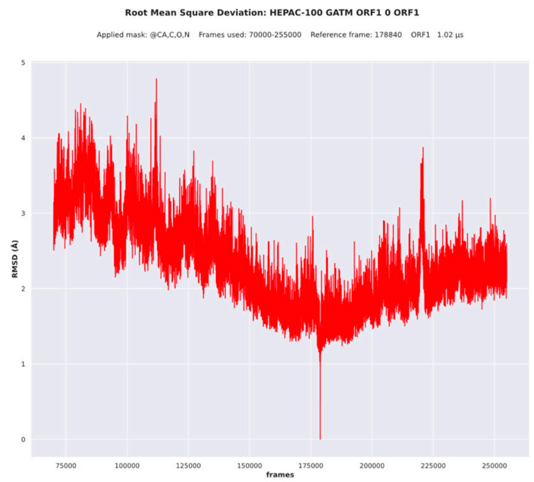
Root Mean Square Deviation: HEPAC-100 GATM ORF1 0 ORF1
Applied mask: @CA,C,O,N Frames used: 70000-255000 Reference frame: 178840 ORF1 1.02 µs
RMSD (Å)
frames

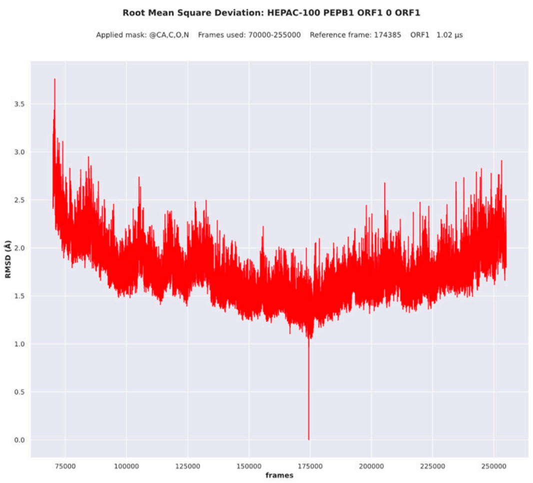
Root Mean Square Deviation: HEPAC-100 PEPB1 ORF1 0 ORF1
Applied mask: @CA,C,O,N Frames used: 70000-255000 Reference frame: 174385 ORF1 1.02 µs
RMSD (Å)
frames

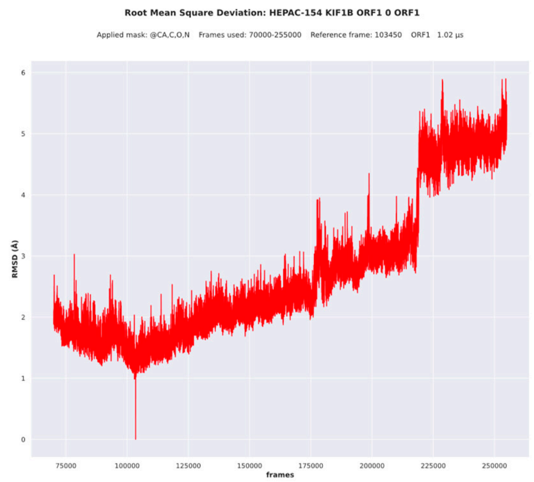
Root Mean Square Deviation: HEPAC-154 KIF1B ORF1 0 ORF1
Applied mask: @CA,C,O,N Frames used: 70000-255000 Reference frame: 103450 ORF1 1.02 µs
RMSD (Å)
frames

Root Mean Square Deviation:Kernow-C1-p6 RPS17 ORF1 0 ORF1

Applied mask: @CA,C,O,N   Frames used: 70000-255000   Reference frame: 184120   ORF1   1.02 µs

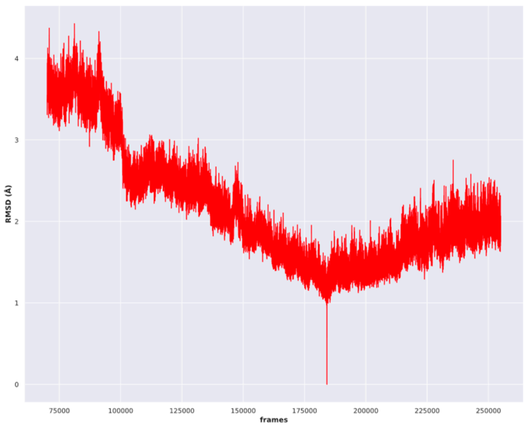

Supplementary Figure S6. RMSF comparison among strains with insertions and WT sequences across the pORF1 domains. The RMSF means and 95% confidence intervals are shown for each group (red: insertions, blue: WT). The PPR was excluded, as the RMSF comparisons require identical amino acid sequences length. (a) MetY domain. (b) FABD/MBD. (c) Macro domain. (d) Helicase domain. (e) RNA-dependent RNA polymerase domain. The RMSF figures for the Macro domain, Helicase, and RdRp are numbered starting from position 1, since the sequence alignment contains insertions preceding the domain positions.

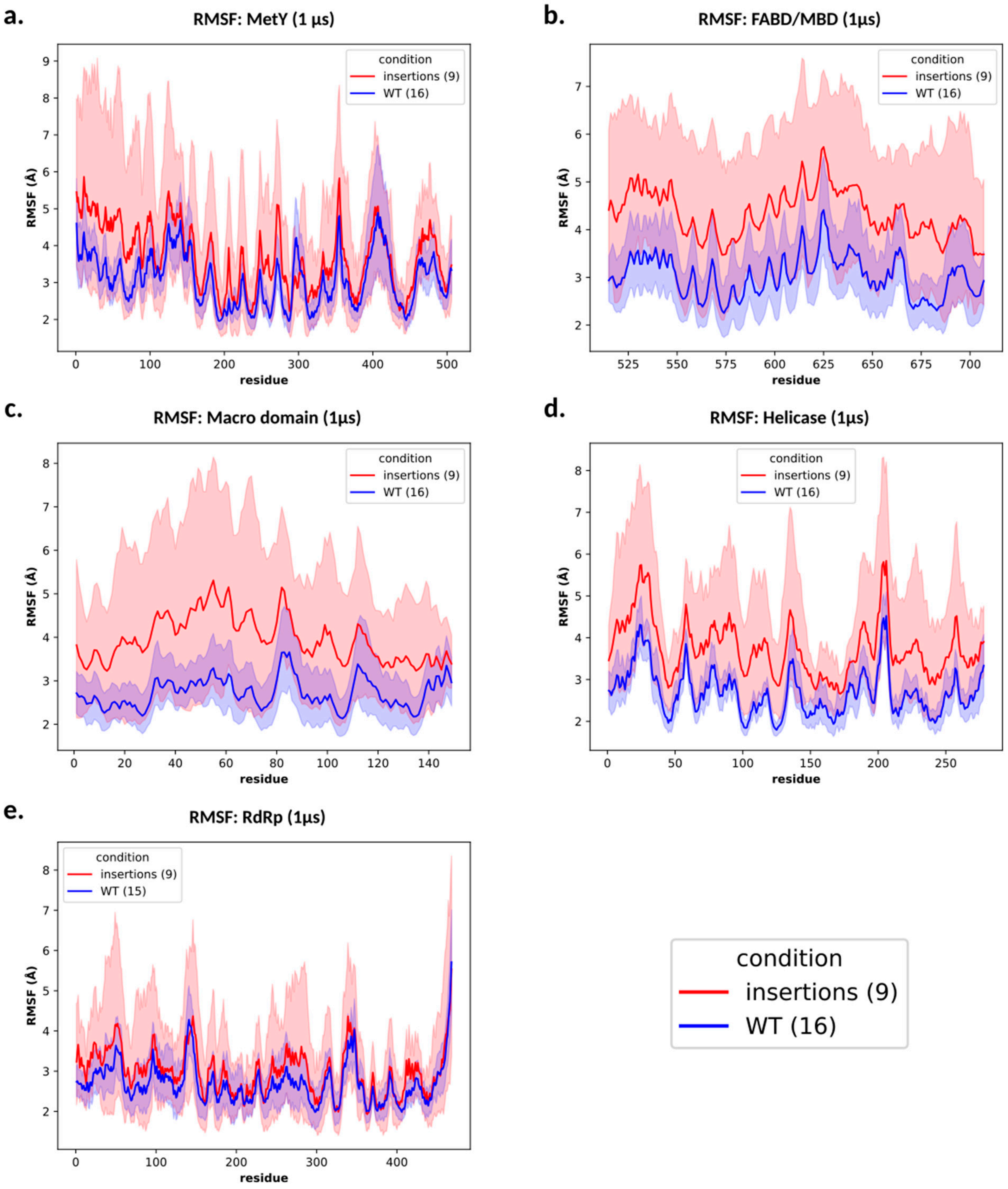

Supplementary Figure S7. Hydrogen bonds formation between the PPR and the pORF1 for the 16 WT strains and the 9 strains with insertions.

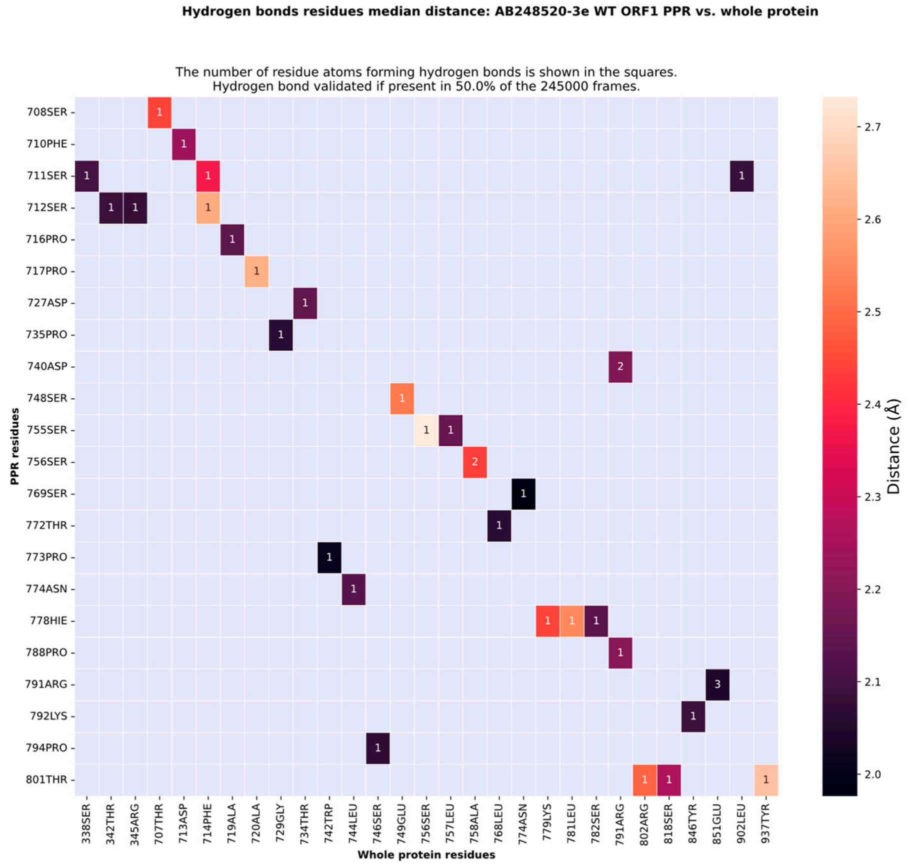

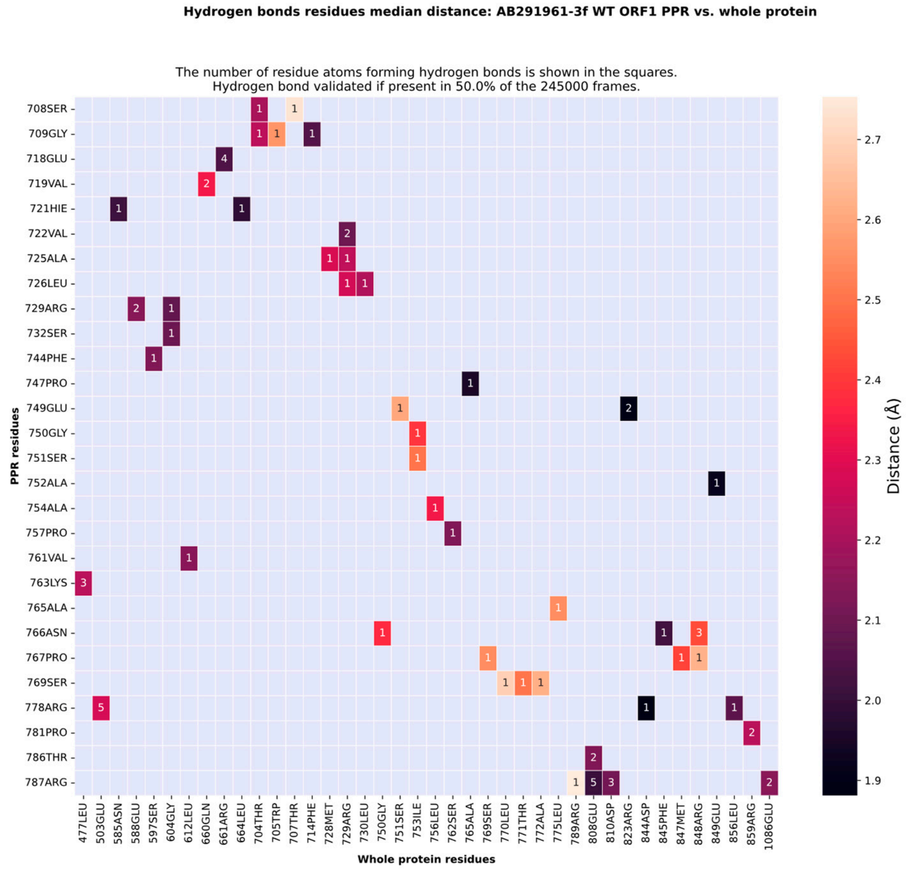

Hydrogen bonds residues median distance: AB437318-3b WT ORF1 PPR vs. whole protein

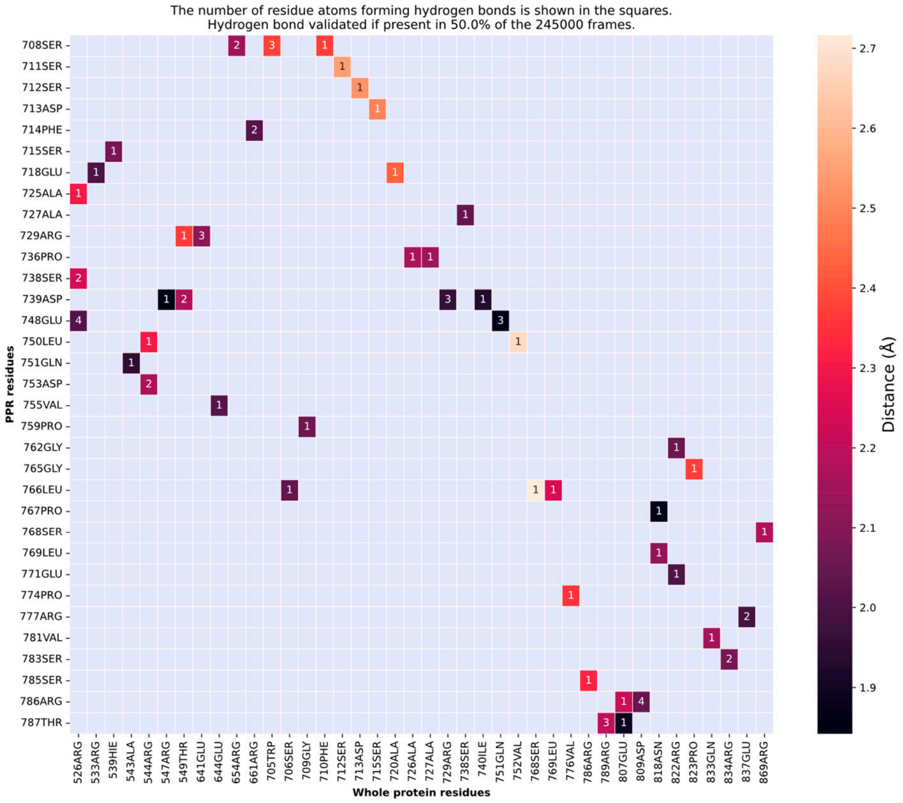

Hydrogen bonds residues median distance: EU495148-3f WT ORF1 PPR vs. whole protein

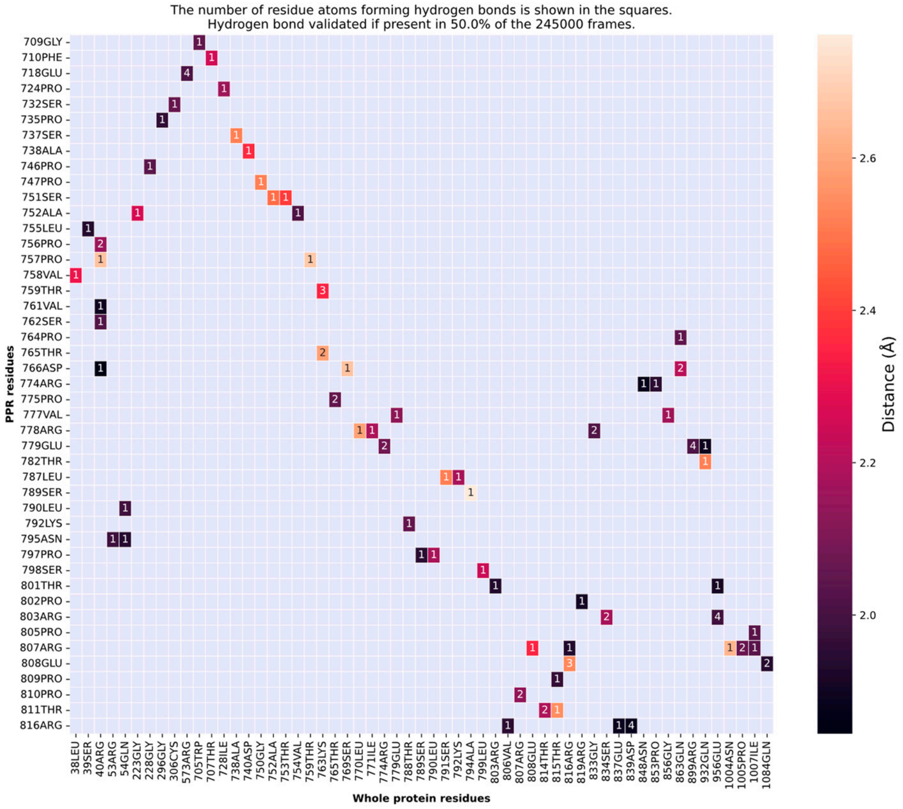

Hydrogen bonds residues median distance: FJ653660-3f WT ORF1 PPR vs. whole protein

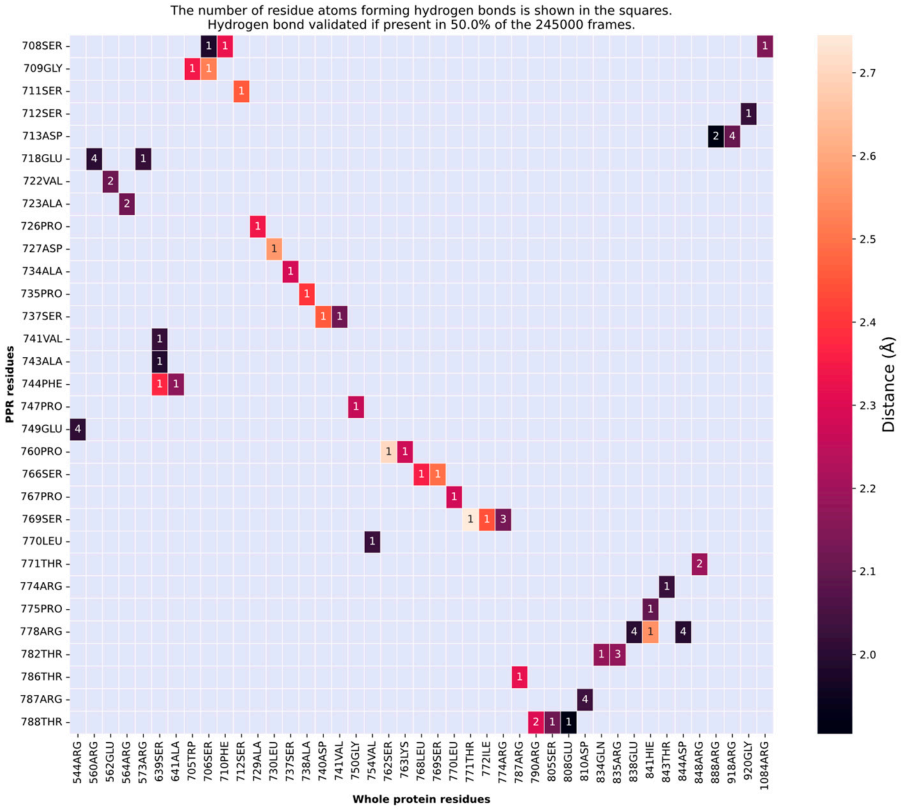

Hydrogen bonds residues median distance: FJ956757-3f WT ORF1 PPR vs. whole protein

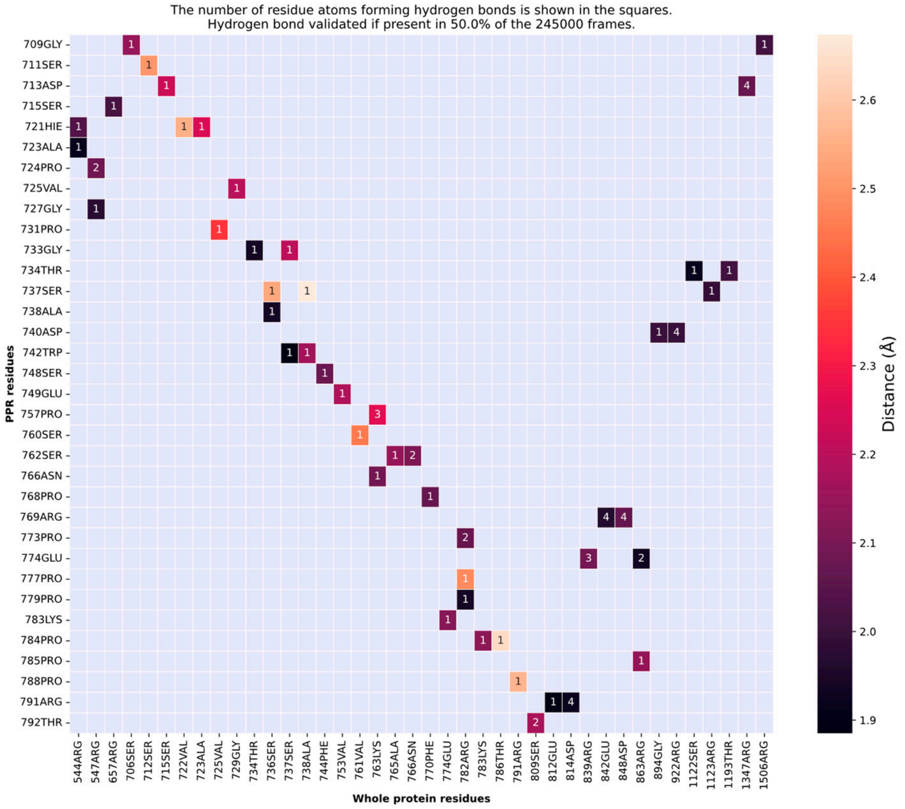

Hydrogen bonds residues median distance: JN837481-3a WT ORF1 PPR vs. whole protein

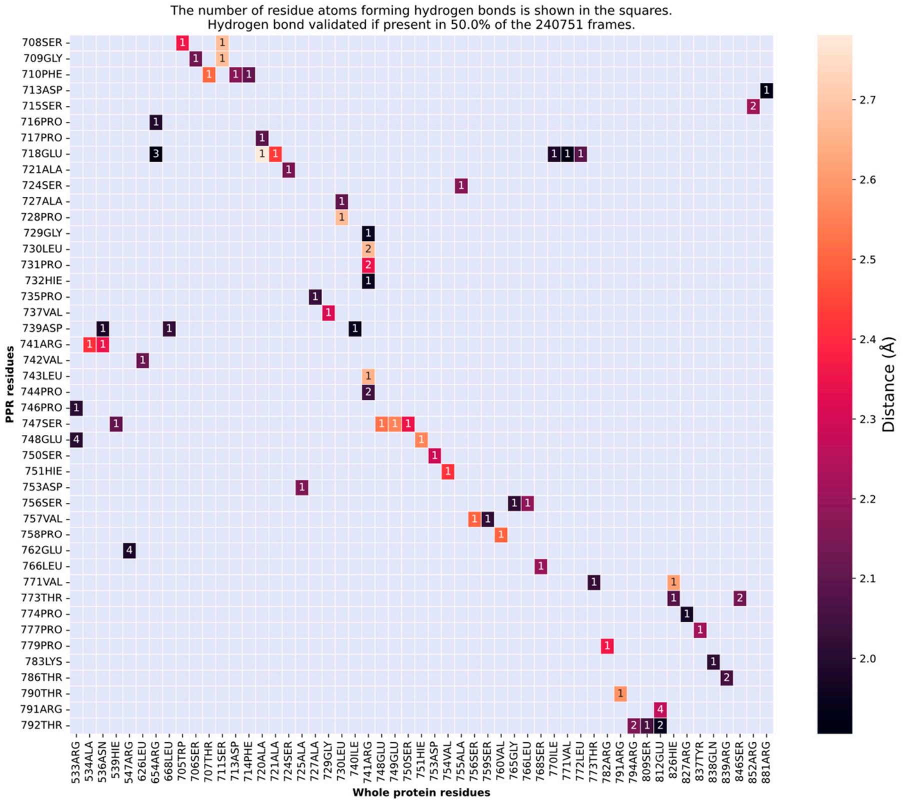

Hydrogen bonds residues median distance: JN906974-3f WT ORF1 PPR vs. whole protein

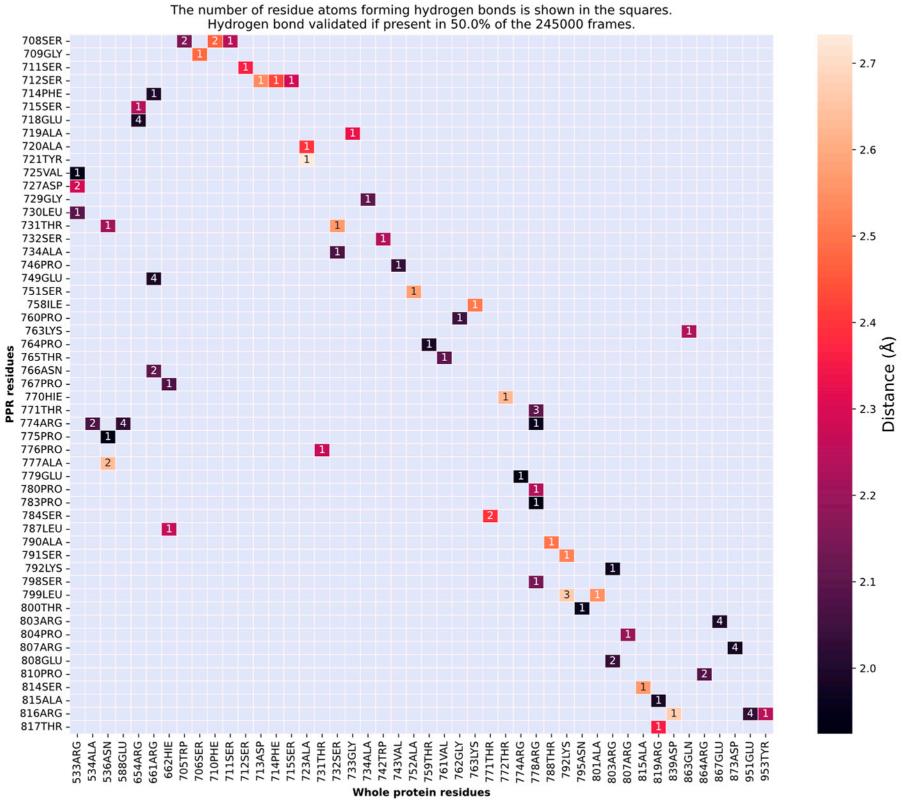

Hydrogen bonds residues median distance: Kernow-C1-p1 WT ORF1 PPR vs. whole protein

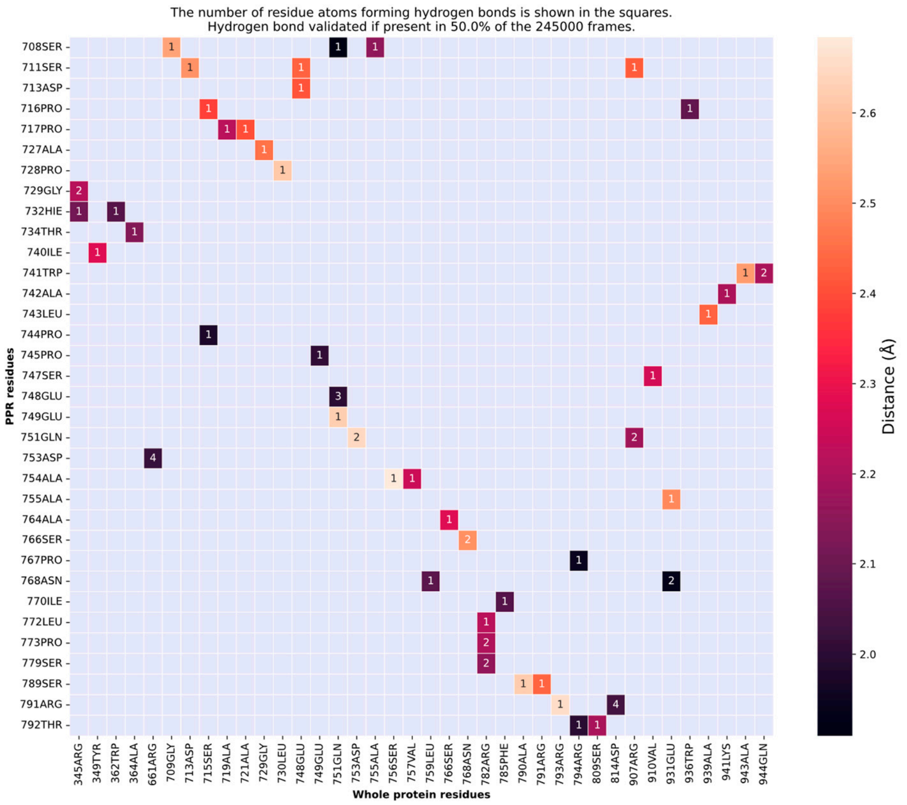

Hydrogen bonds residues median distance: KT447527-3f WT ORF1 PPR vs. whole protein

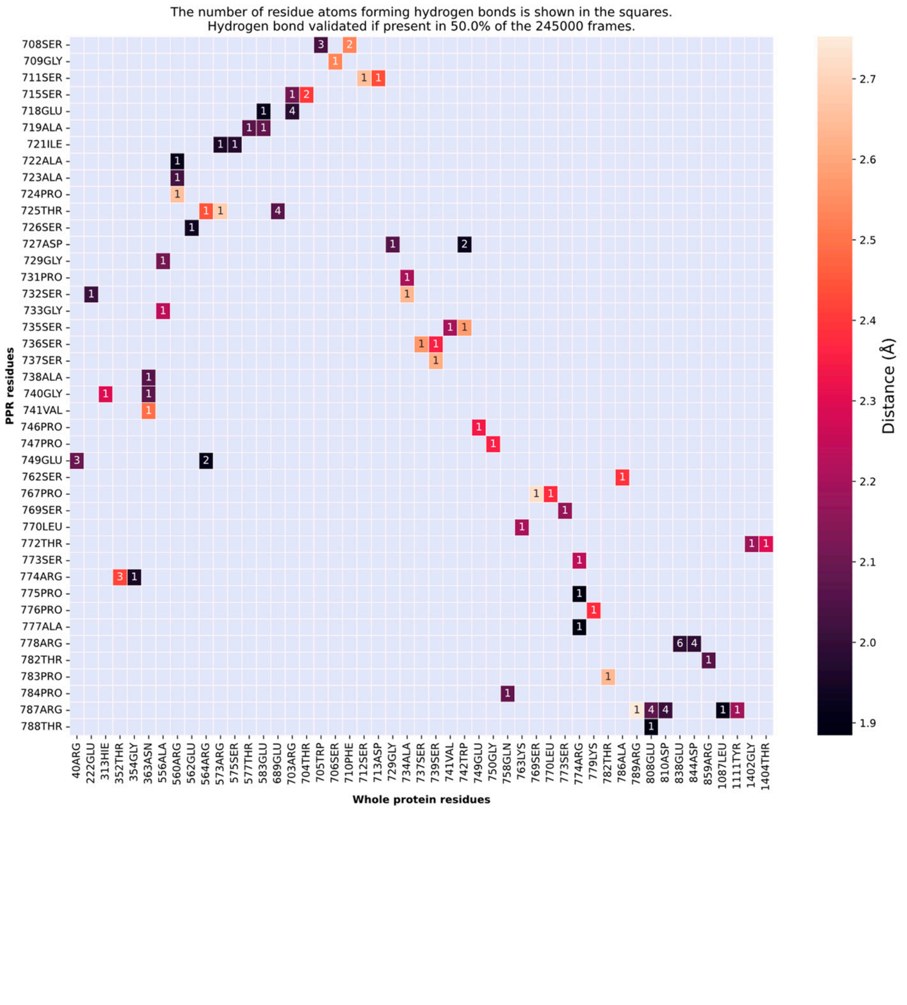

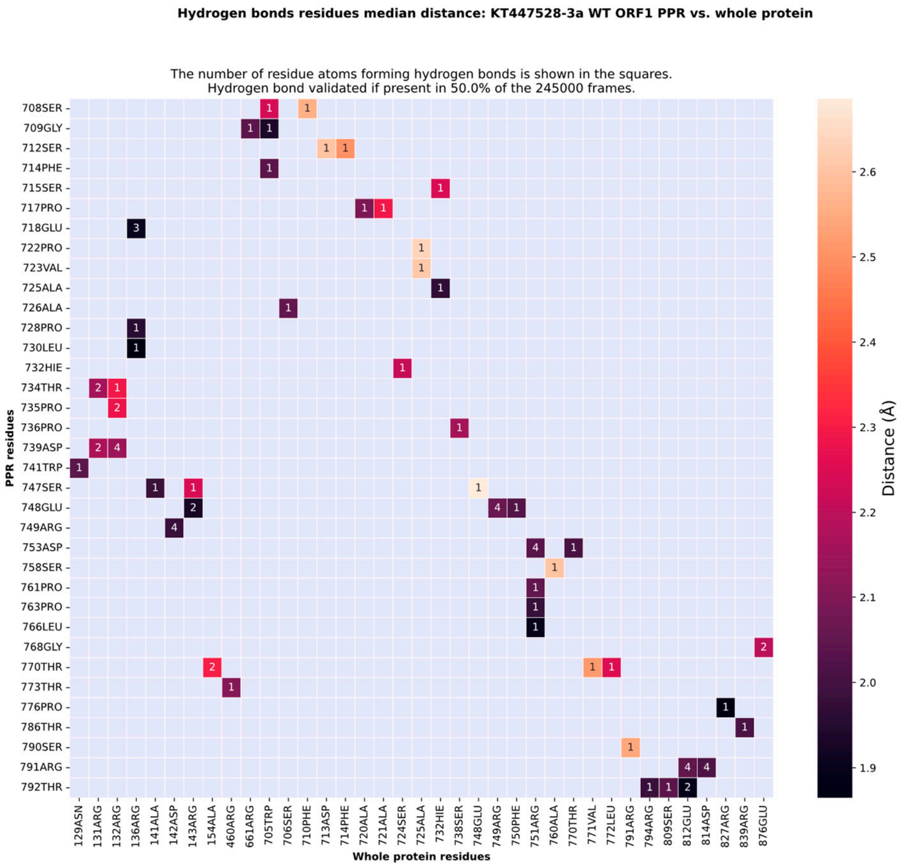
Hydrogen bonds residues median distance: KT447528-3a WT ORF1 PPR vs. whole protein
The number of residue atoms forming hydrogen bonds is shown in the squares.
Hydrogen bond validated if present in 50.0% of the 245000 frames.
PPR residues
Whole protein residues
Distance (Å)

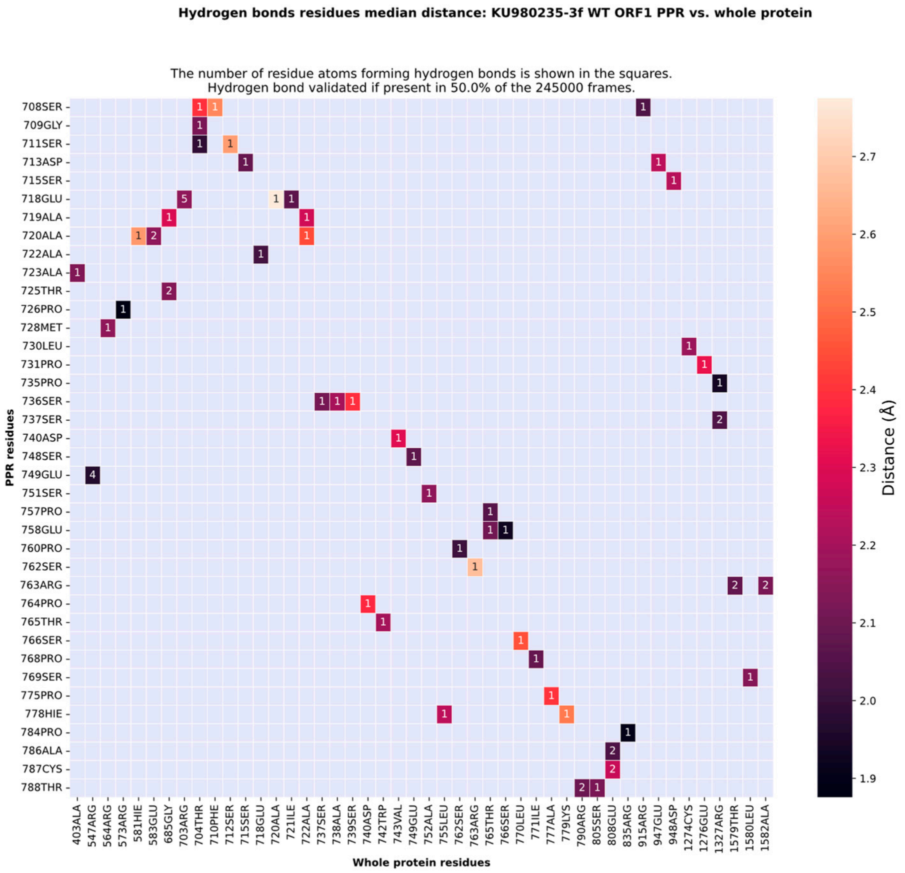
Hydrogen bonds residues median distance: KU980235-3f WT ORF1 PPR vs. whole protein
The number of residue atoms forming hydrogen bonds is shown in the squares.
Hydrogen bond validated if present in 50.0% of the 245000 frames.
PPR residues
Whole protein residues
Distance (Å)

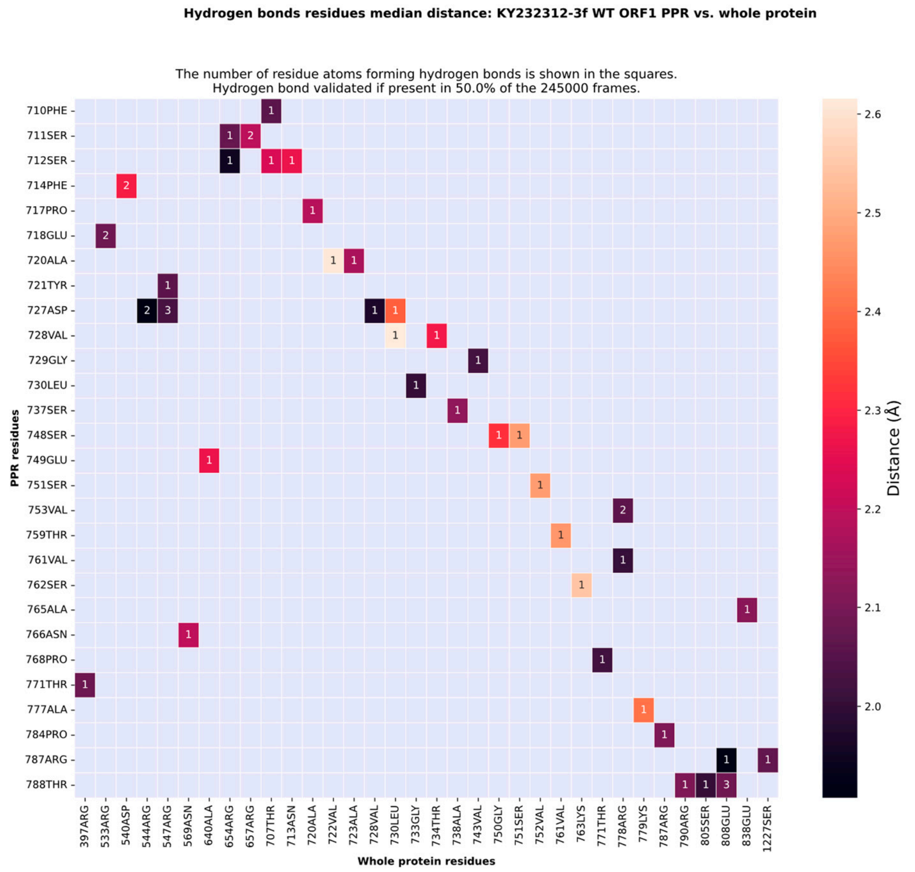
Hydrogen bonds residues median distance: KY232312-3f WT ORF1 PPR vs. whole protein
The number of residue atoms forming hydrogen bonds is shown in the squares.
Hydrogen bond validated if present in 50.0% of the 245000 frames.
PPR residues
710PHE
711SER
712SER
714PHE
717PRO
718GLU
720ALA
721TYR
727ASP
728VAL
729GLY
730LEU
737SER
748SER
749GLU
751SER
753VAL
759THR
761VAL
762SER
765ALA
766ASN
768PRO
771THR
777ALA
784PRO
787ARG
788THR
Whole protein residues
397ARG
533ARG
540ASP
544ARG
547ARG
569ASN
640ALA
654ARG
657ARG
707THR
713ASN
720ALA
722VAL
723ALA
728VAL
730LEU
733GLY
734THR
738ALA
743VAL
750GLY
751SER
752VAL
761VAL
763LYS
771THR
778ARG
779LYS
787ARG
790ARG
805SER
808GLU
838GLU
1227SER
Distance (Å)

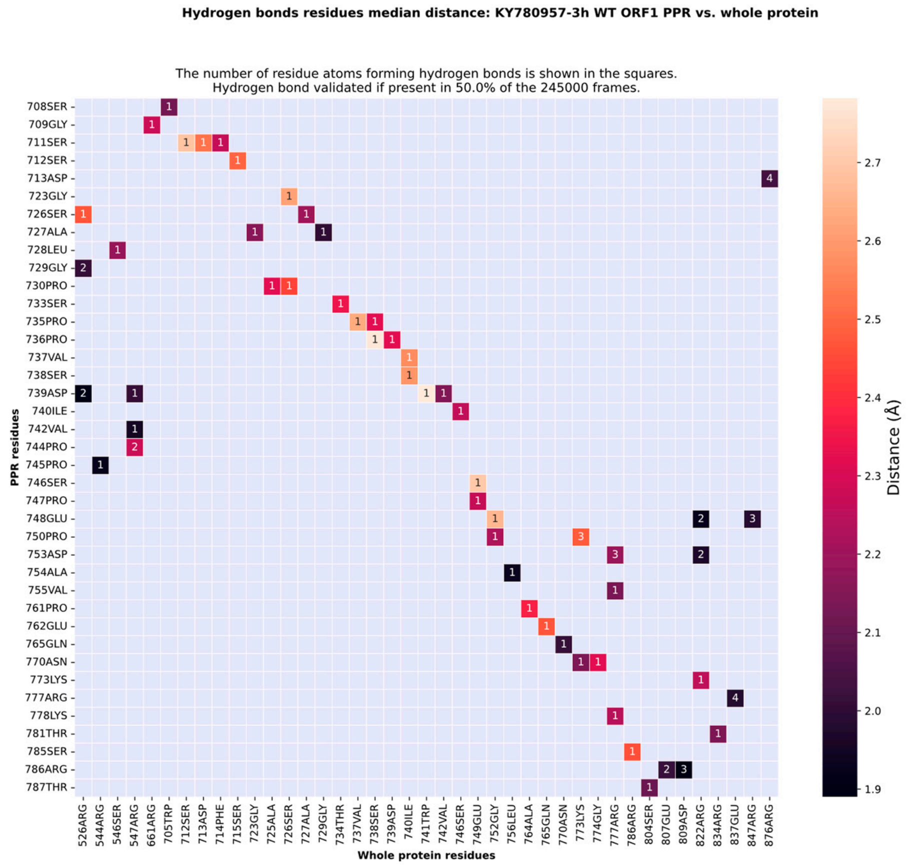
Hydrogen bonds residues median distance: KY780957-3h WT ORF1 PPR vs. whole protein
The number of residue atoms forming hydrogen bonds is shown in the squares.
Hydrogen bond validated if present in 50.0% of the 245000 frames.
PPR residues
708SER
709GLY
711SER
712SER
713ASP
723GLY
726SER
727ALA
728LEU
729GLY
730PRO
733SER
735PRO
736PRO
737VAL
738SER
739ASP
740ILE
742VAL
744PRO
745PRO
746SER
747PRO
748GLU
750PRO
753ASP
754ALA
755VAL
761PRO
762GLU
765GLN
770ASN
773LYS
777ARG
778LYS
781THR
785SER
786ARG
787THR
Whole protein residues
526ARG
544ARG
546SER
547ARG
661ARG
705TRP
712SER
713ASP
714PHE
715SER
723GLY
725ALA
726SER
727ALA
729GLY
734THR
737VAL
738SER
739ASP
740ILE
741TRP
742VAL
746SER
749GLU
752GLY
756LEU
764ALA
765GLN
770ASN
773LYS
774GLY
777ARG
786ARG
804SER
807GLU
809ASP
822ARG
834ARG
837GLU
847ARG
876ARG
Distance (Å)

Hydrogen bonds residues median distance: MF444031-3c WT ORF1 PPR vs. whole protein

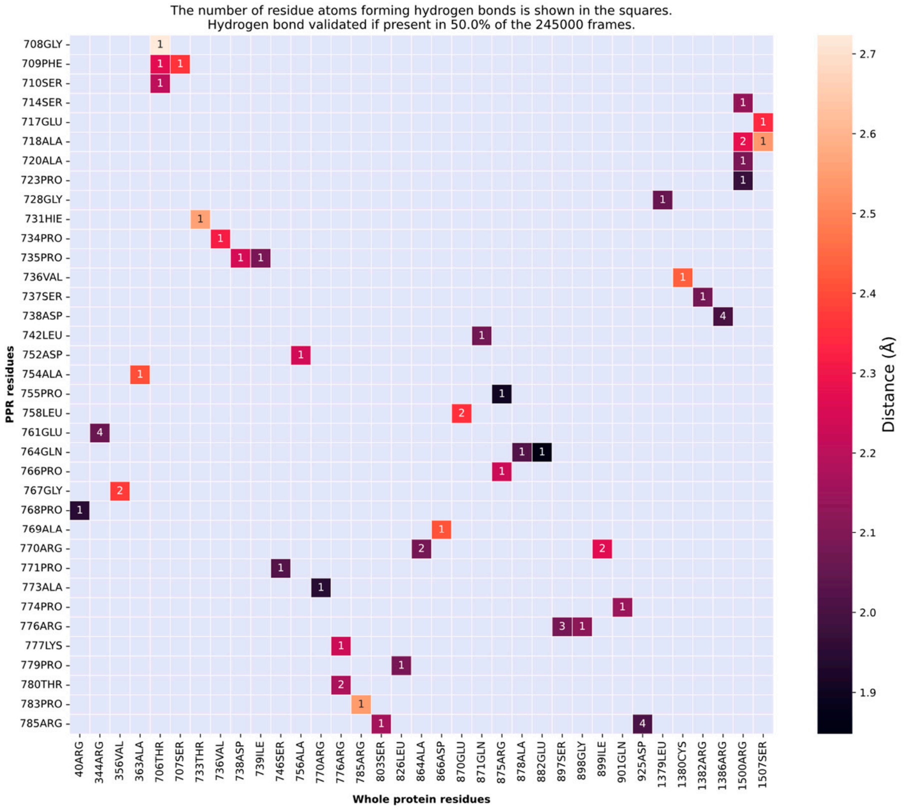

Hydrogen bonds residues median distance: MG783569-3c WT ORF1 PPR vs. whole protein

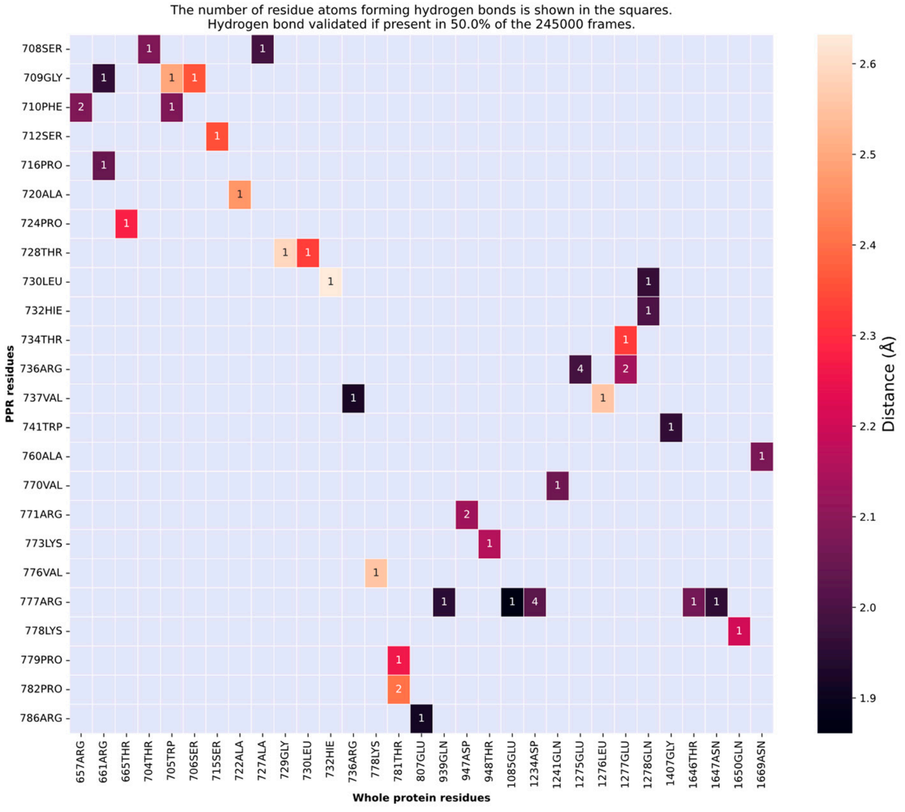

Hydrogen bonds residues median distance: HEPAC-6 RNF19A ORF1 PPR vs. whole protein

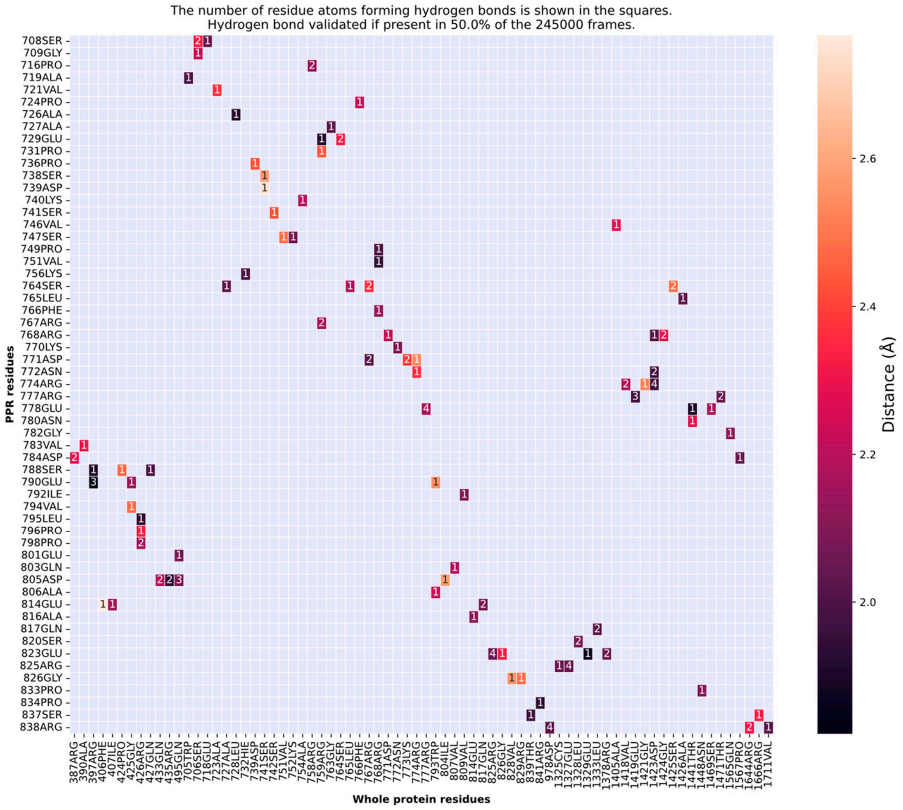

Hydrogen bonds residues median distance: HEPAC-26 RPL6 ORF1 PPR vs. whole protein

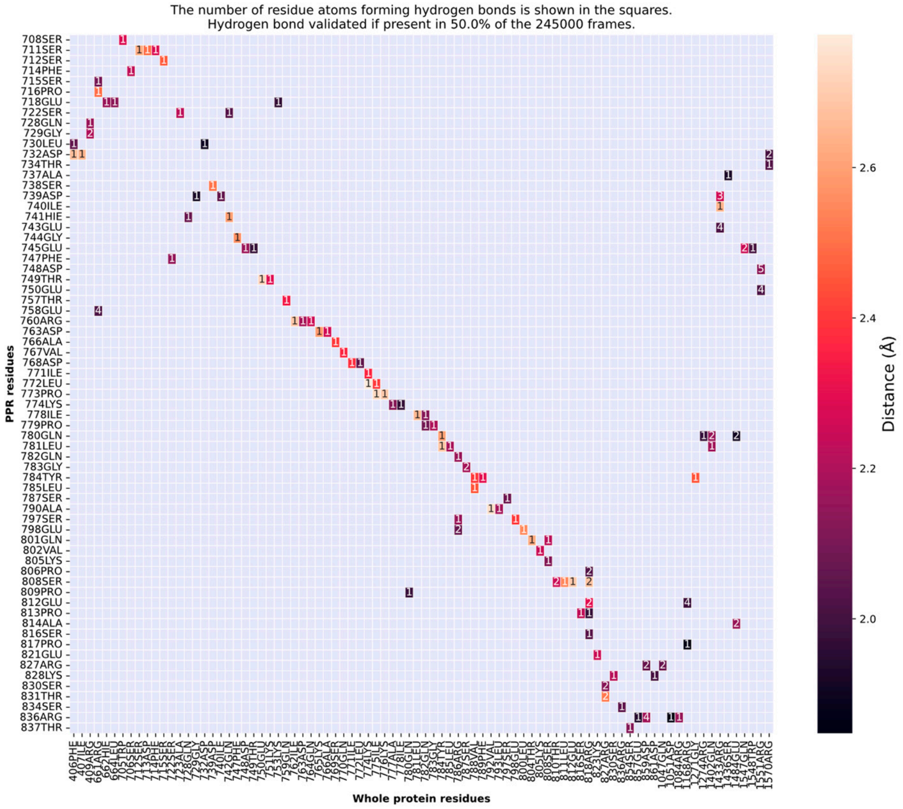

Hydrogen bonds residues median distance: HEPAC-64 ZNF787 ORF1 PPR vs. whole protein

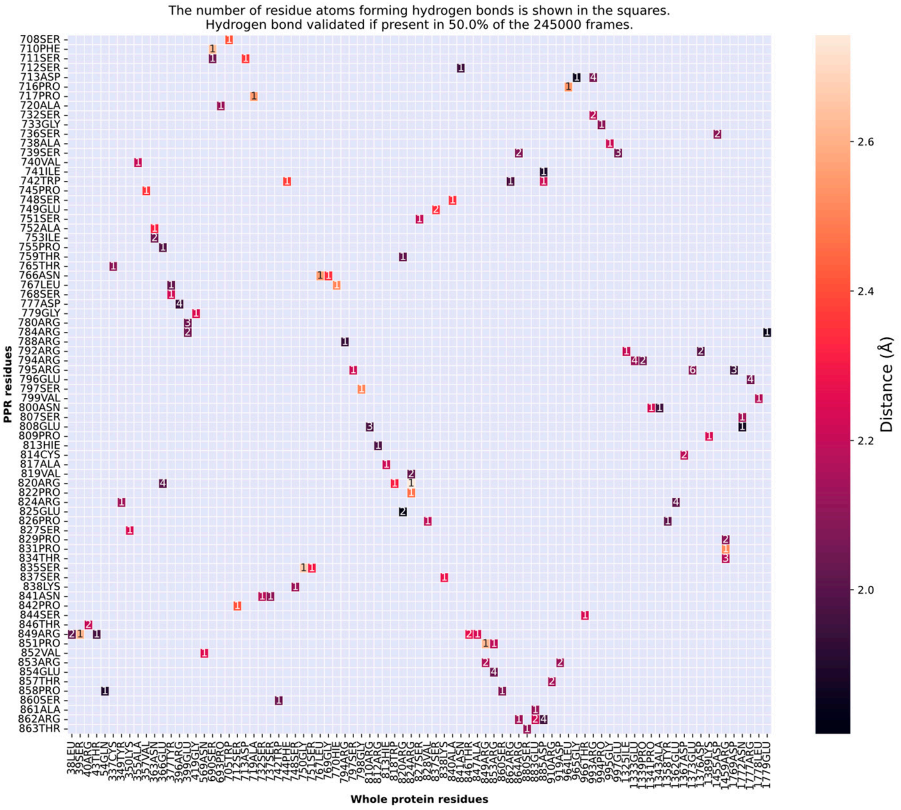

Hydrogen bonds residues median distance: HEPAC-93 EEF1A1 ORF1 PPR vs. whole protein

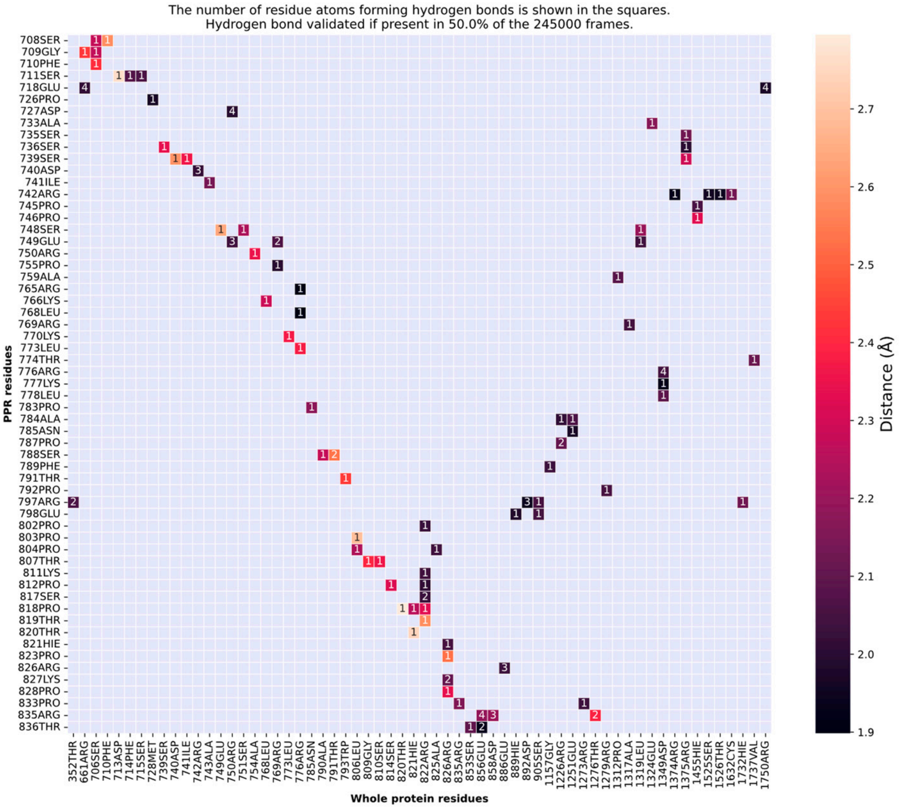

Hydrogen bonds residues median distance: HEPAC-93 RNA18SP5 ORF1 PPR vs. whole protein

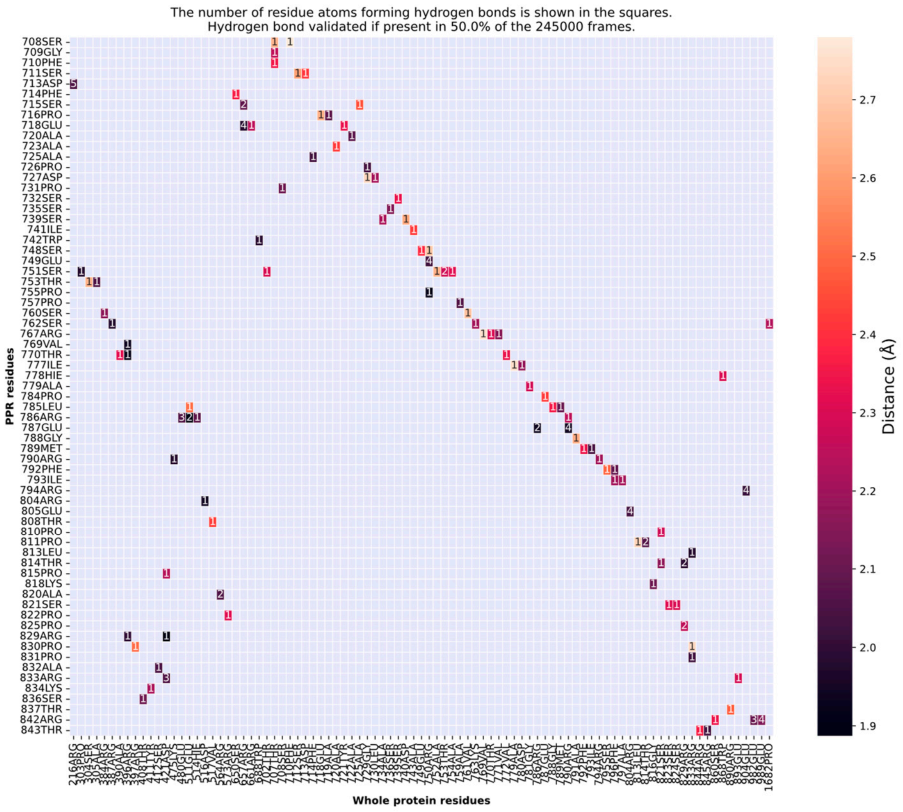

Hydrogen bonds residues median distance: HEPAC-100 GATM ORF1 PPR vs. whole protein

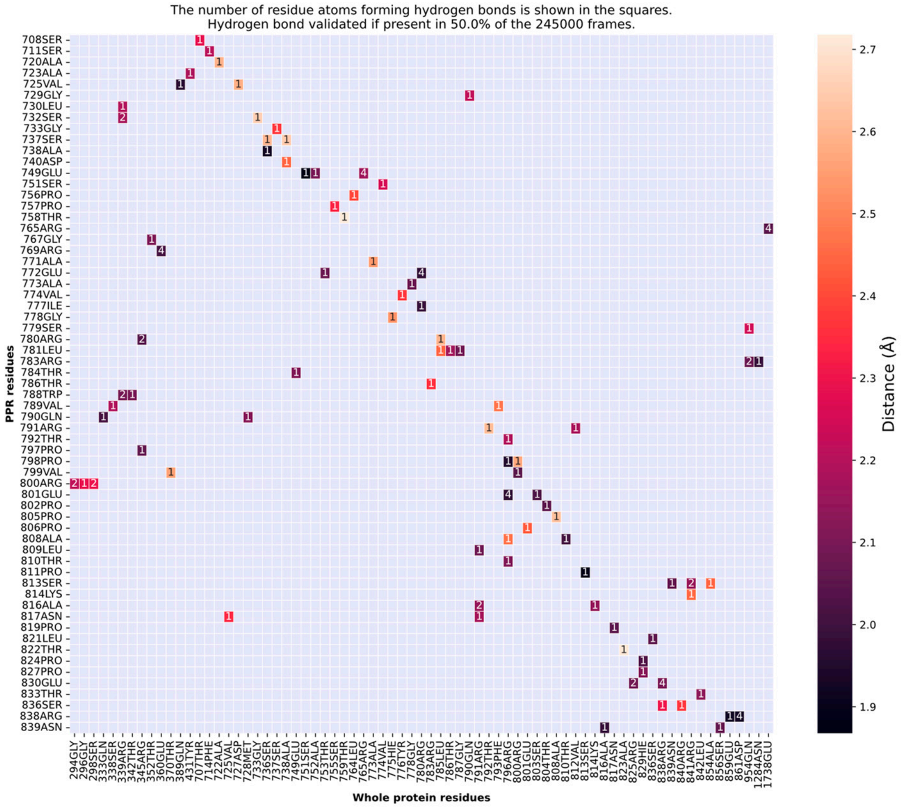

Hydrogen bonds residues median distance: HEPAC-100 PEBP1 ORF1 PPR vs. whole protein

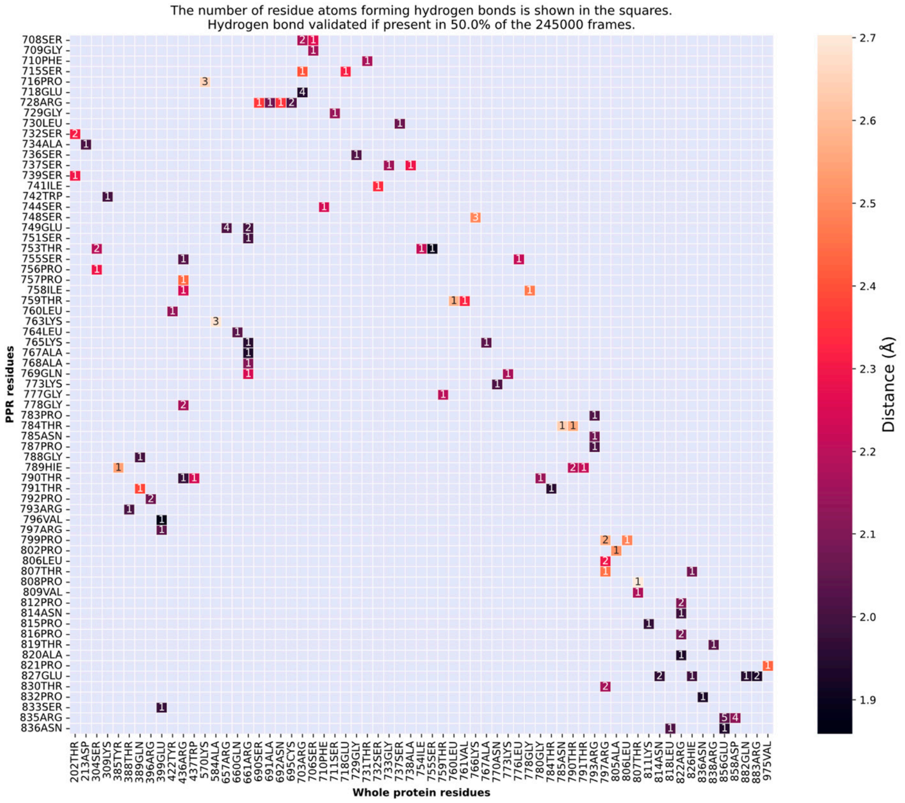

Hydrogen bonds residues median distance: HEPAC-154 KIF1B ORF1 PPR vs. whole protein

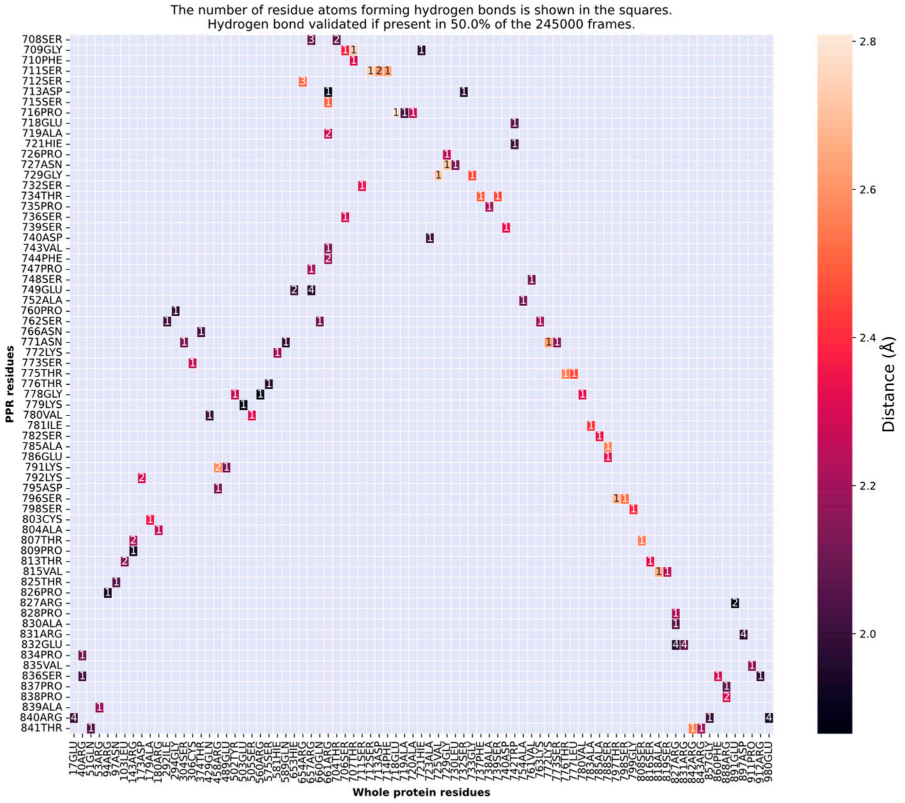

Hydrogen bonds residues median distance: Kernow-C1-p6 RPS17 ORF1 PPR vs. whole protein

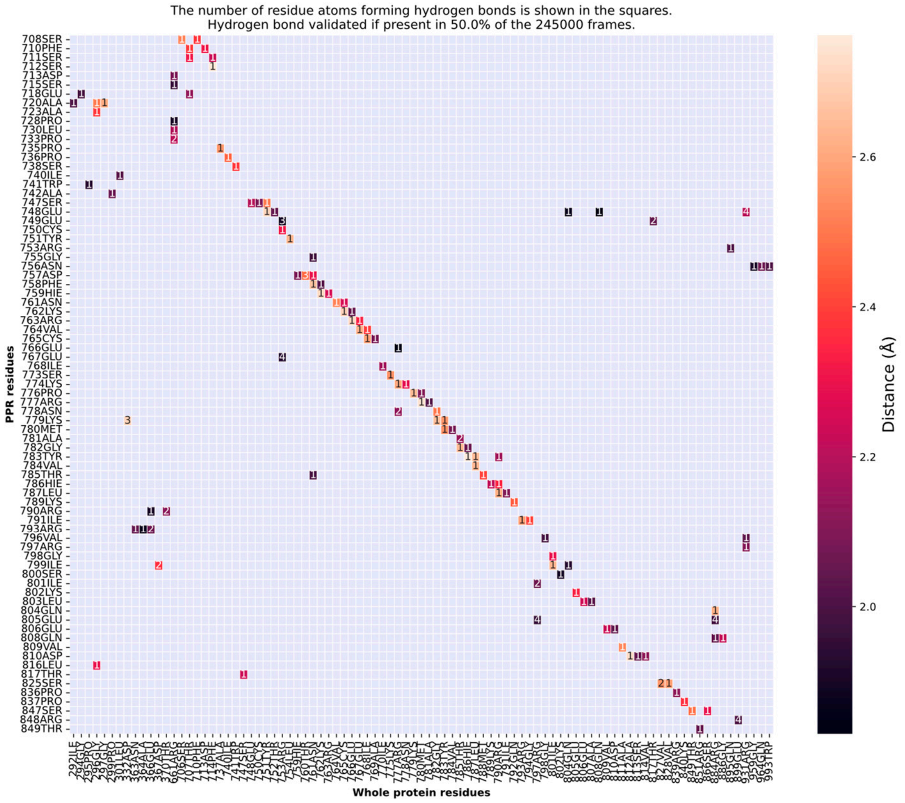

Supplementary Figure S8. The MetY domain Multiple Sequence Alignment, annotated with the number of strains having a contact between the PPR and the MetY domain at each position of the alignment. The grey numbers are positions where the contacts are shared by the insertion strains and the WT strains. The magenta numbers are specific to the insertion strains. The insertions specific contacts increase from the positions 292 to 437, highlighted by black boxes.

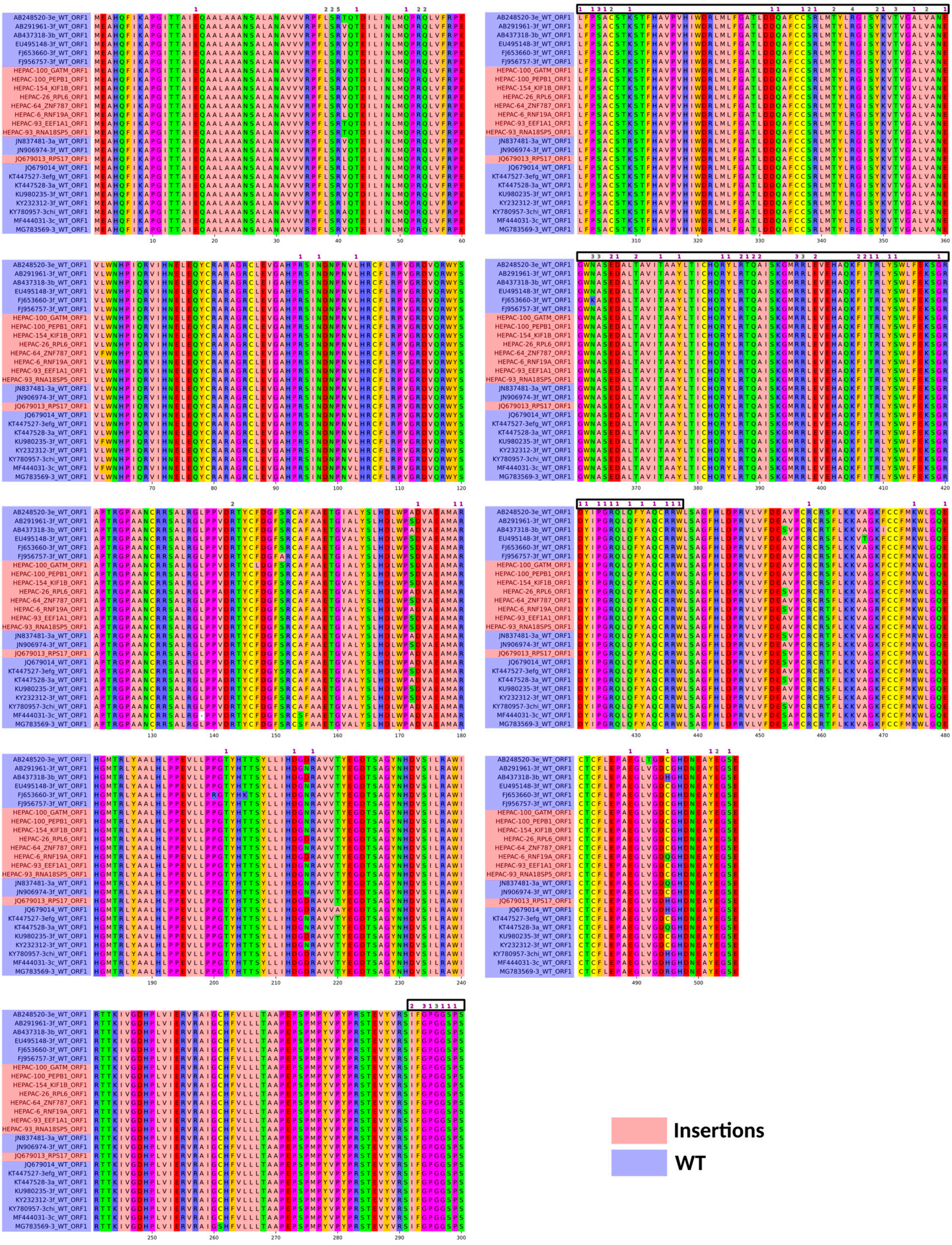

Supplementary Figure S9. Multiple Sequences Alignment of the RdRp and number of hydrogen bonds with the PPR. The RdRp Multiple Sequence Alignment, annotated with the number of strains having a contact between the PPR and the RdRp at each position of the alignment. The grey numbers are positions where the contacts are shared by the insertion strains and the WT strains. The magenta numbers are specific to the insertion strains. The insertions specific contacts increase in two regions from the positions 1544 to 1587 and from positions 1653 to 1676, highlighted by black boxes.

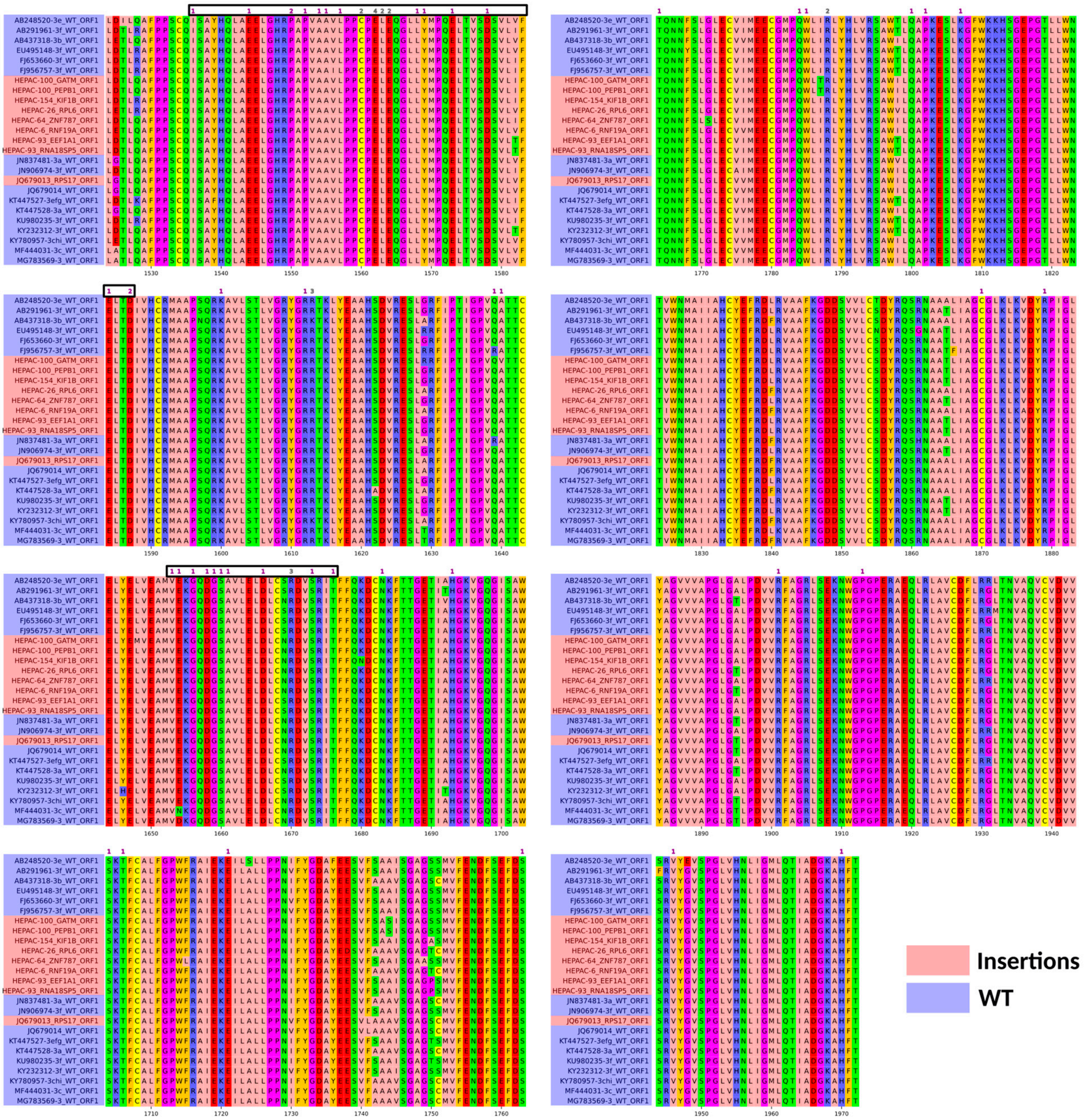

Supplementary File S1. The HEV pORF1 amino acids sequences with insertion events fasta file.

Supplementary File S2. The HEV pORF1 Wild Types amino acids sequences fasta file.
